# From Pharmacophore to Warhead: NAD^+^‐Targeting Triazoles as Mechanism‐Based Sirtuin Inhibitors

**DOI:** 10.1002/anie.202516782

**Published:** 2025-10-30

**Authors:** Florian Friedrich, Marat Meleshin, Niklas Papenkordt, Lena Gaitzsch, Isabel Prucker, Marco Borso, Jan Ruprecht, Christopher Vorreiter, Sabrina Rast, Lin Zhang, Matthias Schiedel, Wolfgang Sippl, Axel Imhof, Henning J. Jessen, Oliver Einsle, Mike Schutkowski, Manfred Jung

**Affiliations:** ^1^ Institute of Pharmaceutical Sciences University of Freiburg Albertstr. 25 79104 Freiburg Germany; ^2^ Department of Enzymology Charles Tanford Protein Center Institute of Biochemistry and Biotechnology Martin‐Luther‐University of Halle‐Wittenberg Kurt‐Mothes‐Straße 3a 06120 Halle Germany; ^3^ Institute of Organic Chemistry University of Freiburg Albertstr. 21 79104 Freiburg Germany; ^4^ Biomedical Center, Medizinische Fakultät Ludwigs‐Maximilians Universität Großhaderner Str. 9 Plangegg‐Martinsried 82152 Germany; ^5^ Department of Medicinal Chemistry Institute of Pharmacy Martin‐Luther‐University of Halle‐Wittenberg 06120 Halle Germany; ^6^ Deutsches Konsortium für Translationale Krebsforschung (DKTK) 79104 Freiburg Germany; ^7^ Institute of Biochemistry University of Freiburg Albertstr. 21 79104 Freiburg Germany; ^8^ Faculty of Synthetic Biology Shenzhen University of Advanced Technology Shenzhen 518107 China; ^9^ Institute of Medicinal and Pharmaceutical Chemistry Technische Universität Braunschweig Beethovenstraße 55 38106 Braunschweig Germany

**Keywords:** Covalent adduct, Drug discovery, Inhibitors, Sirtuins, Triazoles

## Abstract

Sirtuins (SIRTs) are nicotinamide adenine dinucleotide (NAD^+^)‐dependent lysine deacylases linked to key physiological and disease processes. Here, we report a new class of mechanism‐based 1,2,3‐triazole inhibitors that hijack SIRT catalysis by forming stalled triazolium– or triazole–ADP‐ribose (ADPR) adducts derived from the cofactor NAD^+^. These trapped adducts inhibit the enzyme without covalent protein modification, prompting us to term the compounds “Sirtuin Trapping Ligands” (SirTraps). X‐ray crystallography and kinetics, together with mass spectrometry confirming adduct formation both in vitro and in cellulo, reveal that the triazole N3 of peptide‐ and small‐molecule‐based SirTraps triggers nucleophilic attack at C1’ of the nicotinamide riboside moiety of NAD⁺, mimicking the first deacylation step. Adduct formation critically depends on precise triazole positioning within the acyl‐lysine channel and can be tuned through scaffold design, enabling potent and isoform‐selective inhibition. Unlike thiocarbonyl‐based NAD⁺‐targeting SIRT inhibitors, which may suffer from instability and off‐target effects, SirTraps combine high stability, synthetic accessibility, and structural tunability, while demonstrating nanomolar cellular target engagement confirmed by NanoBRET assays. Beyond SIRTs, this inhibition strategy may extend to other NAD⁺‐dependent enzymes, including ADP‐ribosyltransferases, opening new avenues for mechanism‐driven drug discovery.

## Graphical abstract

We report “Sirtuin Trapping Ligands” (SirTraps), mechanism‐based 1,2,3‐triazole inhibitors that hijack sirtuin (SIRT) catalysis to form covalent triazolium– or triazole–ADP‐ribose (ADPR) adducts from NAD^+^. These trapped intermediates stall the enzyme in an inactive state, providing a biocompatible alternative to thiocarbonyl inhibitors and a blueprint for targeting other NAD^+^‐dependent enzymes.

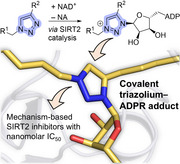

Sirtuin 2 (SIRT2) is a predominantly cytoplasmic, nicotinamide adenine dinucleotide (NAD^+^)‐dependent lysine deacylase involved in key biological processes, including inflammation, genomic stability, and metabolic homeostasis.^[^ ^1^, ^2^, ^3^, ^4^ ^]^ Initially linked to α‐tubulin deacetylation^[^ ^5^ ^]^ and cell cycle regulation,^[^ ^3^ ^]^ SIRT2 also deacetylates histone H4K16Ac,^[^ ^6^ ^]^ p300,^[^ ^7^ ^]^ and NF‐κB,^[^ ^8^ ^]^ and functions as a lysine defatty‐acylase, as demonstrated by its activity on KRas4a,^[^ ^9^ ^]^ linking it to chromatin regulation and cellular stress responses. Mounting evidence implicates SIRT2 in age‐related diseases, such as cancer, neurodegeneration, and metabolic disorders.^[^ ^10^, ^11^, ^12^ ^]^ To dissect its complex biology, diverse inhibitors have been developed, ranging from small molecules to peptide‐based compounds. Among these, “Sirtuin Rearranging Ligands” (SirReals) bind within the acyl‐lysine channel and selectively induce the formation of a unique “selectivity pocket” via their dimethyl pyrimidine group.^[^ ^13^ ^]^ Other inhibitors^[^ ^14^, ^15^, ^16^, ^17^, ^18^ ^]^ and myristoylated lysine (Kmyr) substrates also access this SIRT2‐specific site.^[^ ^19^, ^20^ ^]^ Optimizing SirReals, especially via triazole‐mediated H‐bonding to Arg97, led to potent inhibitors like Mz242 (**1**) and SH10 (**2**) (Figure 1a).^[^ ^21^, ^22^, ^23^ ^]^ SIRTs catalyze lysine deacylation through a distinctive mechanism in which the amide oxygen of the acylated lysine transiently attacks the C1’ of the nicotinamide riboside residue from NAD^+^ (hereafter referred to as C1’ of NAD^+^ ribose), forming a covalent ADP‐ribosylated intermediate. This intermediate is subsequently resolved to yield the deacylated lysine and 2′‐O‐acyl‐ADP‐ribose (Figure S1). Several thiocarbonyl‐containing lysine‐based inhibitors exploit this NAD^+^‐dependent mechanism by forming stalled intermediates, resulting in high affinity and efficacy (Figure 1b).^[^ ^24^, ^25^ ^]^ SIRTs share ADP‐ribosylation chemistry with related enzyme families, including poly(ADP‐ribose) polymerases (PARPs), mono‐ADP‐ribosyltransferases (mono‐ARTs), and ADP‐ribosyl cyclases.^[^ ^26^, ^27^ ^]^

**Figure 1:**
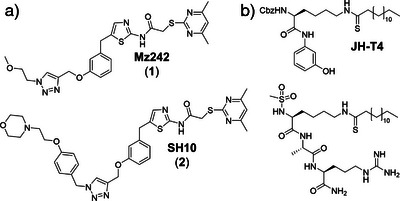
Selected SIRT2 inhibitors. a) SIRT2‐selective SirReal‐triazole inhibitors Mz242 (**1**)^[^ ^21^ ^]^ and SH10 (**2**)^[^ ^22^ ^]^ featuring key H‐bond interactions with Arg97 that enhance potency. b) Representative peptide‐based SIRT2 inhibitors covalently engaging NAD⁺ through a thiomyristoyl warhead forming stalled intermediates.^[^ ^25^, ^28^ ^].^

Building on the enhanced potency of SirReal‐triazoles and the high affinity of long‐chain acylated substrates – such as Kmyr – for SIRT2,^[^ ^19^, ^20^ ^]^ we designed and synthesized a series of TNFα‐derived 1,2,3‐triazole inhibitors, as bioisosteres of amides, based on ornithine and lysine scaffolds to evaluate their inhibitory activity.

Compounds OTi‐1 (**3**, ornithine‐triazolyl inhibitor 1) and LTi‐1 (**4**, lysine‐triazolyl inhibitor 1) mimic acetylated lysine (Kac), whereas OTDi‐1 (**5**, ornithine‐triazolyl‐dodecyl inhibitor 1) and LTDi‐1 (**6**, lysine‐triazolyl‐dodecyl inhibitor 1) mimic Kmyr through their dodecyl extensions, engaging the full acyl‐lysine channel (Figure 2a). Differential scanning fluorimetry (DSF) assays showed no SIRT2 stabilization with 25 µM of **3** or **4**. In contrast, significant melting temperature shifts were observed for **5** and **6** at 10 µM, further enhanced by NAD^+^ addition (Δ*T*_m_: **5** = 9.9 ± 0.0 °C, **6** = 16.3 ± 0.1 °C, Figure 2b), indicating strong SIRT2 stabilization. In a fluorescence‐based deacetylation assay, **5** and **6** reached the assay limit (IC_50_ < 100 nM).^[^ ^29^ ^]^ To better assess inhibitory potency, a more sensitive assay using a fatty‐acylated substrate was employed.^[^ ^30^ ^]^ Compound **6** exhibited an IC_50_ of 28 ± 6 nM, which was further reduced to 3.6  ± 0.6 nM after 15 min of NAD^+^ preincubation, implying cofactor‐assisted binding (Figure 2c). While NAD^+^ preincubation also improved the affinity of compound **5** (IC_50_ = 0.76 ± 0.25 µM, Figure S2C), its potency remained > 200‐fold lower than for **6**. In a jump‐dilution assay, preincubation of SIRT2 with compound **6** and NAD^+^ reduced enzymatic activity to 13% following a 1:100 dilution to 4 nM. Without NAD^+^ preincubation, activity quickly recovered to 72% post‐dilution (Figure 2d). Kinetic analysis using a myristoylated substrate^[^ ^31^ ^]^ revealed a time‐dependent decrease in the reaction rate, characteristic of slow‐binding inhibitors (Figure 2e). Fitting of the progress curves to a two‐step inhibition model supported an NAD^+^‐targeting covalent mechanism (Figure 2f, *k*_inact_ = 0.19 ± 0.01 min^−1^ (3.17 ± 0.17 × 10^−3^ s^−1^), *K*_I_^app^ = 24.7 µM). Although only a moderately tight‐binding SIRT2 inhibitor, compound **6** displays a rapid inactivation rate (*t*_1/2_ = 3.40 min), similar to thiocarbonyl‐based inhibitors, resulting in high potency.^[^ ^24^, ^25^, ^32^ ^]^

**Figure 2:**
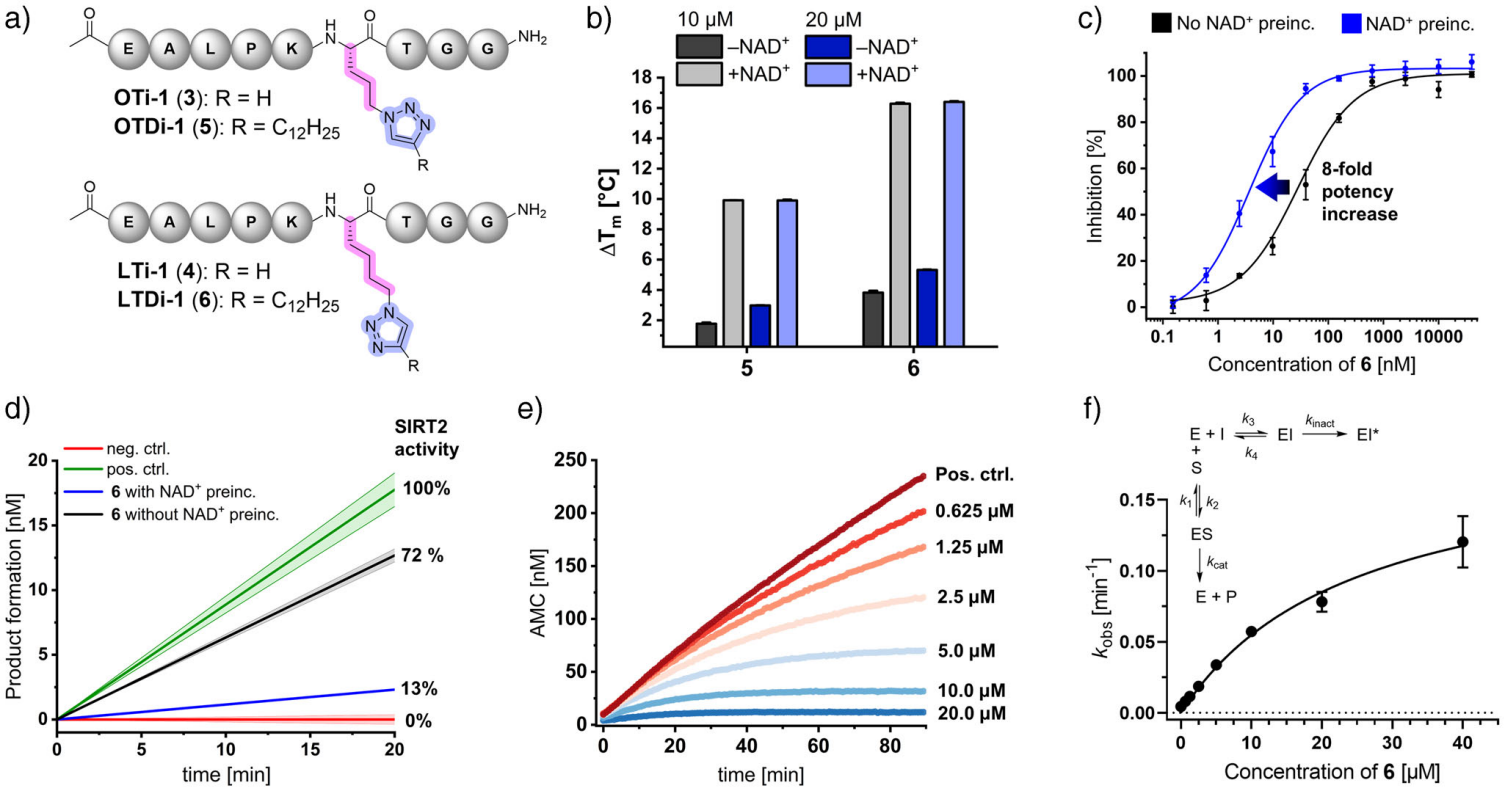
Chemical structures and biochemical profiling of peptide‐based triazole SIRT2 inhibitors. a) Structures of OTi‐1 (**3**), LTi‐1 (**4**), OTDi‐1 (**5**) and LTDi‐1 (**6**). b) Differential scanning fluorimetry showing increased SIRT2 thermal stability for compounds **5** or **6** in the presence of NAD^+^. c) IC_50_ curves for SIRT2 demyristoylation inhibition by compound **6**. Preincubation with NAD^+^ for 15 min enhances the inhibitory potency of **6**. d) Jump‐dilution assay with **6**, revealing persistent inhibition after NAD^+^ preincubation for 30 min (blue curve). e) Time‐dependent curvature of enzymatic progression curves at varying concentrations of compound **6**, consistent with slow‐binding kinetics. f) Two‐step binding model of slow‐binding inhibitors and concentration‐dependent *k*_obs_ plot for **6**, where *k*_obs_ represents the apparent first‐order rate constant of enzyme inactivation.

To elucidate the molecular basis of this unexpected mode of inhibition, co‐crystallization experiments were performed with SIRT2 and compounds **5** and **6**, both in the absence and presence of NAD⁺. Structures without NAD^+^ – SIRT2–**5** (Figure S4A, PDB 9S23, 2.30 Å resolution) and SIRT2–**6** (Figure S4B, PDB 9S25, 2.10 Å resolution) – revealed binding modes closely resembling natural Kmyr substrates (Figure S4C).^[^ ^19^, ^20^ ^]^ In the presence of NAD^+^, covalent adducts between ADP‐ribose (ADPR) and the inhibitors were observed: SIRT2–[**5**–ADPR] (Figures S5A and S5C, PDB 9S24, 2.10 Å resolution) and SIRT2–[**6**–ADPR] (Figures 3a, S5B, and S5D, PDB 9S26, 2.30 Å resolution), consistent with a mechanism‐based inhibition. This reaction mimics the first step of SIRT‐catalyzed deacylation, where the triazole N3‐atom functionally replaces the amide oxygen of the natural substrate attacking the C1’ of NAD^+^ ribose, leading to nicotinamide (NA) release and formation of a stabilized triazolium intermediate (Figure 3c). The intermediate remains trapped in the SIRT2 active site, prompting us to term this novel inhibitor class “Sirtuin Trapping Ligands” (SirTraps). The conserved His187 forms T‐shaped π–π and cation–π interactions with the triazolium ring and participates in a hydrogen‐bonding network with the 2′‐ and 3′‐OH groups of the ribose, further stabilized by backbone and side chain contacts with Gln167 (Figure 3b). These findings suggest that His187 may polarize the triazole to facilitate nucleophilic attack and undergo a conformational adjustment during adduct formation to optimize ADPR interactions. Consistent with the native mechanism, Phe235 adopts a gatekeeper role at the acyl‐lysine channel entrance, shielding the electrophilic C1’ center from solvent‐induced hydrolysis (Figures S5C and S5D).

**Figure 3:**
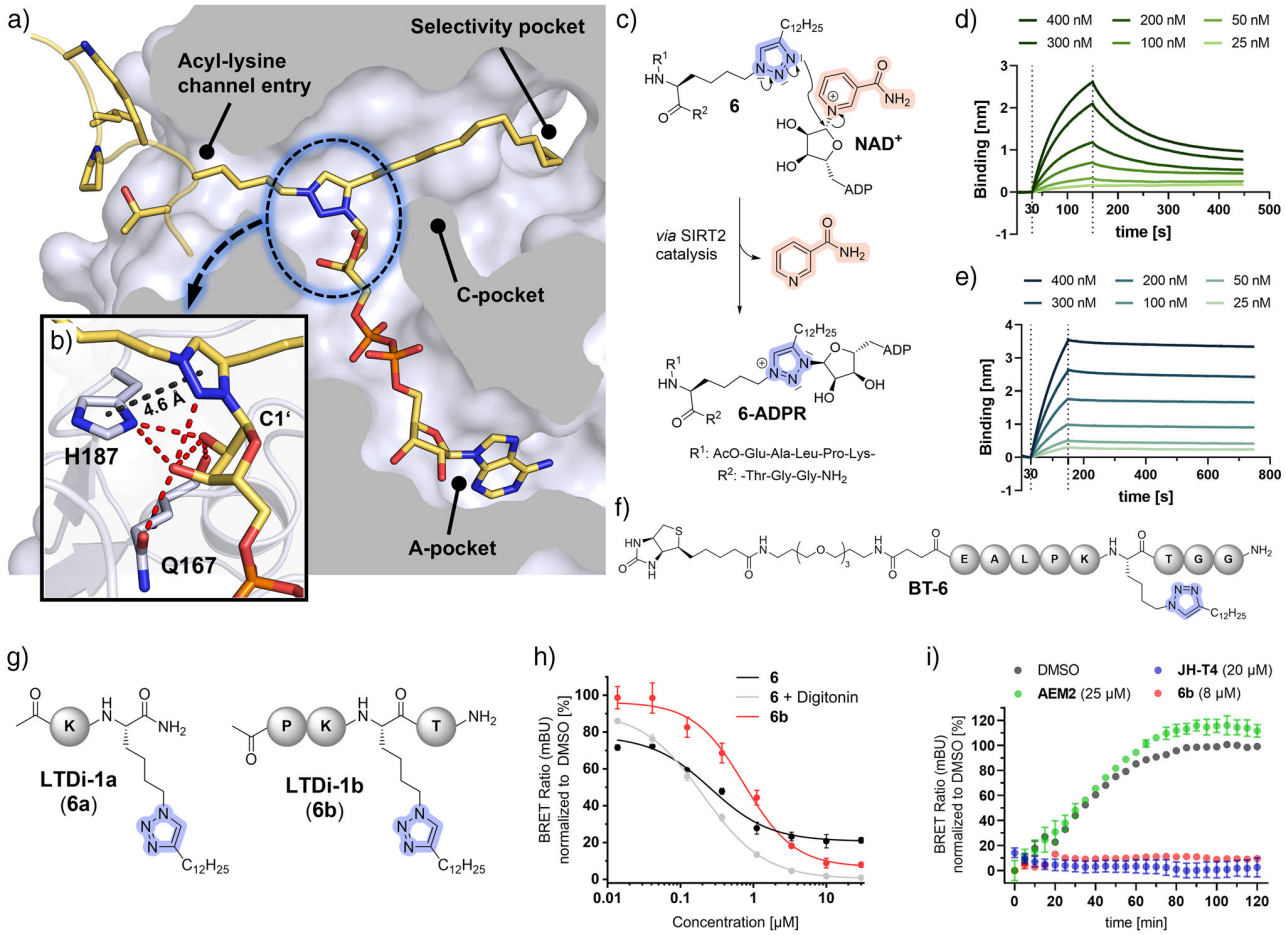
Crystal structure of the SIRT2–[**6**–ADPR] complex (PDB 9S26), proposed inhibition mechanism, biophysical measurements using biolayer interferometry, compound modification and cellular target engagement data. a) Cross‐sectional view of the SIRT2–[**6**–ADPR] complex (**6**–ADPR: yellow–orange). The covalent **6**–ADPR adduct is trapped within the enzyme, with the dodecyl chain of compound **6** extending deep into the hydrophobic acyl‐lysine channel and reinforcing binding via van der Waals interactions with surrounding hydrophobic residues. b) The triazolium cation is stabilized by π–π and cation–π interactions with His187, along with hydrogen bonding from the ribose 2′‐ and 3′‐OH groups to Gln167 and His187, locking **6**–ADPR in place. c) Proposed mechanism: The triazole N3 of compound **6** performs an S_N_2‐type nucleophilic attack on the C1’ of NAD^+^ ribose, displacing nicotinamide and forming a covalent triazolium–ADPR adduct, thereby stalling enzyme activity. d). BLI analysis of SIRT2 at varying concentrations (25–400 nM) following 1 h preincubation with **BT‐6** (500 nM), demonstrating rapid dissociation. e) BLI analysis of SIRT2 at varying concentrations (25–400 nM) following 1 h preincubation with **BT‐6** (500 nM) and NAD^+^ (500 µM), revealing tight and slowly dissociating binding. f) Chemical structure of biotinylated probe **BT‐6**. g) Structures of truncated analogues of compound **6**: **6a** and **6b**. h) NanoBRET‐based assessment of cellular target engagement of NanoLuc–SIRT2 in HEK293T cells using 2 µM tracer. Compound **6** shows modest engagement without digitonin (black) and improved engagement with digitonin (grey), indicating limited membrane permeability, whereas compound **6b** (red) achieves full target engagement even in the absence of digitonin. i) Kinetic NanoBRET washout experiments in HEK293T cells after 2 h preincubation with **AEM2**, **JH‐T4**, or **6b**, followed by a 2 h washout. Only the mechanism‐based inhibitors **JH‐T4** and **6b** retained full inhibition throughout, whereas the inhibition of **AEM2** fades with washout over 2 h.

Clear electron density for the **5**–ADPR and **6**–ADPR adducts confirms their entrapment in the active site (Figures S5A and S5B). Structural comparison of SIRT2–[**5**–ADPR] and SIRT2–[**6**–ADPR] reveals the origin of their differing affinities. In **6**, the triazole is ideally positioned closer to the C1’ of NAD^+^ ribose (2.8 versus 3.5 Å for **5**), favoring nucleophilic attack with minimal steric hindrance (Figure S4D). In contrast, **5** needs to adopt a deeper position, leading to suboptimal geometry and too close contacts with the acyl‐lysine channel entrance (Phe235–Leu239, Figure S5E). These features support tighter binding of **6**–ADPR and underscore the importance of triazole placement for effective NAD⁺ targeting.

Given that the adduct is not covalently bound to the protein but rather to the cofactor‐derived ADPR, we next examined its dissociation kinetics. To this end, a biotinylated analogue of compound **6** (**BT‐6**; Figures 3f and S11) was synthesized and biolayer interferometry (BLI) measurements were performed. SIRT2 was incubated with **BT‐6** in the presence or absence of NAD^+^ for 1 h, followed by immobilization onto streptavidin‐coated biosensors to assess dissociation behavior. Without NAD^+^, rapid dissociation was observed (*k*_off_ = 7.1 × 10^−3^ ± 1.4 × 10^−4^ s^−1^, Figure 3d), consistent with a fast on/off mechanism. Preincubation with NAD^+^ markedly slowed dissociation (*k*_off_ = 2.6 × 10^−4^ ± 8.5 × 10^−6^ s^−1^, Figure 3e), accounting for the high potency of compound **6**.

To probe the role of peptide length in SIRT2 binding, we synthesized truncated variants LTDi‐1a (**6a**) and LTDi‐1b (**6b**) with shortened peptides (Figure 3g). Despite a 10–15‐fold increase in affinity upon NAD^+^ preincubation, truncation led to moderately reduced potency, compared to compound **6** (**6a**: IC_50_ = 36 ± 2 nM; **6b**: IC_50_ = 13 ± 1 nM, Figures S2D and S2E). These results underscore the contribution of peptide backbone contacts with surface‐exposed SIRT2 residues (Figure S5F), consistent with observations in other isoforms such as SIRT5.^[^ ^33^ ^]^ Cellular target engagement was assessed in HEK293T cells stably expressing a NanoLuc–SIRT2 fusion protein using a NanoBRET assay with compounds **6** and **6b**.^[^ ^22^ ^]^ Although compound **6** showed high in vitro potency, it exhibited only modest cellular activity and did not reach full inhibition (EC_50_ = 340 ± 90 nM, *K*_i_ calculated using the Cheng–Prusoff equation^[^ ^34^ ^]^ = 38 ± 10 nM). Co‐treatment with digitonin markedly enhanced target engagement (EC_50 _= 160 ± 20 nM, *K*_i_ = 18 ± 2 nM), indicating limited membrane permeability. The truncated analogue **6b** displayed slightly higher EC_50_ values (730 ± 10 nM, *K*_i_ = 81 ± 1 nM) but achieved complete target engagement without digitonin treatment, suggesting improved cellular uptake (Figure 3h). In a kinetic NanoBRET washout experiment, **6b** retained full inhibition after washout at 8 µM (10 × EC_50_), comparable to the mechanism‐based thiomyristoyl inhibitor **JH‐T4** ^[^ ^28^ ^]^ (20 µM, EC_50_ = 1.9 ± 0.2 µM^[^ ^22^ ^]^). In contrast, the conventional SIRT2 inhibitor **AEM2** ^[^ ^35^ ^]^ (EC_50_ = 5.2 ± 0.2 µM^[^ ^22^ ^]^) completely lost activity at 25 µM (5 × EC_50_; maximum concentration limited by solubility, Figure 3i). These results indicate that the mechanism operates in the cellular environment and that adduct formation enhances target residence time, leading to sustained inhibition.

To determine isoform selectivity, all compounds were tested against SIRT1 and SIRT3, the closest SIRT2 homologs. Given catalytic core conservation, peptide‐based inhibitors often retain activity against isoforms.^[^ ^25^, ^36^ ^]^ Accordingly, most compounds inhibited SIRT1 and SIRT3 deacetylation with nanomolar affinity (Table 1, Figures S8–S10).

**Table 1:** Summary of selectivity results for SirReal‐triazoles and peptide‐based triazole inhibitors across SIRT1‐3 isoforms using fluorescence‐based deacetylation (ZMAL‐assay, 37 °C) with an assay limit of 0.10 µM. Inhibition of SIRT2 demyristoylation was also tested (BSA‐Assay, 25 °C); n.i.: no inhibition.

Compound	SIRT1 (deac.) IC_50_ [µM]	SIRT2 (deac.) IC_50_ [µM]	SIRT2 (demyr.) IC_50_ [µM]	SIRT3 (deac.) IC_50_ [µM]
**1**	n.i.	0.12 ± 0.01	0.66 ± 0.10	n.i.
**2**	n.i.	0.12 ± 0.01	0.05 ± 0.01	n.i.
**5**	14.2 ± 1.5	< 0.10	1.94 ± 0.51	3.3 ± 0.2
**6**	< 0.10	<< 0.10	0.03 ± 0.01	0.13 ± 0.01
**6a**	0.23 ± 0.02	< 0.10	0.46 ± 0.04	8.2 ± 0.4
**6b**	0.10 ± 0.02	<< 0.10	0.11 ± 0.03	0.40 ± 0.03
**7**	n.i.	7.8 ± 2.1	> 10	n.i.

Encouraged by the peptide‐based SirTraps, we next addressed cellular permeability and selectivity by shifting to triazole‐based small molecule inhibitors. The well‐established, SIRT2‐selective SirReal‐triazoles served as a suitable platform.^[^ ^21^, ^22^, ^23^ ^]^ We evaluated two representatives: Mz242 (**1**), bearing a small methoxyethyl‐substituted triazole, and SH10 (**2**), featuring a bulky phenoxyethylmorpholine moiety extending beyond the acyl‐lysine channel entrance (Figure 1 and Table 1). Both were tested in DSF assays with and without NAD^+^. Upon NAD^+^ addition, compound **2** showed only minimal difference in SIRT2 melting temperature compared to NAD^+^ exclusion (Δ*T*_m_ = 0.5 °C), suggesting no adduct formation, consistent with kinetic data (Figure S3B). In contrast, compound **1** triggered a pronounced NAD^+^‐dependent thermal stabilization of SIRT2 (Figure S12B).

To investigate NAD⁺‐dependent binding behavior, we crystallized SIRT2 with NAD^+^ and compounds **1** or **2**. Compound **2** did not show NAD^+^ binding (Figure S6B, PDB 9S20, 1.50 Å resolution). In contrast, the SIRT2–[**1**–ADPR] structure (Figure 4a, PDB 9S1Z, 1.10 Å resolution) revealed a triazolium–ADPR adduct, closely resembling the **5**–and **6**–ADPR adducts (Figures S6A, S6H, and S12C). Compared to the previously reported NAD^+^‐free SIRT2–**1** structure (PDB 8OWZ),^[^ ^23^ ^]^ the triazole of **1** is repositioned toward the C1’ of NAD^+^ ribose to allow covalent bond formation. Notably, adduct formation induces a shift in the Zn^2+^‐binding domain from an open to a closed conformation, suggesting that SIRT2 recognizes **1**–ADPR as a catalytically engaged intermediate (Figure S6E). In SIRT2–[**1**–ADPR], the cofactor‐binding loop (residues 90–110) adopts an extended conformation, whereas in SIRT2–**2**, Arg97 partially occludes the NAD^+^ pocket, likely stabilizing the loop and preventing NAD^+^ binding (Figure S6G).

**Figure 4:**
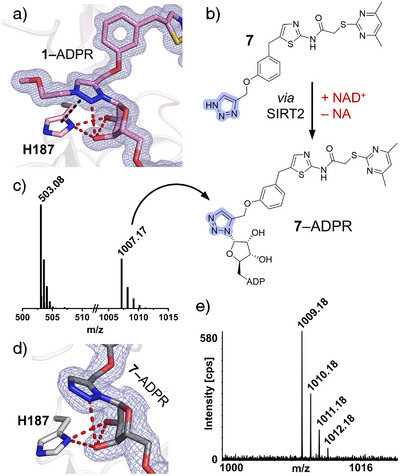
Structural and CE‐ESI‐MS evidence of SirReal‐triazole–ADPR adduct formation in vitro and in HEK293T cells. a) Close‐up of the SIRT2–[**1**–ADPR] complex (PDB 9S1Z) highlighting interactions between the triazolium moiety, His187 and the ADPR ribose moiety. The 2*F*_o_ *–F*_c_ map is depicted as blue mesh and contoured at 1.0*σ*. b) Chemical structure of compound **7** and formation of **7**–ADPR through SIRT2‐catalyzed reaction with NAD^+^. c) CE‐MS data confirming in vitro formation and solution stability of the **7**–ADPR adduct, analyzed in negative mode (*m/z* = 503.08 [2^−^], 1007.17 [1^−^]). d) Close‐up of the SIRT2–[**7**–ADPR] complex (PDB 9S22) showing a shifted orientation of His187 and the triazole of **7**, which disrupts π–π stacking. The 2*F*_o_ *–F*_c_ map is depicted as blue mesh and contoured at 1.0*σ*. e) CE‐MS analysis of **7**–ADPR extracted from HEK293T cells overexpressing NanoLuc–SIRT2, analyzed in positive mode. The singly charged molecule (*m/z* = 1009.19 [M + H]^+^) showed identical migration time and fragmentation pattern to the in vitro reference (Figure S23).

To test this, classical and co‐solvent molecular dynamics (MD) simulations were performed. Compound **1** showed lower interaction occupancy with the cofactor‐binding loop and higher ligand atom fluctuations (RMSF), indicating weaker interactions of its methoxyethyl‐triazole moiety compared to **2** (Figure S14). In classical MD, the ADPR subpocket remained partially closed for both ligands. In co‐solvent MD using xenon probes to open the cofactor binding pocket, the SIRT2–**2** complex retained limited NAD^+^ pocket accessibility, whereas inhibitor **1** induced a shift toward larger pocket volumes, supporting a model where reduced loop stabilization facilitates NAD^+^‐binding and **1**–ADPR formation (Figure S15).

The discovery of a covalent NAD^+^‐targeting mechanism for both peptide‐ and SirReal‐based 1,2,3‐triazole inhibitors was unexpected as disubstituted triazoles are generally poor nucleophiles and the resulting triazolium cation was considered rather reactive and unstable in biological systems. We synthesized the mono‐substituted 1,2,3‐triazole LG023 (**7**, Figure 4b), hypothesizing that removing the alkyl group on the ring nitrogen would enhance nucleophilicity and yield a more stable, uncharged N‐heterocyclic adduct. Structural analysis revealed the formation of the SIRT2–[**7**–ADPR] complex (Figures 4d and S6D, PDB 9S22, 1.95 Å resolution), nearly identical to **1**–ADPR (Figure S6F). To confirm adduct stability in solution, SIRT2 was incubated with NAD^+^ and either **1** or **7**. CE‐ESI‐MS following thermal denaturation confirmed release of **7**–ADPR (Figures 4b,c, and S21), whereas **1**–ADPR was undetectable. When MeOH was used as denaturant, elevated MeO–ADPR levels were observed, consistent with nucleophilic attack of MeOH at the C1′ ribose of **1**–ADPR (Figure S22). These results implicate enhanced solution reactivity as the reason for failed detection of **1**–ADPR. To assess whether the **7**–ADPR adduct also forms in cells, HEK293T cells overexpressing a NanoLuc–SIRT2 fusion protein were incubated with 20 µM of **7** for 3 h. Following lysis and extraction, CE‐ESI‐MS/MS confirmed formation of **7**–ADPR in cellulo (Figures 4e and S23), demonstrating that the reaction mechanism operates in the cellular environment and providing the basis for future cellular studies. Despite improved stability in solution, **7**‐ADPR showed reduced SIRT2 affinity (Table 1, Figures S12B and S13C), likely due to loss of π–π and cation–π interactions between the neutral triazole and His187, weakening active site stabilization (Figure 4d).

In conclusion, durable adduct formation relies on i) close positioning of the triazole moiety near the C1’ of NAD^+^ ribose (≤ 3.0 Å) and ii) stabilization of the inhibitor within and around the acyl‐lysine channel without restricting cofactor loop flexibility. Compound **6** meets both criteria, resulting in slow‐binding kinetics and high affinity whereas truncated analogs (**6a**, **6b**) and SirReal‐triazole inhibitors (**1**, **7**) show reduced effectiveness in vitro. Nonetheless, compound **1** and **6b** retain notable cellular target engagement in NanoBRET, with EC_50_ values of 2.7 ± 0.1 µM (*K*_i_ = 300 ± 10 nM) and 0.73 ± 0.01 µM (*K*_i_ = 81 ± 1 nM), respectively, and pronounced α‐tubulin hyperacetylation for compound **1**.^[^ ^22^ ^]^

To explore broader relevance beyond SIRT2, SIRT3 was co‐crystallized with **6** and NAD^+^, yielding the SIRT3–[**6**–ADPR] complex (Figure S7A, PDB 9S27, 1.60 Å resolution). Superimposition with SIRT2–[**6**–ADPR] revealed a highly conserved binding mode, in which **6**–ADPR maintains key interactions with His248 and Gln228, corresponding to His187 and Gln167 in SIRT2 (Figures 5, S7B and S7C). However, due to hinge region differences (i.e., Gln142 and Phe143 in SIRT2 versus Asn203 and Tyr204 in SIRT3) the dodecyl chain of **6** cannot engage the SIRT2‐specific selectivity pocket and instead projects outward from the channel (Figure 5), as seen in other SIRT3 structures (e.g., PDB 5BWN^[^ ^37^ ^]^ and PDB 9CBT^[^ ^36^ ^]^). These findings indicate that while the reactive triazole core enables binding across SIRTs, structural divergence at the channel periphery governs isoform selectivity, highlighting a route for tuning triazole inhibitors through targeted modification.

**Figure 5:**
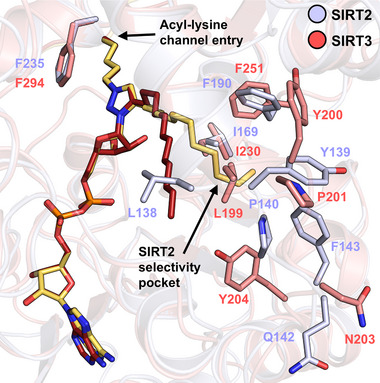
Superimposed crystal structures of SIRT2–[**6**–ADPR] (**6**–ADPR: yellow–orange, SIRT2: blue–white) and SIRT3–[**6**–ADPR] (**6**–ADPR: red, SIRT3: salmon, PDB 9S27) reveal a conserved positioning of the **6**–ADPR adduct. Variations in hinge‐region residues (Gln142/Phe143 in SIRT2 versus Asn203/Tyr204 in SIRT3) result in distinct dodecyl‐chain orientations, with selectivity pocket formation observed only in SIRT2.

In summary, covalent triazolium– and triazole–ADPR adduct formation defines a previously unrecognized mechanism‐based inhibition strategy for SIRTs – distinct from traditional thiocarbonyl warheads or the physiological low‐affinity nicotinamide feedback system.^[^ ^38^ ^]^ While thiocarbonyl‐based inhibitors can achieve high potency and have yielded approved drugs, their broader application is potentially limited by cytotoxicity, metabolic instability, and nonspecific reactivity.^[^ ^39^, ^40^ ^]^ In contrast, the triazole scaffold presented here offers a mechanism‐driven alternative with straightforward synthetic accessibility via click chemistry and reduced off‐target liabilities. Moreover, by modulating the nucleophilic heterocycle and surrounding inhibitor framework, both potency and selectivity can be fine‐tuned. This covalent NAD^+^‐targeting mode of inhibition expands the conceptual framework for SIRT modulation, offering opportunities for selective therapeutic development and potentially extending to other NAD^+^‐dependent enzymes such as PARPs, which are clinically validated targets in oncology. The unprecedented switch of the 1,2,3‐triazole motif, commonly utilized in chemical probes due to its accessibility and perceived low reactivity in biological systems, from a pharmacophore component to a cofactor‐reactive warhead highlights an interesting avenue for further exploration in chemical biology and drug discovery.^[^ ^41^, ^42^ ^]^

## Supporting Information

Additional tables and figures can be found in the Supporting Information. Atomic coordinates and structure factors for SIRT2−[**1**−ADPR] (PDB 9S1Z), SIRT2−**2** (PDB 9S20), SIRT2−**5** (PDB 9S23), SIRT2−[**5**−ADPR] (PDB 9S24), SIRT2−**6** (PDB 9S25), SIRT2−[**6**−ADPR] (PDB 9S26), SIRT2−**7** (PDB 9S21), SIRT2−[**7**−ADPR] (PDB 9S22) and SIRT3−[**6**−ADPR] (PDB 9S27) have been deposited in the Protein Data Bank (www.rcsb.org). The authors will release the atomic coordinates upon article publication.

## Author Contributions

**Florian Friedrich** – Conceptualization; Methodology and Investigation (Protein purification, X‐Ray crystallography, in vitro assays); Data analysis, Visualization and Writing of the original draft. **Marat Meleshin** – Conceptualization; Methodology and Investigation (Peptide inhibitor synthesis); Data analysis. **Niklas Papenkordt** – Methodology and Investigation (Cellular samples and assays); Data analysis. **Lena Gaitzsch** – Methodology and Investigation (Small‐molecule inhibitor synthesis); Data analysis. **Isabel Prucker** – Methodology and Investigation (CE‐MS measurements); Data analysis. **Marco Borso** – Methodology and Investigation (CE‐MS measurements); Data analysis. **Jan Ruprecht** – Methodology and Investigation (BLI measurements); Data analysis. **Christopher Vorreiter** – Methodology and Investigation (MD simulations); Data analysis. **Sabrina Rast** – Investigation (Selectivity assays). **Lin Zhang** – Data analysis. **Matthias Schiedel** – Resources and Funding; Data analysis. **Wolfgang Sippl** – Resources and Funding; Data analysis. **Axel Imhof** – Resources and Funding; Data analysis. **Henning J. Jessen** – Resources and Funding; Data analysis. **Oliver Einsle** – Resources and Funding; Data analysis. **Mike Schutkowski** – Resources and Funding; Data analysis. **Manfred Jung** – Conceptualization; Resources and Funding; Data analysis; Supervision and Project administration; Writing of the original draft. All authors reviewed and approved the final manuscript.

## Conflict of Interests

The authors declare no conflict of interest.

## Supporting information



Supporting Information

Supporting Information

## Data Availability

The data that support the findings of this study are available from the corresponding author upon reasonable request.
