# Supplementary material for: From Pharmacophore to Warhead: NAD+‐Targeting Triazoles as Mechanism‐Based Sirtuin Inhibitors
Source: Angew Chem Int Ed Engl. 2025 Oct 30;64(52):e16782. doi: 10.1002/anie.202516782 (PMC12723449; doi:10.1002/anie.202516782)
Supplement: Supplementary file 1 — Supporting Information [file ANIE-64-e16782-s001.pdf]

Supporting Information

From Pharmacophore to Warhead: NAD^+-Targeting Triazoles as Mechanism-Based Sirtuin Inhibitors

Florian Friedrich,[a] Marat Meleshin,[b] Niklas Papenkordt,[a] Lena Gaitzsch,[a] Isabel Prucker,[c] Marco Borso,[d] Jan Ruprecht,[a] Christopher Vorreiter,[e] Sabrina Rast,[a,f] Lin Zhang,[g,h] Matthias Schiedel,[i] Wolfgang Sippl,[e] Axel Imhof,[d] Henning J. Jessen,[c] Oliver Einsle,[g] Mike Schutkowski,[b] Manfred Jung*[a,f]

*Corresponding author: manfred.jung@pharmazie.uni-freiburg.de

[a] F. Friedrich, N. Papenkordt, L. Gaitzsch, J. Ruprecht, S. Rast, Prof. M. Jung; Institute of Pharmaceutical Sciences, University of Freiburg, Albertstr. 25, 79104 Freiburg, Germany; E-mail: manfred.jung@pharmazie.uni-freiburg.de
[b] Dr. M. Meleshin, Prof. M. Schutkowski; Department of Enzymology, Charles Tanford Protein Center, Institute of Biochemistry and Biotechnology, Martin-Luther-University of Halle-Wittenberg, Kurt-Mothes-Straße 3a, 06120 Halle, Germany
[c] I. Prucker, Prof. H. J. Jessen; Institute of Organic Chemistry, University of Freiburg, Albertstr. 21, 79104 Freiburg, Germany
[d] Dr. M. Borso, Prof. A. Imhof; Biomedical Center, Ludwigs-Maximilians Universität, Großhaderner Str. 9, 82152 Plangegg-Martinsried, Germany
[e] C. Vorreiter, Prof. Wolfgang Sippl; Department of Medicinal Chemistry, Institute of Pharmacy, Martin-Luther-University of Halle-Wittenberg, 06120 Halle, Germany
[f] S. Rast, Prof. M. Jung; Deutsches Konsortium für Translationale Krebsforschung (DKTK), 79104 Freiburg, Germany
[g] Prof. L. Zhang, Prof. O. Einsle; Institute of Biochemistry, University of Freiburg, Albertstr. 21, 79104 Freiburg, Germany
[h] Prof. L. Zhang; Faculty of Synthetic Biology, Shenzhen University of Advanced Technology, 518107 Shenzhen, China
[i] Prof. M. Schiedel; Institute of Medicinal and Pharmaceutical Chemistry, Technische Universität Braunschweig, Beethovenstraße 55, 38106 Braunschweig, Germany

Table of Contents

Page | **Contents**

Page	Contents
**S3**	**Experimental Procedures**
S3	Synthesis
S5	Biochemical Methods
S10	Biological Methods
S11	Computational Methods
**S12**	**Results and Discussion**
S12	Supplementary Figures
S27	Supplementary Tables
S30	Supplementary NMR spectra
S31	Supplementary HPLC spectra
S39	Supplementary References

Experimental Procedures

Synthesis of peptide-based triazole inhibitors

Most of the Fmoc-protected amino acids were purchased from GL Biochem Ltd (Shanghai, China). *N*,*N*-Dimethylformamide (DMF), *N*,*N′*-diisopropylcarbodiimide (DIC), ethyl cyanohydroxyiminoacetate (Oxyma), Fmoc-L-Orn(N_3)-OH (α-Fmoc-δ-azido-L-norvaline), Fmoc-L-Lys(N_3)-OH (α-Fmoc-ε-azido-L-norleucine), Fmoc-TTDS-OH and Rink amide resin (0.65 mmol/g) were purchased from Iris Biotech (Marktredwitz, Germany). (San Diego, USA). *N*,*N*-Diisopropylethylamine (DIPEA), trifluoroacetic acid (TFA) and dichloromethane (DCM) were purchased from Carl Roth (Karlsruhe, Germany). Ethynyltrimethylsilane and 1-tetradecyne were purchased from BLD Pharmatech Ltd. (Shanghai, China) and tris[(1-benzyl-1H-1,2,3-triazol-4-yl)methyl]amine (TBTA) from TCI (Tokyo, Japan). Biotin was purchased from Carbolution Chemicals GmbH (Ingbert, Germany). Acetonitrile (ACN) was obtained from Avantor (Radnor, Pennsylvania, USA). All other chemicals were obtained from Sigma-Aldrich (Saint Louis, USA), unless stated otherwise.

HPLC-MS analysis was performed using a Waters ACQUITY UPLC-MS system (Milford, USA) equipped with a Waters ACQUITY UPLC BEH C18 column (1.7 µM, 2.1 x 50 mm, 30 Å). QTOF spectra were made on Waters Q-Tof Premier (Milford, MA, USA) mass spectrometer. As a mobile phase 0.1 % formic acid in H_2O (solvent A) and 0.1 % formic acid in ACN (solvent B) solutions were used. A typical gradient from 5:95 (v/v) of ACN/H_2O to 95:5 (v/v) of ACN/H_2O over 6 min was used for most runs. Data were analyzed using Waters MassLynx software.

Peptide purification was performed on a Shimadzu LC System with a Phenomenex Kinetex^{TM} 5 µm XB-C18 column (250 x 21.1 mm, 100 Å) with various gradients of 0.05 % TFA in H_2O (solvent A) and 0.05 % TFA in ACN (solvent B).

Solid-phase peptide synthesis

Peptides containing Orn(N_3) and Lys(N_3) were synthesized on Rink amide resin using Fmoc-based solid-phase peptide synthesis on an automated microwave peptide synthesizer Liberty Blue (CEM Corporation). Amino acid couplings were carried out with DIC/Oxyma at 90 °C for 2 min. Fmoc deprotection was accomplished using a 10% (w/v) piperazine solution in a *N*-methylpyrrolidone-ethanol (9:1, v/v) mixture containing 0.1 M 1-hydroxybenzotriazole at 90 °C for 1 min. Biotin (dissolved in DMSO-DMF mixture, 1:1 v/v) was coupled at room temperature for 1 hour, repeated twice. Final *N*-terminal acetylation was performed using an acetic anhydride-DIPEA-DMF mixture (0.5:1:8.5, v/v/v) for 1 h at room temperature.

All peptides were cleaved from the resin using TFA-H_2O (95:5, v/v) for 1 h, repeated twice. TFA was evaporated *in vacuo*, and the crude peptides were dissolved in water and lyophilized.

General procedure for click reaction of azido-containing peptides

Crude azido-containing peptides (from a 0.1 mmol batch synthesis) were dissolved in 1 ml of an ACN-H_2O (1:1) solution. An aliquot of 0.25 ml was transferred to an Eppendorf tube, and the pH was adjusted to 7 using a 10% $NaHCO_3$ solution. The following solutions were then added:

50 µL of 100 mM $CuSO_4$ x $5H_2O$, 50 µL of 100 mM TBTA solution in DMSO, 28 µL of 1-tetradecyne in 0.2 ml of ACN (or 14 µL of neat TMS-acetylene) and 50 µL of 100 mM sodium citrate.

The tubes were sealed with parafilm, placed in a shaker and the mixtures were agitated overnight at 40 °C. The resulting peptide triazole solutions, synthesized using 1-tetradecyne (compounds **OTDi-1**, **LTDi-1, LTDi-1a** and **LTDi-1b**), were directly purified by reverse-phase HPLC.

For triazole derivatives obtained from TMS-acetylene (**OTi-1** and **LTi-1**), the following post reaction treatment was applied:

To each reaction mixture, 20 µL of di-tert-butyl dicarbonate (Boc_2O) and 20 µL of triethylamine were added. The reaction solutions were shaken and monitored by HPLC-MS. This step was introduced to increase the hydrophobicity of the peptides by Boc-protecting the free lysine side chain, thus facilitating HPLC purification.

Subsequently, the reaction mixtures were diluted with an equal volume of water, and the pH was adjusted to 4-6 using acetic acid. Then, 30 mg of solid sodium fluoride was added to remove TMS group from the triazole ring. The tubes were shaken at 40 °C until no TMS-peptide was detected with HPLC-MS (4-5 hours). The resulting Boc-protected peptide triazoles were purified by preparative HPLC.

The lyophilized peptides were dissolved in a small volume of TFA and left for 30 min. TFA was removed under a stream of nitrogen, and the residue was dissolved in water and re-lyophilized to yield the final triazolyl peptides **OTi-1** and **LTi-1**.

Synthesis of SirReal-triazole inhibitors

Mz242 (**1**) & **SH10** (**2**)

The synthesis of compound **1**[1] & **2**[2] was performed as previously described.

LG023 (**7**)

N-(5-(3-((1H-1,2,3-triazol-4-yl)methoxy)benzyl)thiazol-2-yl)-2-((4,6-dimethylpyrimidin-2-yl)thio)acetamide

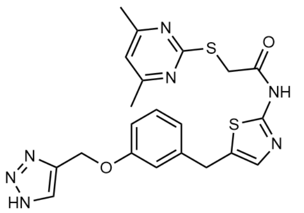

^{1}H-NMR: (400 MHz, DMSO-d_6): δ 12.21 (s, 1H, thiazole-N*H*-CO-), 7.84 (s, 1H, triazole H5), 7.29 – 7.17 (m, 2H, *m*-aryl, thiazole H4), 6.93 (s, 1H, pyrimidine H5), 6.91 – 6.79 (m, 3H, *o*-/*p*-aryl), 5.13 (s, 2H, triazole-CH_2-O-), 4.07 (s, 2H, S-CH_2-CO-), 4.03 (s, 2H, aryl-CH_2-thiazole), 2.27 (s, 6H, pyrimidine CH_3), 1.23 (s, 1H, triazole N*H*). APCI: calc. for $C_{21}H_{21}N_7O_2S_2$ [M-H$^+$]: 466.1, found: 466.1 [M-H$^+$]. HPLC: *t*R = 12.930 min (Method D). UV-purity at 210 nm >99.9%.

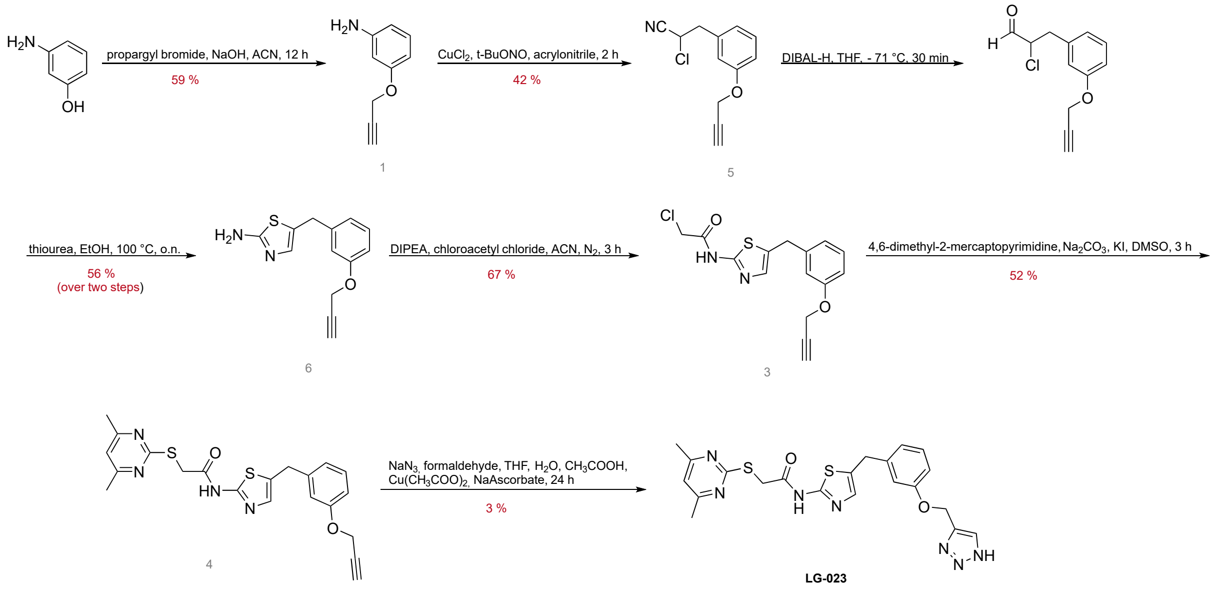

Scheme S1: Synthesis procedure of compound **7**. A mixture of formaldehyde (37 wt%, 0.53 ml, 7.00 mmol), acetic acid (0.06 ml, 1.05 mmol), THF (0.4 ml) and water (0.4 mL) was kept under stirring at room temperature for 15 min. After this time, intermediate **4** (0.30 g, 0.70 mmol) solubilized in THF (0.4 ml) and NaN_3 (0.07 g, 1.05 mmol) were poured into the flask. To this suspension was added a mixture of sodium ascorbate (0.08 g, 0.42 mmol) solubilized in water (0.4 mL) and $CuSO_4$ (0.06 g, 0.35 mmol). The reaction was stirred at room temperature for 24 h and monitored by TLC. The mixture was extracted with DCM and the organic layer was washed with water, dried with Na_2SO_4, filtered and concentrated under reduced pressure. The residue was purified on the preparative HPLC (water/MeCN; 0.05% TFA) to obtain the title product as a white solid (**LG023**, TFA salt, 9 mg, 3%).

JH-T4 & **AEM2**

JH-T4 was kindly provided by Prof. Hening Lin[3] and is commercially available. **AEM2** is commercially available.

Biochemical Methods

Protein expression and purification for assays and crystallization

For assay experiments, strep-tagged human $SIRT1_{134–747}$ and $SIRT2_{56-356}$ were expressed and purified as previously described.[2,4] Briefly, chemically competent *E. coli* BL21 Star (DE3) cells were transformed with pET30S-$hSIRT1_{134–747}$ or pET30S-$hSIRT2_{56–356}$ plasmids. Cell cultures were grown in 2× YT medium (5 g/L NaCl, 16 g/L tryptone, 10 g/L yeast extract) supplemented with 50 µg/mL kanamycin at 37 °C reaching an OD_{600} of 0.6 − 0.7. Protein expression of $SIRT1_{134–747}$ and $SIRT2_{56–356}$ was induced with 1 mM IPTG (isopropyl β-D-1-thiogalactopyranoside), followed by incubation for 16 h at 20 °C. Cells were harvested by centrifugation (15 min, 5000g), resuspended in lysis buffer (100 mM Tris/HCl pH 8.0, 150 mM NaCl, 10% (v/v) glycerol) and lysed via ultrasonication (Branson Digital Sonifier, 70% amplitude, 3 s on/10 s off for 10 min). After centrifugation (1 h, 80,000g), the supernatant was loaded onto a Strep-Tactin Superflow column (5 mL, IBA Lifescience). Bound protein was eluted with lysis buffer supplemented with 5 mM D-desthiobiotin and further purified by size-exclusion chromatography (Superdex S75 26/600, GE Healthcare) using SEC buffer (20 mM HEPES, 150 mM NaCl, pH 7.5). Final protein fractions were concentrated by ultrafiltration, flash-frozen in liquid nitrogen, and stored at –80 °C. Protein purity and identity were confirmed by SDS-PAGE and protein concentration was determined using the BCA assay with BSA as a standard. Human $SIRT3_{118-395}$ was expressed and purified as previously described.[5]

For crystallization experiments, human $SIRT2_{56-356}$ was expressed and purified as previously described with minor modifications summarized hereafter.[5] Cell cultures were grown in 2× YT medium supplemented with 100 µg/mL ampicillin at 37 °C reaching an OD_{600} of 0.6 − 0.8. Protein expression of His_{10}-tagged SIRT2 was induced with 1 mM IPTG, followed by incubation for 16 h at 20 °C. The His_{10}-Tag was cleaved *via* TEV protease in lysis buffer (50 mM Tris/HCl, 500 mM NaCl, 10 % (v/v) glycerol, 0.5 mM TCEP, pH 8.0) at 4 °C for 36 h. Cleaved protein was isolated using a HisTrap HP column (5 mL, GE Healthcare), collecting the flow-through. A final purification step via size-exclusion chromatography (Superdex S75 26/600) in SEC buffer (20 mM HEPES, 150 mM NaCl, pH 7.5) yielded pure SIRT2. The protein was concentrated, flash-frozen, and stored at –80 °C. For purification of His_{10}-tagged SIRT2, TEV cleavage was omitted, and the final size-exclusion chromatography step was carried out directly after HisTrap purification. Protein purity and identity were confirmed by SDS-PAGE and protein concentration was determined using the BCA assay with BSA as a standard. Human $SIRT3_{118-395}$ for crystallography was expressed and purified as previously described.[5]

Differential scanning fluorimetry (DSF) assay

Differential scanning fluorimetry assays were performed in white 96-well plates (Hard-Shell PCR Plates, BioRad, USA) with a total volume of 20 µL per well and a final DMSO concentration of 5% (v/v), following a published protocol.[2,5]

A solution of 10 µL SIRT2 (final concentration of 6.0 µM) and SYPRO Orange (5x, Sigma-Aldrich, Germany) in assay buffer (25 mM Tris-HCl, 150 mM NaCl, 1 mM DTT, pH 8.0) was mixed with 10 µL of compound and incubated at 25 °C and 350 rpm for 5 min. Where applicable, NAD^+ was added to a final concentration of 2.5 mM. Fluorescence intensity was recorded during a temperature gradient of 1°C per 20 s from 25 to 95°C using a real-time PCR machine (C1000 Touch™ Thermal Cycler, CFX96™ Real-Time System, BioRad, USA). Melting temperatures were determined using GraphPad Prism, following a published procedure.[6]

Fluorescence polarization assay (FPA)

Fluorescence polarization (FP) assays were conducted in a black 384-well microplate (OptiPlate-384 F, PerkinElmer) with a total volume of 20 µL per well and a final DMSO concentration of 5% (v/v), following a published protocol.[2,7]

A mixture of 14 µL SIRT2 (final concentration of 200 nM) in assay buffer (50 mM Tris-HCl, 137 mM NaCl, 2.7 mM KCl, 1 mM $MgCl_2$, 1 mg/mL BSA, 0.05% CHAPS, pH 8.0) and 1 µL of compound was incubated at 37 °C and 350 rpm for 10 min. For IC_{50} determination, dilution series of the ligand (20× concentrated in DMSO) were prepared. Where applicable, NAD^+ was added to a final concentration of 500 µM. Negative controls (P_n) used 1 µL Mz242 (**1**) (final concentration of 20 µM), while positive controls (P_p) replaced the inhibitor with 1 µL DMSO. After incubation, 5 µL of SirReal-TAMRA (final concentration of 40 nM, prepared from a 10 mM DMSO stock) was added, followed by further

incubation at 37 °C and 350 rpm for 30 min. Blank controls contained 19 µL of assay buffer and 1 µL DMSO. Fluorescence intensities were measured with an EnVisionTM plate reader (PerkinElmer, optical module – BODIPY TMR FP, excitation filter – FP 531 nm, emission filter 1 – FP p-pol 595 nm, emission filter 2 – FP s-pol 595 nm). Inhibition was calculated using the equation reported below (P_I, P_n, and P_p are FP values of samples, positive control, and negative control, respectively).

$$I = 100\left(1 - \left(\frac{P_I - P_n}{P_p - P_n}\right)\right)\%$$

Determination of IC_{50} values (SIRT1, SIRT2, SIRT3) via fluorescence-based deacetylation assay (ZMAL-Assay)

The fluorescence-based deacetylation assay was conducted in a black 384-well microplate (OptiPlate-384 F, PerkinElmer) with a total volume of 30 µL per well and a final DMSO concentration of 2% (v/v) according to a published procedure.[8] Briefly, a mixture of 10 µL SIRT enzyme ($SIRT1_{134\text{-}747}$/$SIRT2_{56\text{-}356}$/$SIRT3_{118\text{-}395}$, final concentration of 100 nM) in assay buffer (50 mM Tris, 137 mM NaCl, 2.7 mM KCl, 1m $MgCl_2$, 0.01% Tween, 0.05 mg/mL BSA, 0.2 mM TCEP, pH 8.0), 5 µL of substrate (**ZMAL**, final concentration of 10.5 µM), 5 µL of NAD^+ (final concentration of 500 µM) and 10 µL of inhibitor solution (final concentration of 30 µM – 0.014 µM, serial 1:3 dilutions) was incubated at 37 °C and 300 rpm for 4 h. A solution of trypsin (final concentration of 0.5 mg/mL) and nicotinamide (final concentration of 4 mM) was added and incubated at 37 °C and 300 rpm for 30 min to stop the reaction. Samples with 2% (v/v) DMSO instead of inhibitor were prepared as a positive control, and samples with 2% (v/v) DMSO instead of SIRT enzyme were prepared as a negative control. Fluorescence intensity was measured with a microplate reader (λ_{Ex} = 380-10 nm, λ_{Em} = 460 nm, BMG POLARstar Optima, BMG Labtech, Germany). IC_{50} values were then determined using a nonlinear regression fit with Origin Pro 2019 software. All experiments were performed at least in duplicates.

Determination of IC_{50} values (SIRT2) via fluorescence-based demyristoylation assay (BSA-Assay)

The fluorescence-based demyristoylation assay was conducted in a continuous manner in a black 384-well microplate (OptiPlate-384 F, PerkinElmer) with a total volume of 40 µL per well and a final DMSO concentration of 5% (v/v), following a published procedure.[9] A mixture of 14 µL SIRT2 (final concentration of 1 nM) in assay buffer (20 mM Tris, 150 mM NaCl, 5 mg/mL $MgCl_2$, pH 7.8) freshly supplemented with 2 mg/mL of BSA, 14 µL of substrate (**F4**, K_M = 33 ± 5 nM[9], final concentration of 40 nM) and 2 µL of inhibitor solution (final concentration of 100 µM – 0.002 µM, serial 1:2 or 1:4 dilutions) was incubated at 25 °C and 350 rpm for 5 min. Samples with 5% (v/v) DMSO instead of inhibitor were measured as a positive control, and samples with assay buffer and 5% (v/v) DMSO instead of SIRT2 were measured as a negative control. The reaction was started with the addition of 10 µL NAD^+ (final concentration of 500 µM) and the product formation was immediately monitored by the change of the fluorescence intensity at 25 °C for 20 min. For NAD^+ preincubation experiments, instead of the substrate, 10 µL of NAD^+ (final concentration of 500 µM) was added to the solution and incubated at 25 °C and 350 rpm for 15 min. The reaction was started with the addition of 14 µL substrate (final concentration of 40 nM) and the product formation was immediately monitored by the change of the fluorescence intensity at 25 °C every 60 s for 20 min using a microplate reader (λ_{Ex} = 485 nm, λ_{Em} = 530-10 nm, BMG POLARstar Optima, BMG Labtech, Germany).

The activity was normalized using the positive control as 100% and the negative control as 0%. The normalized activity (v/v_0) was plotted as a function of the logarithm of the inhibitor concentration [I], and a nonlinear fit was performed according to the following equation to determine the IC_{50} value.[9]

$$\frac{v}{v_0} = \frac{100}{1 + 10^{[I] - \log IC_{50}}}$$

Jump-dilution assay

The jump-dilution assay was adapted from the continuous fluorescence-based demyristoylaion assay. Compounds were prepared to concentrations of 10× IC_{50} in assay buffer (20 mM Tris, 150 mM NaCl, 5 mg/mL $MgCl_2$, pH 7.8) freshly supplemented with 2 mg/mL of BSA. 0.2 µL of inhibitor solution (20× IC_{50}, 0.1× IC_{50} after jump dilution) was mixed with 0.2 µL of SIRT2 (200 nM, final concentration of 1 nM after jump dilution). After incubation for 5 min, the

solution was diluted 100-fold to a total volume of 40 µL with substrate (final concentration of 40 nM), NAD^+ (final concentration of 500 µM) and DMSO (final concentration of 5 % (v/v)) and transferred to a black 384-well microplate (Optiplate 384 F, PerkinElmer). For NAD^+ preincubation, 0.1 µL of inhibitor solution (40× IC_{50}, 0.1× IC_{50} after jump dilution), 0.1 µL of SIRT2 (400 nM, final concentration of 1 nM after jump dilution) and 0.2 µL of NAD^+ (500 µM, final concentration of 500 µM after jump dilution) were preincubated for 30 min. Afterwards, the solution was diluted 100-fold to a total volume of 40 µL with substrate (final concentration of 40 nM), NAD^+ (final concentration of 500 µM) and DMSO (final concentration of 5 % (v/v)) and transferred to a black 384-well microplate (Optiplate 384 F, PerkinElmer). The change in fluorescence was monitored with a microplate reader (λ_{Ex} = 485 nm, λ_{Em} = 530-10 nm, BMG POLARstar Optima, BMG Labtech, Germany) every 30 s for 20 min. All measurements were performed in triplicate. Reaction rates were obtained from the slope of the fluorescence signal over time and converted to product concentration using calibration curves, assuming equal fluorescence intensities for substrate and product, as described previously.[9]

Kinetic analysis via continuous enzyme demyristoylation inhibition assay

Rate experiments for determination of kinetic parameters were conducted in a continuous manner in a black 384-well microplate (OptiPlate-384 F, PerkinElmer) with a total volume of 40 µL per well and a final DMSO concentration of 2% (v/v), following a published procedure.[8,10] A solution of 10 µL SIRT2 (final concentration of 20 nM) in assay buffer (50 mM HEPES, 100 mM KCl, 0.01% Tween-20, 0.2 mM TCEP, 0.05 mg/mL BSA, pH 7.4) was incubated with 5 µL substrate (**ETDKMyrAMC**, K_M = 1.8 µM, final concentration of 4 µM), 5 µL NAD^+ (final concentration of 400 µM), 10 µL inhibitor (final concentration of 40 – 0.04 µM; serial 1:2-fold dilution) and a 10 µL trypsin solution (final concentration of 20 ng/µL) in assay buffer. In situ fluorophore release was monitored immediately by fluorescence readings recorded every 45 – 60 s for 60 – 90 min at 25 °C using a microplate reader (λ_{Ex} = 485 nm, λ_{Em} = 530-10 nm, BMG POLARstar Optima, BMG Labtech, Germany).

Assay progression curves of product concentration [P] vs. time (t) were fitted to following equation in order to calculate the apparent first-order rate constant for reaching equilibrium (k_{obs}) at each inhibitor concentration.

$$F_t = F_0 + v_s t + \frac{v_i - v_s}{k_{obs}}\left[1 - e^{-k_{obs} t}\right]$$

F_t = time-dependent signal resulting from product formation (in µM, calculated from AU values), F_0 = Y-intercept = background signal at reaction initiation (in µM, calculated from AU values), v_i = initial product formation velocity (in µM/min), v_s = final/steady-state product formation velocity (in µM/min), t = incubation time after enzyme addition (in min), k_{obs} = observed rate of time-dependent inhibition from initial v_i to final v_s (in min^{-1}).

K_I^{app} and k_{inact} were calculated using following equation:

$$k_{obs} = k_{ctrl} + \frac{k_{inact}[I]}{K_I^{app} + [I]}$$

k_{obs} = observed reaction rate (in s^{-1}), [I] = inhibitor concentration (in M) to find k_{ctrl} = rate of nonlinearity in uninhibited control k_{ctrl} (in s^{-1}), k_{inact} = maximum reaction rate (in s^{-1}) and K_I^{app} = apparent inactivation constant (in M).

Biolayer interferometry (BLI)

Biolayer interferometry (BLI) was performed using a BLItz ® system (Pall FortéBio, Menlo Park, USA). SA biosensors were hydrated in assay buffer (20 mM HEPES, 150 mM NaCl, 0.05% BSA, pH 7.5) for 10 minutes prior to use. His_{10}-tagged $SIRT2_{56\text{-}356}$ (25 nM – 400 nM) was mixed and incubated for 1 h with 500 nM **BT-6** in assay buffer. For covalent adduct formation 500 µM NAD^+ was added additionally. After baseline generation, sensors were dipped into samples followed by dissociation in assay buffer. Association was monitored for 120 s, dissociation was monitored for 300 s in absence of NAD^+ and 600 s in presence of NAD^+. Samples were referenced against a control containing only 500 nM **BT-6**. Rate constants were determined by global fitting to a 1:1 binding model using BLItz Pro 1.2 software.

Capillary electrophoresis–electrospray ionization–mass spectrometry (CE-ESI-MS) analysis of adducts formed *in vitro*

Analysis of the enzymatic products were performed using a capillary electrophoresis coupled to mass spectrometry method (CE-MS) described first in the separation of inositol (pyro)phosphates.[11]
CE (Agilent 7100 CE system) was coupled to a time-of-flight mass spectrometry Agilent 6545 system and ionization was performed with a commercially available Agilent Jet Stream (AJS) electrospray ionization (ESI) source. An Agilent liquid coaxial interface was used and the ionization spray was stabilized with a water-isopropanol (1:1 v/v) mixture as sheath liquid. The sheath liquid was pumped with an Agilent 1200 isocratic LC pump, using a 1:100 splitter and resulting in a flow of 1.5 µl/min.
A mixture of $SIRT2_{56\text{-}356}$ (final concentration of 11 mg/mL) in SEC buffer, compound **1** or **7** (3.33 mM of inhibitor with a final DMSO concentration of 3.33 % (v/v)) and NAD^+ (final concentration of 10 mM) were incubated for 90 min and centrifuged for 5 min to remove inhibitor precipitate. Following SIRT2 denaturation at 65 °C for 1 min and centrifugation for 5 min to remove precipitated protein, 20 nL of each sample was injected under 100 mbar pressure for 20 s. Afterwards, a plug of background electrolyte (BGE) was injected at 50 mbar for 2 s. Separation was achieved in a bare fused silica capillary with an internal diameter of 50 µm and a length of 100 cm. Before the first measurement, capillary was activated with sodium hydroxide (1 M) and flushed with water for 10 min. For measurements, a BGE composed of ammonium acetate (35 mM) titrated to pH 9.75 with ammonia solution was used. A voltage of +30 kV was applied to receive a stable CE current of 22 µA. Analytes were detected in negative ionization mode, a capillary voltage of -3000 V and an ESI spray current of 2.1 µA. The nebulizer gas had a pressure of 8 psi and drying gas were set to temperatures of 250 °C and a flow of 8 l/min. Voltages of 140 V, 60 V and 750 V were set for the fragmentor, skimmer and Oct RFV, respectively. Reference standards (TFA anion, $[M\text{-}H]^-$, 112.9855), and (HP-0921, $[M\text{-}H+CH_3COOH]^-$, 980.0163) in the sheath liquid were used for automatic recalibration of spectra with an acquisition rate of 1 spectra per second, scanning over a mass range of 80 to 1700 *m/z*. Peak assignment was done *via* accurate mass and isotopic pattern (**7**-ADPR adduct calc.: 1007.1736, found: 1007.1737).

CE-ESI-MS analysis of adducts formed *in cellulo*

Reagents: $^{15}N_5$-ATP analytical standard, 0.1 N HCl, 0.1 N NaOH, water, 7.5M ammonium acetate solution, 25% ammonia solution, methanol and chloroform were purchased from Merck (Darmstadt, Germany) and were ultrapure or LCMS grade.

Extraction protocol of cell pellets: Cell pellets (see Biological Methods for details) were resuspended in 50 µL of an ice-cold methanol/water (80/20 v/v) solution. 2.5 µL of $^{15}N_5$-ATP (corresponding to 1 nmol) were added in order to normalize signal intensities. Cell pellets were submitted to a sonication cycle using a Bioruptor® Pico for 10 min (30 s ON and 30 s OFF) at 4°C. Extraction was performed by adding an ice-cold mixture of chloroform/methanol/water (200 µL/250 µL/350 µL), strongly vortexed and incubated 30 min on ice. After centrifugation at 14,000 x g for 15 min at 4°C, 500 µL of the upper polar phase were collected, directly dried in a SpeedVac (Eppendorf, Hamburg, Germany) and stored at -70°C. Dried samples were resuspended in 30 µL of water, vortexed and centrifuged at 10,000 x g for 10 min before being placed into the corresponding nanoVial (Sciex, Concord, Ontario, Canada). The in vitro reference sample was prepared as described in the section "CE-ESI-MS analysis of adducts formed in vitro."

CESI-MS analysis: Electrophoretic separation was carried out using a CESI 8000 (Sciex) equipped with a sheathless OptiMS CESI cartridge (30 µm ID x 91 cm bare fused silica capillary) maintained at 25°C coupled to a Sciex 6600 TTOF. Samples were kept in a thermostated tray at 8°C, injected hydrodynamically into the capillary at 2 psi for 30 s (~10 nL) and separated into a 16 mM Ammonium acetate buffer (25% ammonia solution was used to adjust the pH to a value of 9.7). A voltage of 30 kV in normal polarity was applied for 20 min and during the separation, 1 psi of pressure was applied both at the inlet and at the outlet side. Between injections, the capillary was rinsed with 0.1 N NaOH and 0.1 N HCl at 90 psi for 2 min each followed by water at 90 psi for 2 min and finally by the separation buffer at 90 psi for 3 min. The values for gas 1 (GS1), gas 2 (GS2), temperature (TEM) and Declustering Potential (DP) were set at 0, 0, 50°C and 40, respectively. The mass spectrometer operated in positive TOF-MS mode (m/z range 350-1100 Da) using an IonSpray Voltage Spray (ISVF) of 1700V and an accumulation time of 250 ms. The IDA (information dependent acquisition) was used for further identification of the fragments by monitoring the top 10 ions (m/z range of 200-1100 Da) with an accumulation time of 100 ms and rolling collision energy (**7**-ADPR adduct calc.: 1009.19, found: 1009.19). System control and data acquisition were carried out using AB Sciex 32 Karat (v. 10.3) and Analyst® (v. 1.8.1) software.

Crystallization and soaking

Crystallization assays were prepared with the Oryx Nano pipetting robot (Douglas Instruments, East Garston, UK) using the vapor diffusion sitting drop method (MRC 2 Well UVP Plate, SWISSCI, Buckinghamshire, UK) at 20 °C. All $SIRT2_{56\text{-}356}$– and $SIRT3_{118\text{-}395}$–inhibitor complexes were mixed and incubated on ice for 1 h prior to crystallization. To remove precipitates, the solutions were centrifuged 10 min at 4 °C before pipetting.

Crystals of the SIRT2–[**1**–ADPR] complex (14.0 mg/mL SIRT2, 10 mM β-NAD^+, 0.5 mM of compound **1** with 5 % (v/v) final DMSO concentration) formed after three days in wells with 0.2 µL of protein solution and 0.4 µL of reservoir solution containing 24 % (w/v) PEG MME 2000 in 0.1 M Bis-Tris at pH 6.5. Crystals of the SIRT2–**2** complex (18.0 mg/mL SIRT2, 10 mM β-NAD^+, 1.67 mM of compound **2** with 1.67 % (v/v) final DMSO concentration) formed after two days in wells with 0.2 µL of protein solution and 0.4 µL of reservoir solution containing 19 % (w/v) PEG 3350 in 0.1 M Bis-Tris at pH 6.5. Crystals of the SIRT2–**7** complex (11.4 mg/mL SIRT2, 3.33 mM of compound **7** with 3.33 % (v/v) final DMSO concentration) formed after one day via microseed matrix screening using crystals from the SIRT2–**1** complex[12] in wells with 0.3 µL of protein solution and 0.3 µL of reservoir solution containing 32% (w/v) PEG MME 2000 in 0.1 M Bis-Tris at pH 6.5. Initial microcrystals of the SIRT2–[**7**–ADPR] complex (11.4 mg/mL SIRT2, 10 mM β-NAD^+, 3.33 mM of compound **7** with 3.33 % (v/v) final DMSO concentration) not suitable for diffraction experiments formed after two days in wells with 0.2 µL of protein solution and 0.4 µL of reservoir solution containing 19 % (w/v) PEG 3350 in 0.1 M Bis-Tris at pH 6.5. Final crystals of the SIRT2–[**7**–ADPR] complex (11.4 mg/mL SIRT2, 10 mM β-NAD^+, 3.33 mM of compound **7** with 3.33 % (v/v) final DMSO concentration) formed after three days via microseed matrix screening in wells with 0.1 µL of the initial SIRT2–[**7**–ADPR] microcrystal solution, 0.3 µL of protein solution and 0.3 µL of reservoir solution containing 25 % (w/v) PEG 3350 and 0.2 M $MgCl_2$ x $6H_2O$ in 0.1 M Tris at pH 8.5. Crystals of the SIRT2–**5** complex (11.0 mg/mL SIRT2, 5 mM of compound **5** with 2.5 % (v/v) final DMSO concentration) formed after one day in wells with 0.3 µL of protein solution and 0.3 µL of reservoir solution containing 21.5 % (w/v) PEG 3350 in 0.1 M Bis-Tris at pH 6.7. Crystals of the SIRT2–**6** complex (11.0 mg/mL SIRT2, 5 mM of compound **6** with 2.5 % (v/v) final DMSO concentration) formed after one day in wells with 0.3 µL of protein solution and 0.3 µL of reservoir solution containing 31.5 % (w/v) PEG 3350 in 0.1 M HEPES at pH 7.5. Crystals of the SIRT2–[**5**–ADPR] complex (11.0 mg/mL SIRT2, 20 mM β-NAD^+, 5 mM of compound **5** with 2.5 % (v/v) final DMSO concentration) formed after one day in wells with 0.3 µL of protein solution and 0.3 µL of reservoir solution containing 26.5 % (w/v) PEG 3350 in 0.1 M HEPES at pH 7.5. Initial Crystals of the SIRT2–[**6**–ADPR] complex (11.4 mg/mL SIRT2, 10 mM β-NAD^+, 5 mM of compound **6** with 2.5 % (v/v) final DMSO concentration) formed after two days in wells with 0.3 µL of protein solution and 0.3 µL of reservoir solution containing 31.5 % (w/v) PEG 3350 in 0.1 M HEPES at pH 7.5. Larger crystals of the SIRT2–[**6**–ADPR] complex (11.0 mg/mL SIRT2, 20 mM β-NAD^+, 5 mM of compound **6** with 2.5 % (v/v) final DMSO concentration) formed after one day via microseed matrix screening in wells with 0.1 µL of the initial SIRT2–[**6**–ADPR] crystal solution, 0.2 µL of protein solution and 0.4 µL of reservoir solution containing 29 % (w/v) PEG 3350 and 0.1 M HEPES at pH 7.5.
Crystals of the SIRT3–[**6**–ADPR] complex (10.0 mg/mL SIRT3, 15 mM β-NAD^+, 3 mM of compound **6** with 1.5 % (v/v) final DMSO concentration) formed after 2 days in wells with 0.3 µL of protein solution and 0.3 µL of reservoir solution containing 3 M NaCl in 0.1 M Tris at pH 8.5.
All crystals were cryoprotected with a mixture of reservoir solution and 10 % (v/v) 2*R*,3*R*-(–)-butanediol, mounted on a nylon loop and flash-cooled in liquid nitrogen.

Data collection and structure determination

X-ray diffraction data for the SIRT2–[**1**–ADPR] (PDB 9S1Z) complex was collected on the BM07 beamline at the European Synchrotron Radiation Facility (ESRF, Grenoble, France) using a PILATUS 6M detector (DECTRIS, Switzerland). X-ray diffraction data for the SIRT2–**2** (PDB 9S20), SIRT2–**7** (PDB 9S21), SIRT2–[**7**–ADPR] (PDB 9S22) and SIRT2–[**5**–ADPR] (PDB 9S24) complexes were collected on the ID30B beamline at the ESRF using an EIGER2 X 9M detector (DECTRIS, Switzerland). X-ray diffraction data for the SIRT2–**5** (PDB 9S23), SIRT2–**6** (PDB 9S25), SIRT2–[**6**–ADPR] (PDB 9S26) and SIRT3–[**6**–ADPR] (PDB 9S27) complexes were collected on the ID23-2 beamline at the ESRF using an EIGER2 X 9M detector (DECTRIS, Switzerland). The datasets were processed with autoPROC[13] and scaled using Aimless.[14] The structures were solved by molecular replacement with Phaser[15] using the SIRT2–**1** complex (PDB 8OWZ)[12], the SIRT2–TNFαK20myr complex (PDB 4Y6O)[16] and the SIRT3–ADPR complex (PDB 4BN4)[17] as the search model. Models were built and refined iteratively in COOT[18] and either REFMAC[19] or Phenix.refine.[20] Restraints for the ligands were generated with the Grade Web Server (Global Phasing Ltd., UK). Electron density was well-defined for all ligands. Final structures were validated using MolProbity.[21] All data collection and refinement statistics are reported in Table S1 – S3.

Biological Methods

Preparation of HEK293T cells expressing NanoLuc–SIRT2 fusion protein for MS experiments

HEK293T cells stably overexpressing the NanoLuc-tagged SIRT2$_{50\text{-}356}$ fusion protein were used to investigate **7**-ADPR adduct formation by CE-MS. Cells were seeded in 10 cm cell culture dishes (Sarstedt) and allowed to adhere overnight at 37 °C in a humidified atmosphere containing 5% CO_2. On the following day, the culture medium was replaced with fresh medium containing the indicated concentrations of inhibitor. After 3 h of incubation at 37 °C, cells were harvested by trypsinization and collected by centrifugation for 5 min at 500g. The supernatant was discarded, and the resulting cell pellets were immediately snap-frozen in liquid nitrogen and stored at −80 °C until further processing for CE-MS analysis.

NanoBRET assay

The NanoBRET target engagement assay was performed as previously described[2] with the modification that stably transfected HEK293T cells expressing the NanoLuc-tagged SIRT2$_{50\text{-}356}$ fusion protein were used instead of transient transfection. Briefly, HEK293T-NLuc- SIRT2$_{50\text{-}356}$ cells were cultured under standard conditions (37 °C, 5% CO_2) in DMEM supplemented with 10% fetal bovine serum, 2 mM glutamine, and 300 µg/mL hygromycin as a selection marker. For the assay, cells were trypsinized, resuspended in Opti-MEM® reduced serum medium, and adjusted to a concentration of 2×10^5 cells per mL. To determine the affinities of the inhibitors, a final tracer concentration of 2 µM was used. Serially diluted inhibitor and tracer were added to the cell suspension, and 100 µL were seeded into 96-well white, sterile, nonbinding surface plates. To evaluate the cellular permeability of LTDi-1 (**6**), the non-ionic detergent digitonin (50 µg/ml) was added. Plates were incubated at 37 °C with 5% CO_2 for 2 h. For BRET measurements, NanoBRET NanoGlo Substrate (Promega cat. #N1571) was added to the wells according to the manufacturer's protocol.

All measurements were performed using the 2102 EnVision™ Multilabel Reader (PerkinElmer), equipped with a 460 nm filter (donor) and a 615 nm filter (acceptor). The BRET ratio was calculated as the ratio of acceptor to donor signal, and data were normalized to vehicle-treated controls. Apparent intracellular binding affinities (EC_{50} values) were determined by fitting dose-response curves using nonlinear regression analysis in GraphPad Prism. All experiments were conducted in triplicate unless otherwise noted, and results are presented as mean ± standard deviation. K_i values were calculated using the Cheng–Prusoff equation[22] with [Tracer] = 2 µM and $K_{d,app}$(Tracer) = 0.25 µM[2]:

$$K_i = \frac{IC_{50}}{1 + \frac{[Tracer]}{K_{d,app}(Tracer)}}$$

NanoBRET washout assay for determination of target residence time

A NanoBRET-based washout assay was used to assess the target residence time of inhibitors for SIRT2 in living cells. White, flat-bottom 96-well plates (PerkinElmer) were pre-coated with poly-L-lysine (Sigma-Aldrich) to enhance cell adherence. Briefly, each well was treated with poly-L-lysine solution for 5 min at room temperature, followed by removal of the coating solution and air-drying before seeding.

HEK293T cells stably expressing the NanoLuc-SIRT2$_{50\text{-}364}$-fusion target protein were seeded into the coated plates and cultured overnight at 37 °C in a humidified incubator with 5% CO_2. Cells were treated with the indicated inhibitor concentrations and incubated for 2 h at 37 °C to allow compound binding and target engagement.

After incubation, the inhibitor-containing medium was aspirated, and a washout procedure was performed to remove unbound inhibitor. Each well was washed twice by sequential addition and removal of 100 µL Opti-MEM (Thermo Fisher Scientific). Following the final wash, 90 µL Opti-MEM was added per well, followed by 100 µL of 2× Nano-Glo® substrate solution (Promega) and 10 µL of 20× SIRT2-Tracer solution.

BRET measurements were recorded kinetically using a Tecan Spark® plate reader equipped with donor (460 nm) and acceptor (615 nm) emission filters. The BRET ratio was monitored over time to track the re-binding of the fluorescent tracer.

Computational Methods

Protein-ligand complex preparation

The crystallographically obtained protein–ligand complex structures were used for the present computational studies. Additionally, the Sirt2–**1** complex was retrieved from the Protein Data Bank (PDB ID 8OWZ)[12]. All structures were initially prepared using the Protein Preparation Wizard Panel[23,24] accessed via Schrödinger's Maestro.[25] The structures were first preprocessed, which included bond order assignment, the addition of missing hydrogen atoms, zero-order bond formation to metals, the prediction of missing side chains and loops using Prime[24,26,27], ligand state generation by Epik[24,28], and protein termini capping. The hydrogen bond network was optimized at pH 7.0, whereby pK_a predictions by PROPKA were considered. All buffer molecules as well as water molecules with fewer than two hydrogen bonds to non-water residues were deleted. In the final preparation step, the complexes underwent a restrained minimization procedure within the OPLS 2005 force field[29–31], during which the limit of heavy-atom displacement was set to 0.3 Å.

Molecular dynamics simulations

Molecular dynamics (MD) simulations were carried out using the Amber 22 software package.[32] The protein chains were initially prepared by pdb4amber[32] and parameterized according to the ff14SB force field.[33] The Li/Merz 12-6-4 parameter set[34–37] for +2 to +4 ions delivered respective specifications for the zinc ion being a crucial component of SIRT2. Antechamber and Parmchk[32] carried out the ligand preparation while assigning AM1-BCC atomic charges.[38] The ligands were parameterized according to the General Amber Force Field 2 (GAFF2).[39] TLEAP[32] was used to set up the final simulation systems while solvating the complexes with TIP3P[40] water molecules and adding sodium ions for system neutralization. As a solvation box shape, truncated octahedrons were chosen that ensured a minimum distance of 10 Å between the protein surface and box edges. The co-solvent systems were set up in a similar way; prior to adding the water box, a layer of xenon atoms was placed around the protein-ligand complexes so that a target concentration of ~ 150 mM was achieved. The xenon probe atoms were parameterized as described in previous publications[41–43] using a simple Lennard-Jones potential (ε = 2.35 kJ/mol, σ = 0.4063 nm). Prior to the actual MD production stages, the systems underwent two phases of energy minimization. The first phase consisted of 1000 iterations of steepest descent and 2000 steps of conjugate gradient minimization. Meanwhile, the protein-ligand complex was kept rigid using a force constant of 10 kcal*mol^{-1}*Å^{-2}. The second unrestrained minimization phase included additional 2000 iterations of steepest descent and 2000 steps of conjugate gradient minimization. Afterwards, the systems were heated to target temperature (300 K) through 100 ps of MD simulation while applying constant volume periodic boundaries. In the following pressure equilibration phase, the systems were simulated for additional 100 ps at a constant pressure of 1 bar. During heating and pressure equilibration, the above-mentioned force constant was applied to keep the protein-ligand complexes rigid. Finally, each system underwent 100 ns of unrestrained MD simulation at constant pressure conditions using a time step of 2 fs. The Langevin thermostat ensured temperature equilibration and maintenance at 300 K using a collision frequency of 2 ps^{-1}. The SHAKE algorithm[44] as well as the Particle Mesh Ewald (PME) method[45,46] were applied. The nonbonded cutoff distance for long-range electrostatic interactions was set to 10 Å. MD trajectory analysis (RMSD, RMSF, simulation interactions) was carried out using CPPTRAJ[47] and the Simulation Interactions Diagram Panel[48] accessed via Maestro.[25] Generally, RMSD values were calculated considering all heavy atoms of the analyzed molecule. However, the SIRT2 insertion loop (residues 289-304) was neglected in the protein RMSD calculations due to its high flexibility. In the shown simulation interactions diagrams, only interactions with occupancies >33% were considered. Binding site volume calculations were carried out using MDpocket (Fpocket)[49,50], whereby the superimposed ADP-ribosyl substituent extracted from the SIRT2–[**1**–ADPR] complex served as a reference ligand to define the location of the ADPR subpocket.

All 3D structures visualizing the inhibitor binding modes were created using PyMol.[51] Matplotlib[52] and Seaborn[53] were used to generate the plots.

Results and Discussion

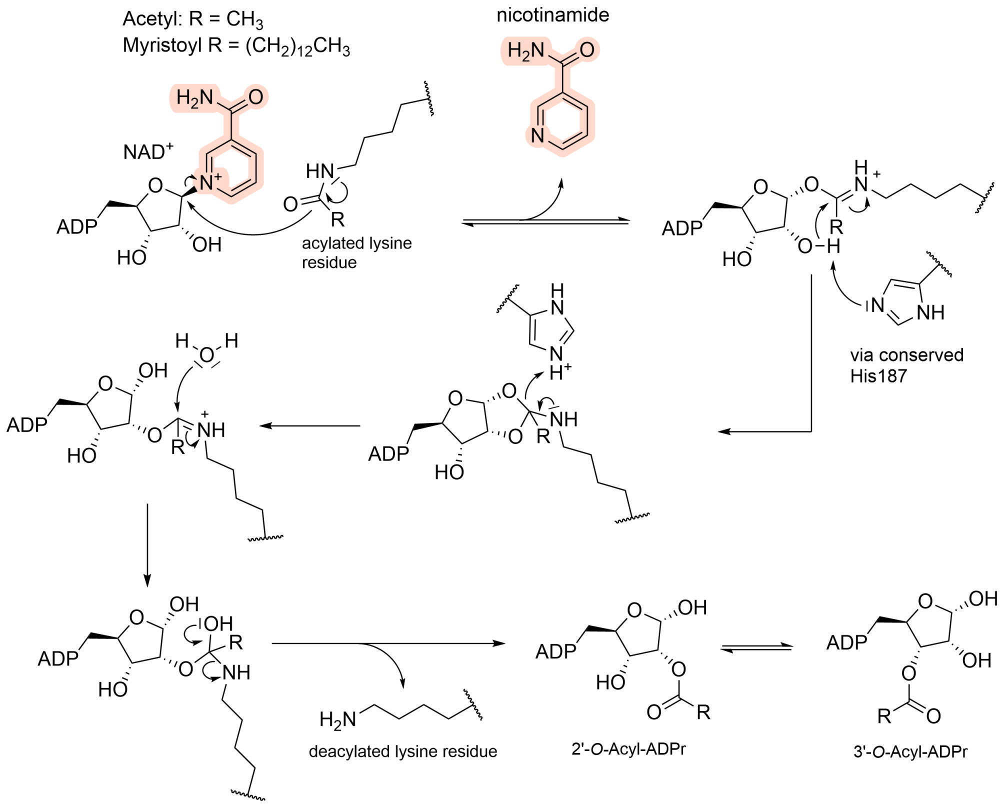

Figure S1. Mechanism of the SIRT2 deacylation process.

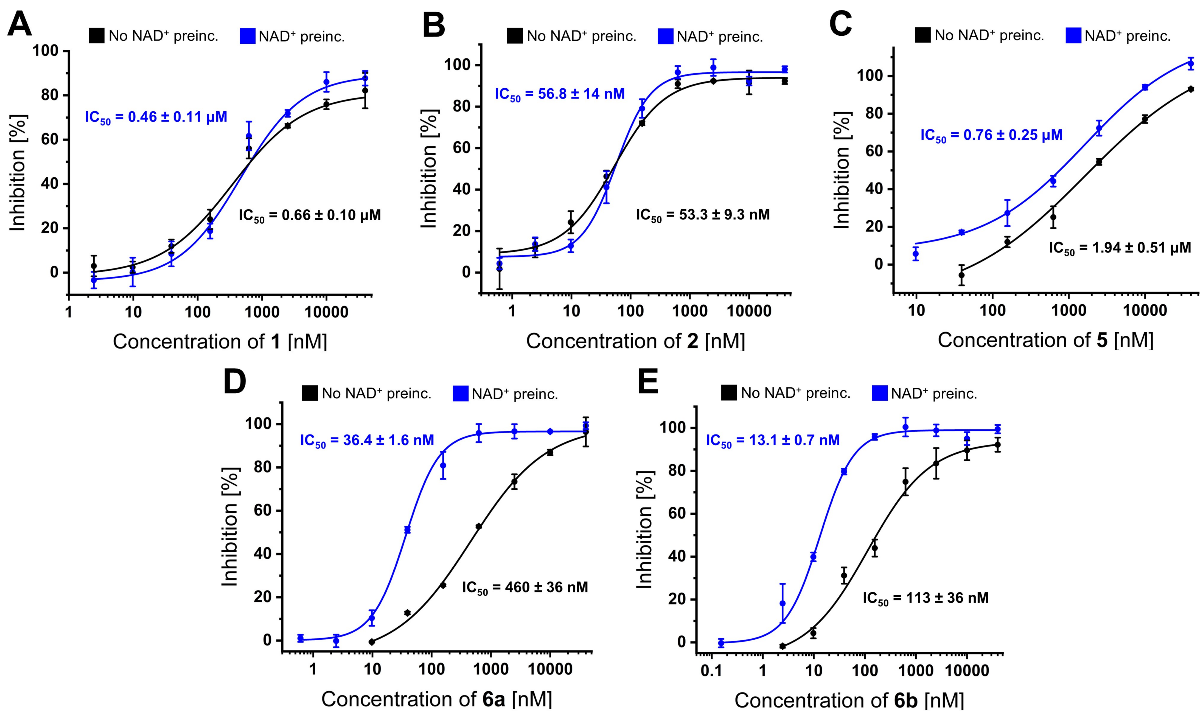

Figure S2. IC_{50} determination using the SIRT2 demyristoylation assay (BSA-Assay) with (blue curves) and without (black curves) NAD^+ preincubation (15 min) of (**A**) compound **1**, (**B**) compound **2**, (**C**) compound **5**, (**D**) compound **6a** and (**E**) compound **6b**.

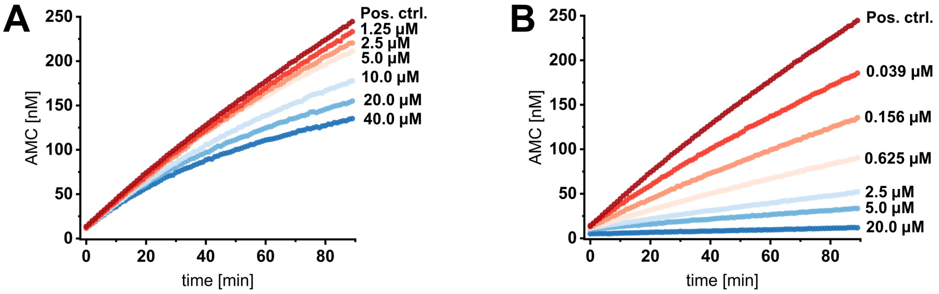

Figure S3. SIRT2-mediated progress curves of AMC formation for (**A**) **JH-T4** and (**B**) compound **2** using **ETDKMyr-AMC** as substrate.

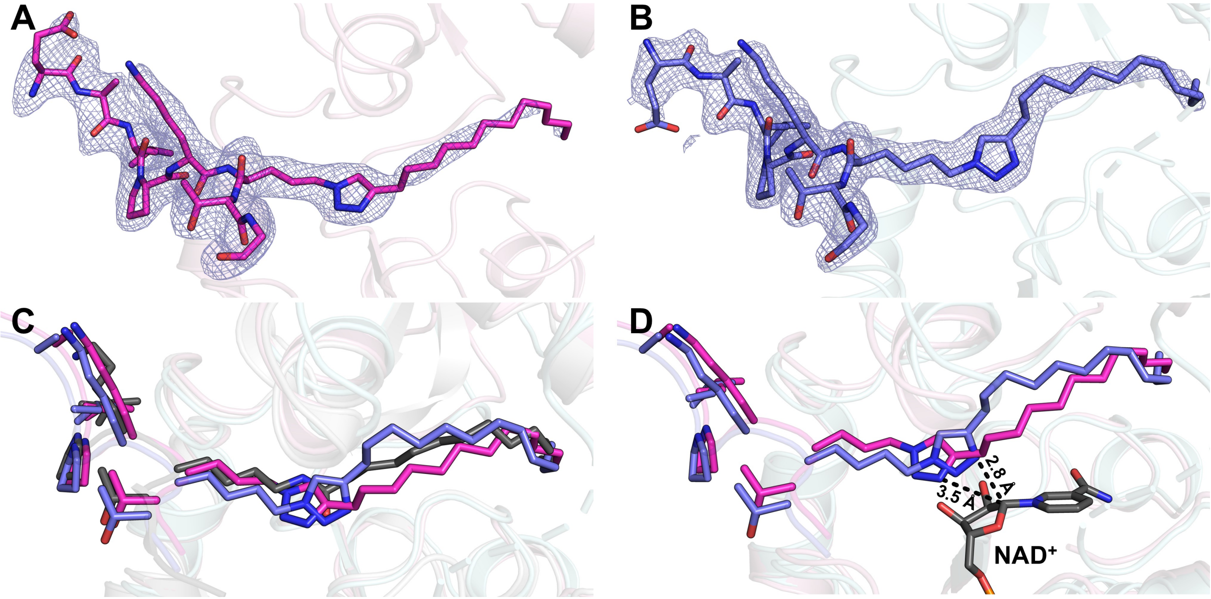

Figure S4. Crystal structures of (**A**) SIRT2–**5** at 2.30 Å resolution (PDB 9S23) and (**B**) SIRT2–**6** at 2.10 Å resolution (PDB 9S25) validate the expected binding site of **5** and **6** inside the acyl-lysine channel. The $2F_o - F_c$ maps are depicted as blue mesh and contoured at 1.0σ. (**C**) Superimposition of SIRT2–**5** (**5**: light magenta), SIRT2–**6** (**6**: slate) and SIRT2–TNFαK20myr (TNFαK20myr: dark grey, from PDB 4Y6O)[16] reveals a shared binding mode. The triazole of **6** is positioned deeper within the acyl-lysine channel than that of **5**. (D) The altered triazole orientation in **6** reduces the distance to the *C*1' of NAD+ ribose (dark grey, from PDB 8QT4) to 2.8 Å, compared to 3.5 Å for **5**, favoring a more efficient nucleophilic attack and yielding a more stable ADPR adduct, consistent with in vitro assay data.

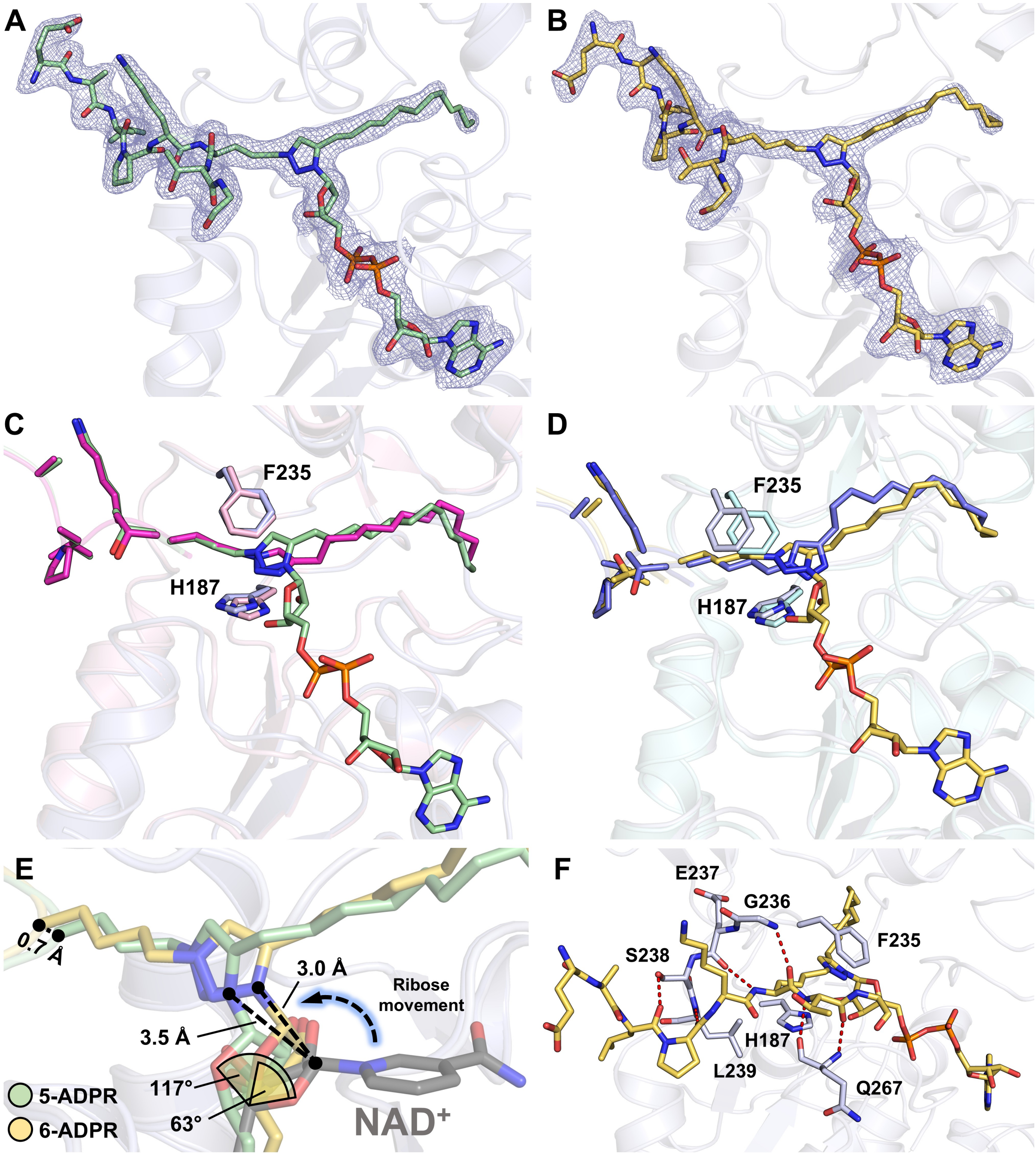

Figure S5. (**A**) Crystal structure of the SIRT2–[**5**–ADPR] complex at 2.10 Å resolution (SIRT2: lightblue, **5**–ADPR: palegreen, PDB 9S24) and (**B**) the SIRT2–[**6**–ADPR] complex at 2.30 Å resolution (SIRT2: blue-white, **6**–ADPR: yellow-orange, PDB 9S26). The $2F_o - F_c$ maps are depicted as blue mesh and contoured at 1.0σ. Both complexes display well-resolved density for the covalent adduct and the TNFα-derived peptide backbone. (**C**) Superimposition of SIRT2–[**5**–ADPR] and SIRT2–**5** (SIRT2: lightpink, **5**: light magenta) shows that π–π interactions with Phe235 are generated during adduct formation as the triazole ring tilts to accommodate covalent bonding. (**D**) Superimposition of SIRT2–[**6**–ADPR] and SIRT2–**6** (SIRT2: palecyan, **6**: slate) reveals that upon adduct formation, the triazole moiety of **6** rotates by 29° and shifts 1.0 Å outward of the channel to allow optimal alignment with ADPR positioning. A slight rotation of Phe235 in the SIRT2–[**6**–ADPR] structure indicates enhanced shielding of the electrophilic ribose-*C*1' from solvent hydrolysis. (**E**) Superimposition of the SIRT2–[**5**–ADPR] (**5**–ADPR: pale green) and SIRT2–[**6**–ADPR] (**6**–ADPR: yellow-orange) complexes illustrates structural features underpinning the 200-fold affinity difference. Compound **5** is pushed 0.7 Å deeper into the acyl-lysine channel to react with NAD^+, increasing conformational strain. Its triazole-N3 attacks the C1' of NAD^+ ribose at a less favorable 3.5 Å distance and requires a 117° ribose tilt (vs. 3.0 Å and 63° for **6**, respectively; NAD^+ from PDB 8QT4). (**F**) In the SIRT2–[**6**–ADPR] complex, additional hydrogen bonding between **6** and backbone residues Gly236, Glu237, Ser238, and Gln267 of SIRT2 stabilizes the binding conformation and enhances slow-binding behavior, contributing to higher affinity.

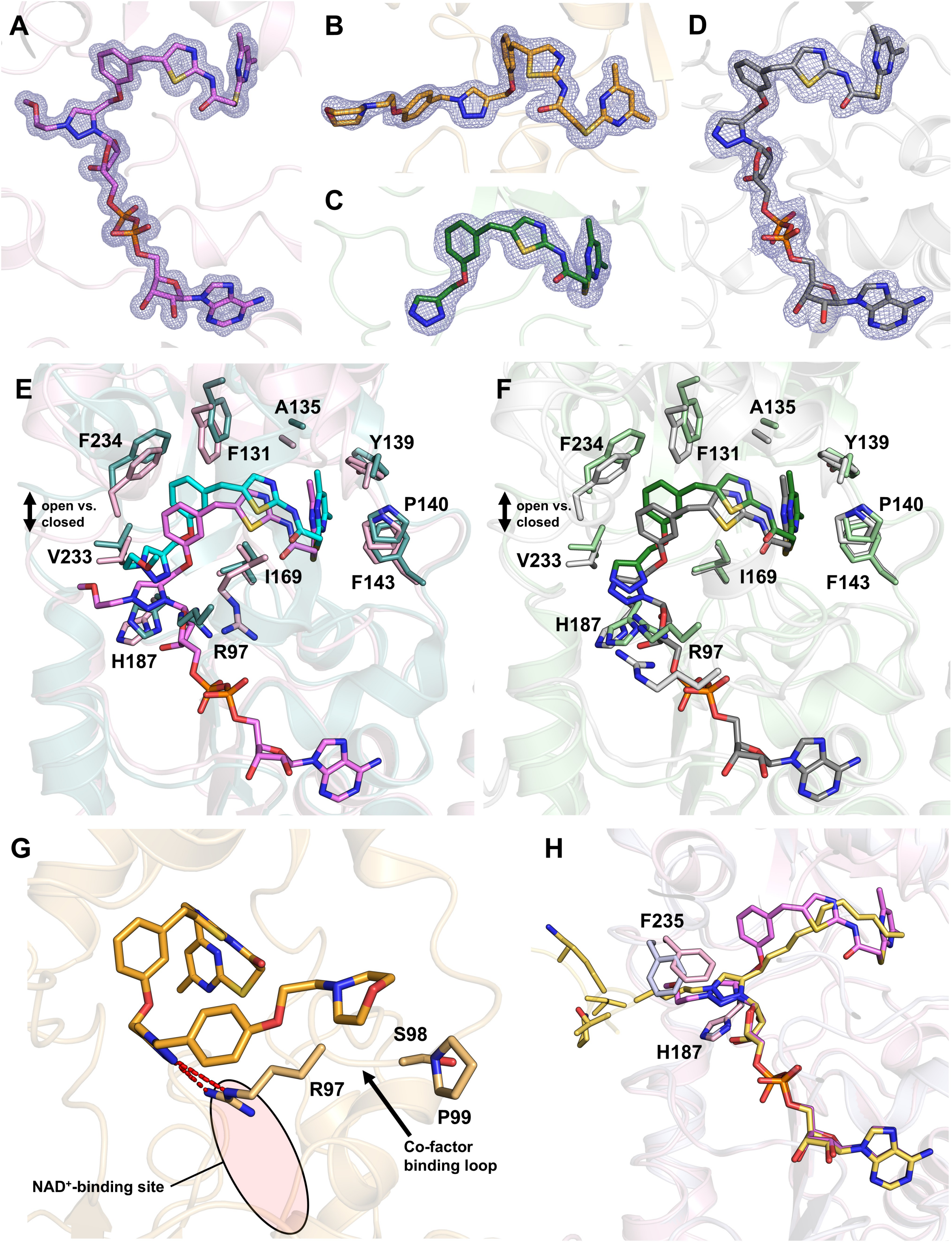

Figure S6. Crystal structures of (**A**) SIRT2–[**1**–ADPR] at 1.10 Å resolution (PDB 9S1Z), (**B**) SIRT2–**2** at 1.50 Å resolution (PDB 9S20), (**C**) SIRT2–**7** at 1.55 Å resolution (PDB 9S21) and (**D**) SIRT2–[**7**–ADPR] at 1.95 Å resolution (PDB 9S22). The $2F_o - F_c$ maps are depicted as blue mesh and contoured at 1.0σ. (**E**) Superimposition of the SIRT2–[**1**–ADPR] (SIRT2: light pink, **1**–ADPR: pink) and SIRT2–**1** (SIRT2: light teal, **1**: cyan, from PDB 8OWZ) complexes and (**F**) Superimposition of the SIRT2–[**7**–ADPR] (SIRT2: light grey, **7**–ADPR: grey) and SIRT2–**7** (SIRT2: pale green, **7**: dark green) complexes. The NAD^+ induced formation of the **1**–ADPR and **7**–ADPR adducts results in a Zn^{2+}-binding domain closure. (**G**) Although NAD^+ was present during crystallization of the SIRT2–**2** complex, it was absent in the final structure – likely due to cofactor binding loop (residues 90 – 110) stabilization by compound **2**, positioning Arg97 to obstruct NAD^+ binding. (**H**) Superposition of SIRT2–[**1**–ADPR] and SIRT2–[**6**–ADPR] structures reveals a highly conserved positioning of the triazolium-ADPR moiety, despite the distinct overall scaffolds of **1** and **6**.

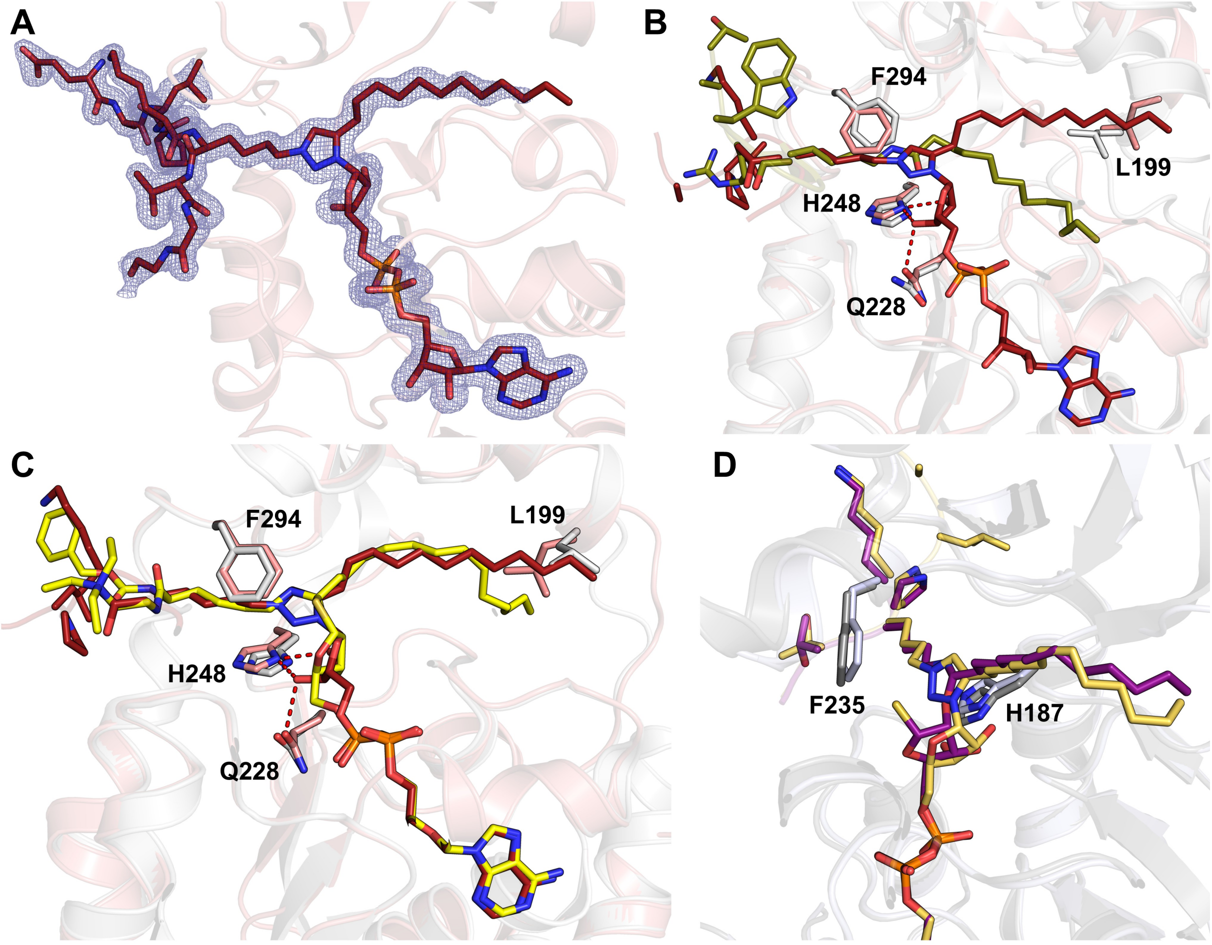

Figure S7. (**A**) Crystal structure of SIRT3–[**6**–ADPR] at 1.60 Å resolution (PDB 9S27). The $2F_o - F_c$ map (blue mesh, contoured at 1.0σ) indicates reduced density along the distal dodecyl chain of **6**–ADPR, suggesting increased flexibility due to surface exposure. (**B**) Superimposition of SIRT3–[**6**–ADPR] (SIRT3: salmon, **6**: firebrick) with SIRT3–H3K9myr (SIRT3: light grey, H3K9myr: olive, PDB 5BWN).[54] (**C**) Superimposition of SIRT3–[**6**-ADPR] with a stalled thiomyristoyl intermediate bound to SIRT3 (yellow, PDB 9CBT)[55] highlights differing alkyl chain orientations driven by Leu199 positioning. (**D**) Comparison of SIRT2–[**6**–ADPR] with a stalled thiomyristoyl intermediate bound to SIRT2 (dark purple, PDB 4X3O).[56]

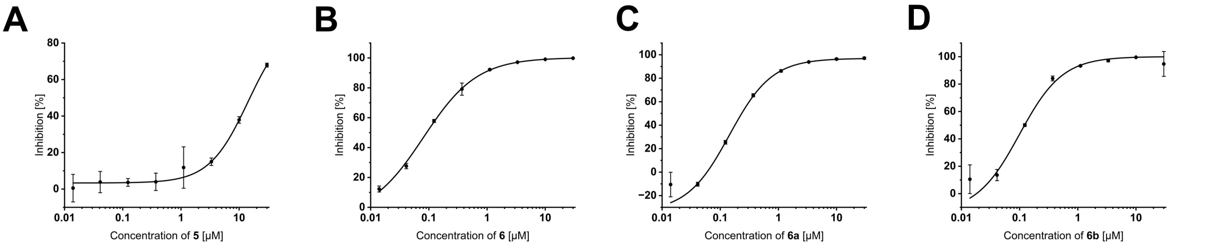

Figure S8. IC_{50} curves from SIRT1 deacetylation assays (ZMAL assay) for (**A**) compound **5**, (**B**) compound **6**, (**C**) compound **6a** and (**D**) compound **6b**.

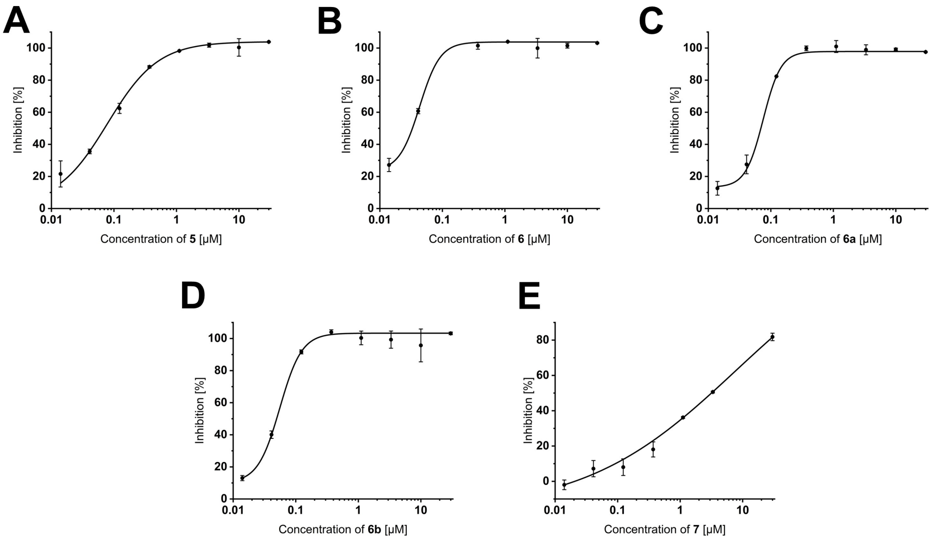

Figure S9. IC_{50} curves from SIRT2 deacetylation assays (ZMAL assay) for (**A**) compound **5**, (**B**) compound **6**, (**C**) compound **6a**, (**D**) compound **6b** and (**E**) compound **7**.

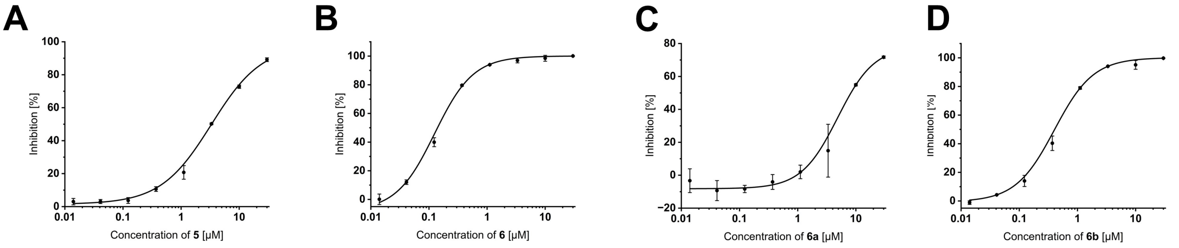

Figure S10. IC_{50} curves from SIRT3 deacetylation assays (ZMAL assay) for (**A**) compound **5**, (**B**) compound **6**, (**C**) compound **6a** and (**D**) compound **6b**.

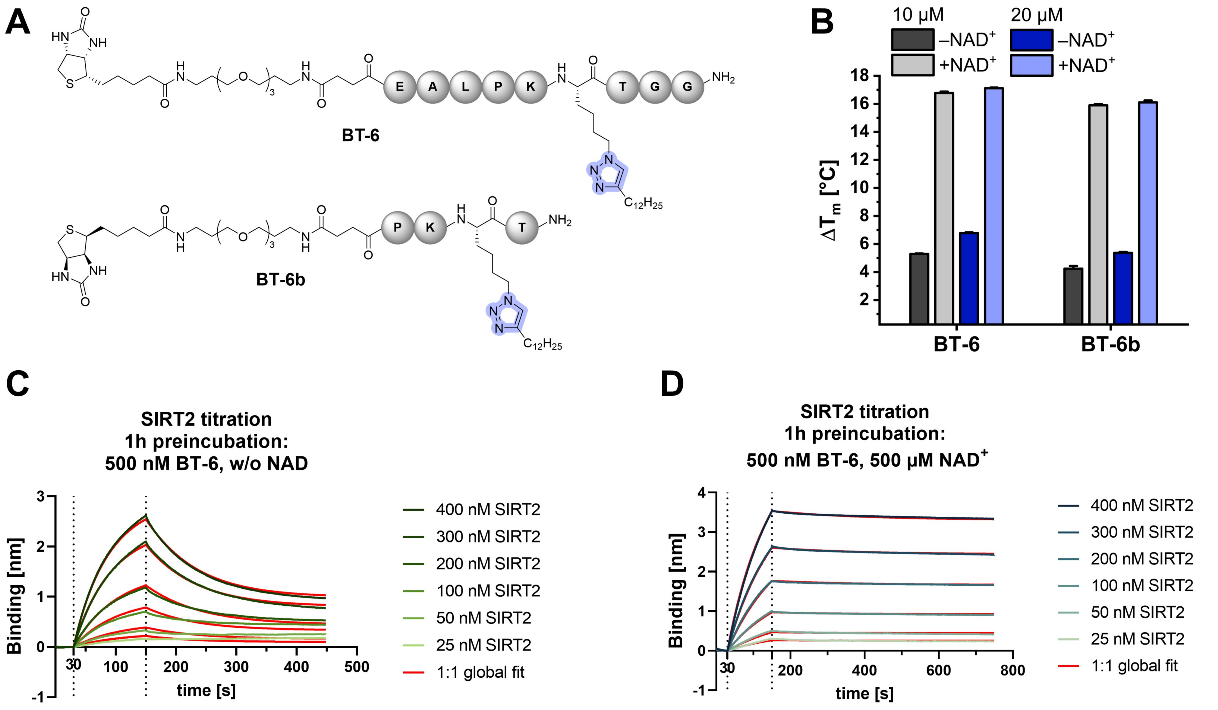

Figure S11. (**A**) Chemical structures of the biotinylated probes **BT-6** and **BT-6b**, derived from compounds **6** and **6b**, respectively. (**B**) Fluorescence thermal shift assay results showing strong SIRT2 stabilization upon NAD$^+$ addition for both **BT-6** and **BT-6b**, indicating that linker and biotin incorporation do not adversely affect binding. **(C)** BLI analysis of SIRT2 at varying concentrations with **BT-6**; global fit to a 1:1 binding model shown. **(D)** BLI analysis of SIRT2 at varying concentrations with **BT-6** and NAD$^+$; global fit to a 1:1 binding model shown.

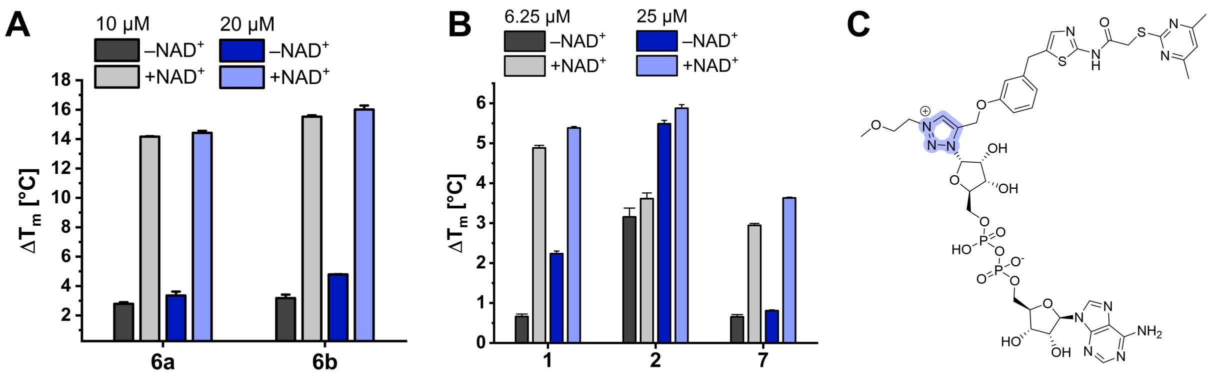

Figure S12. SIRT2 fluorescence thermal shift assay results of (**A**) LTDi-1a (**6a**) and LTDi-1b (**6b**) and (**B**) SirReal-triazoles **1**, **2** and **7**. (**C**) Chemical structure of the **1**–ADPR adduct.

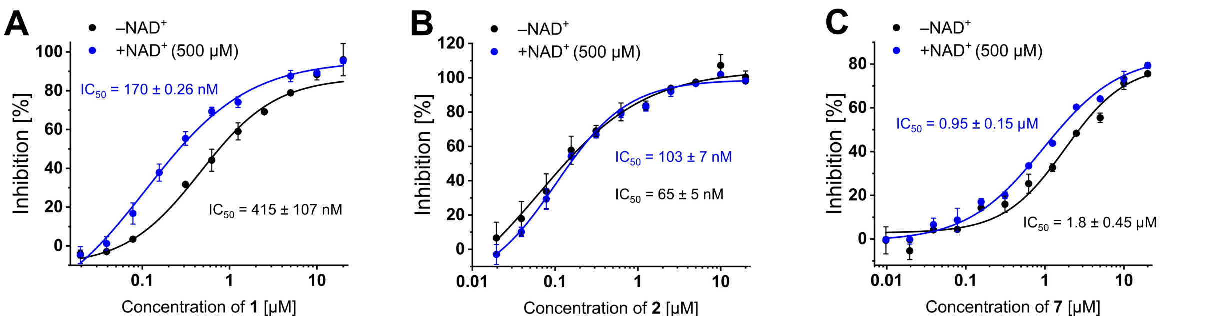

Figure S13. IC_{50} curves from SIRT2 fluorescence polarization assays for SirReal-triazoles (**A**) **1**, (**B**) **2** and (**C**) **7**. Only Compound **1** and **7** show increased affinities upon NAD$^+$ addition.

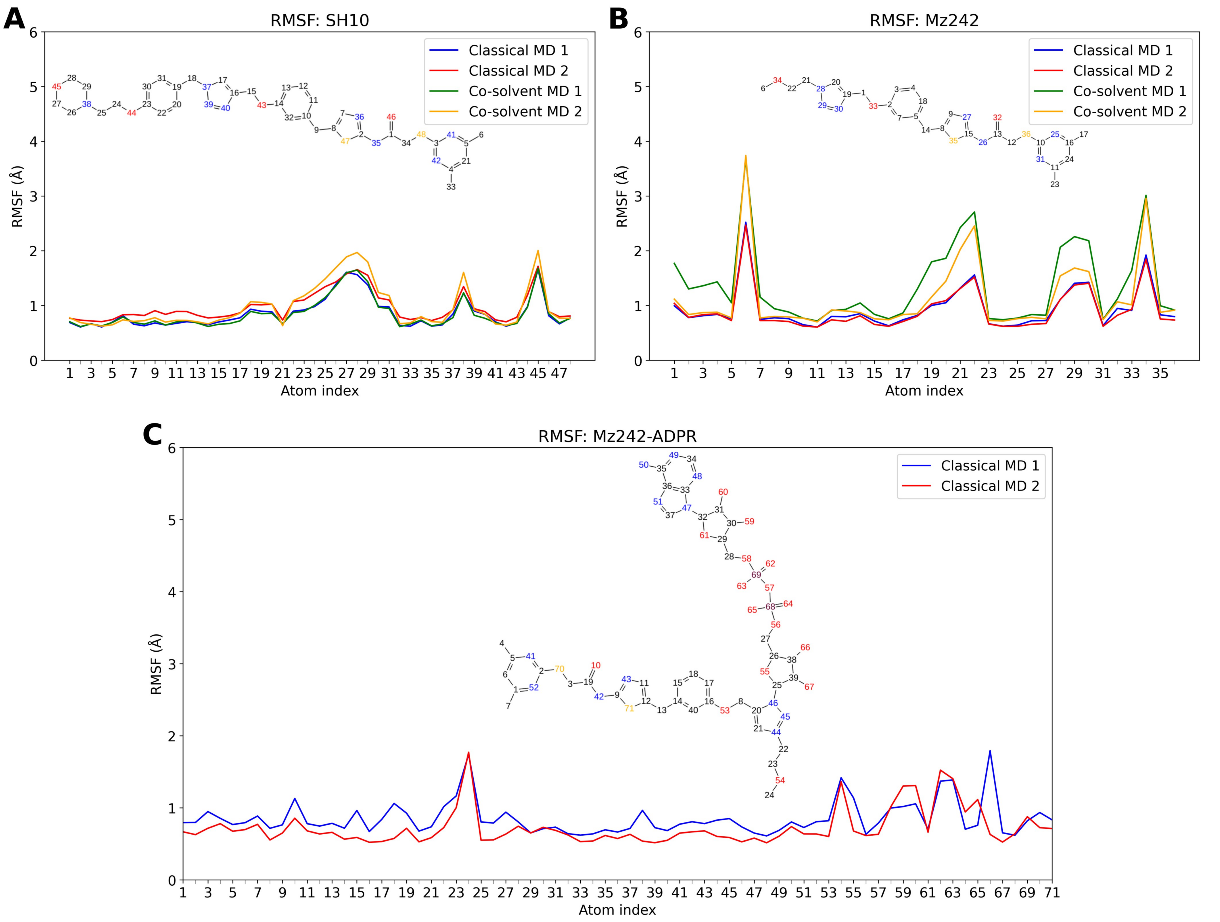

Figure S14. Ligand atom fluctuations (RMSF) calculated for MD simulations with (**A**) compound **2**, (**B**) compound **1** and (**C**) the **1**-ADPR adduct.

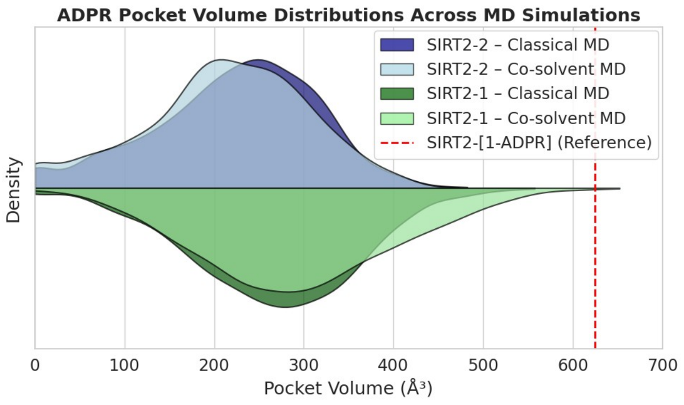

Figure S15. MD-simulations comparing the cofactor binding site pocket volume distribution for compound **1** and **2**. Co-solvent MD with xenon probes shows limited NAD$^+$ pocket opening in the **2**–SIRT2 complex (light-blue), while covalent inhibitor **1** increases pocket volume (light-green), suggesting that reduced loop stabilization facilitates NAD$^+$ binding and adduct formation.

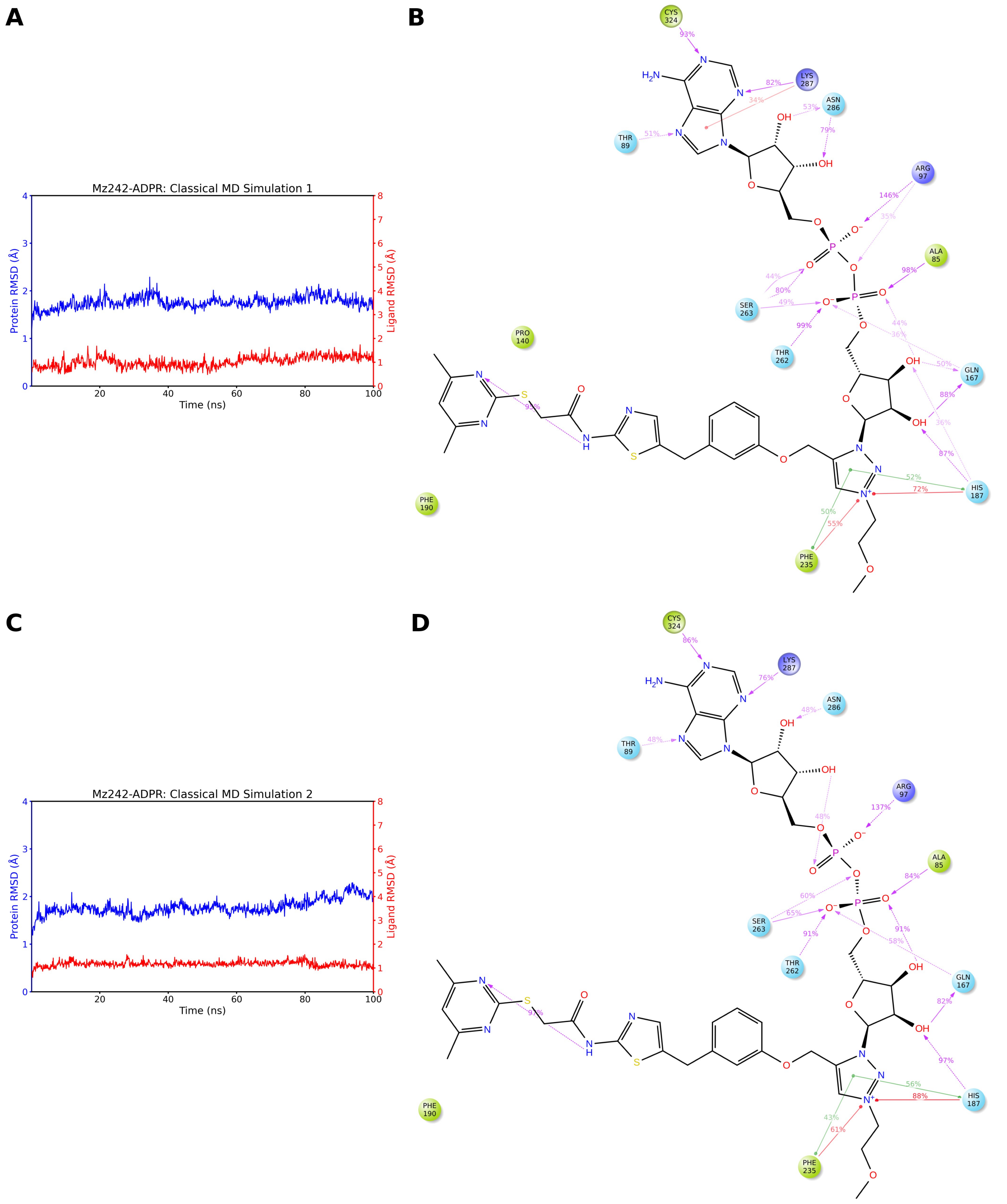

Figure S16. Protein and ligand RMSD graphs (**A**, **C**) as well as simulation interactions diagrams (**B**, **D**) obtained for all classical MD simulations of the SIRT2–[**1**–ADPR] complex.

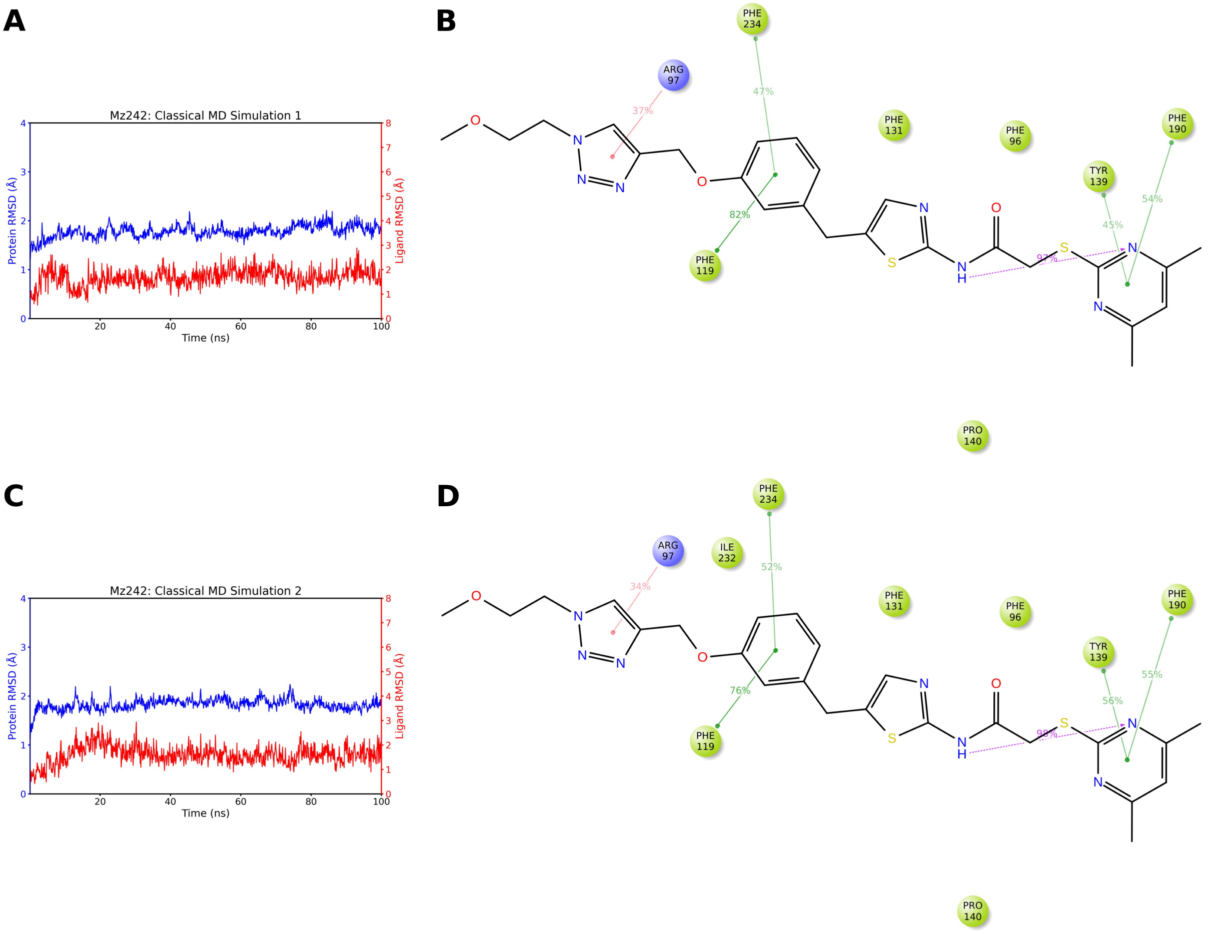

Figure S17. Protein and ligand RMSD graphs (**A**, **C**) as well as simulation interactions diagrams (**B**, **D**) obtained for all classical MD simulations of the SIRT2–**1** complex.

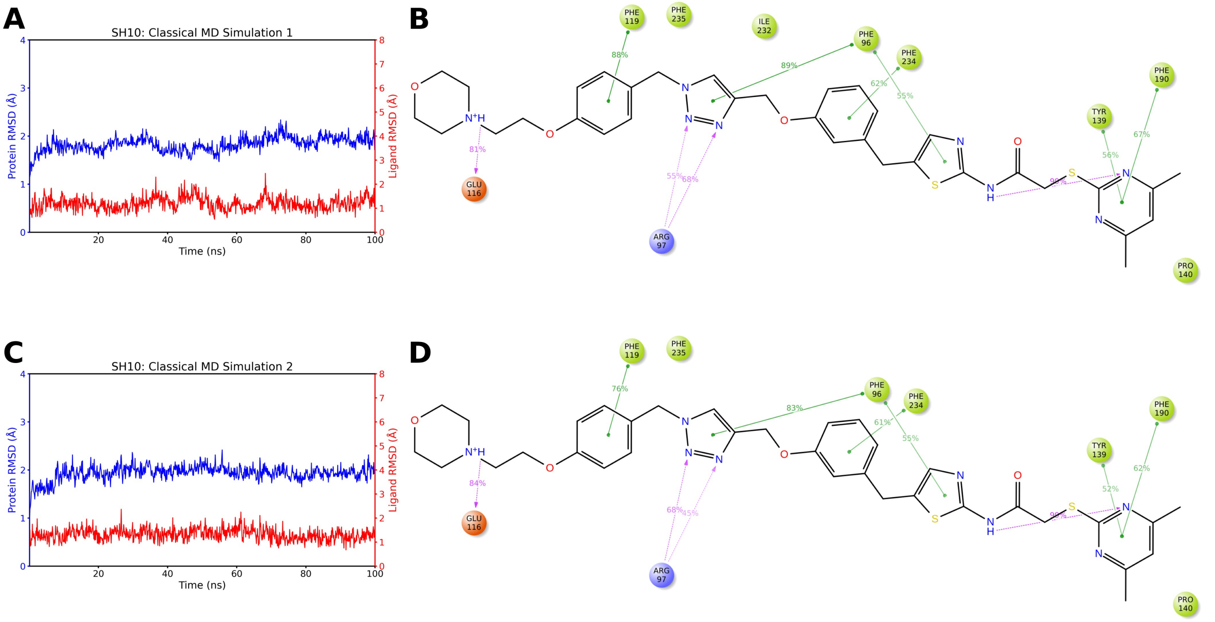

Figure S18. Protein and ligand RMSD graphs (**A**, **C**) as well as simulation interactions diagrams (**B**, **D**) obtained for all classical MD simulations of the SIRT2–**2** complex.

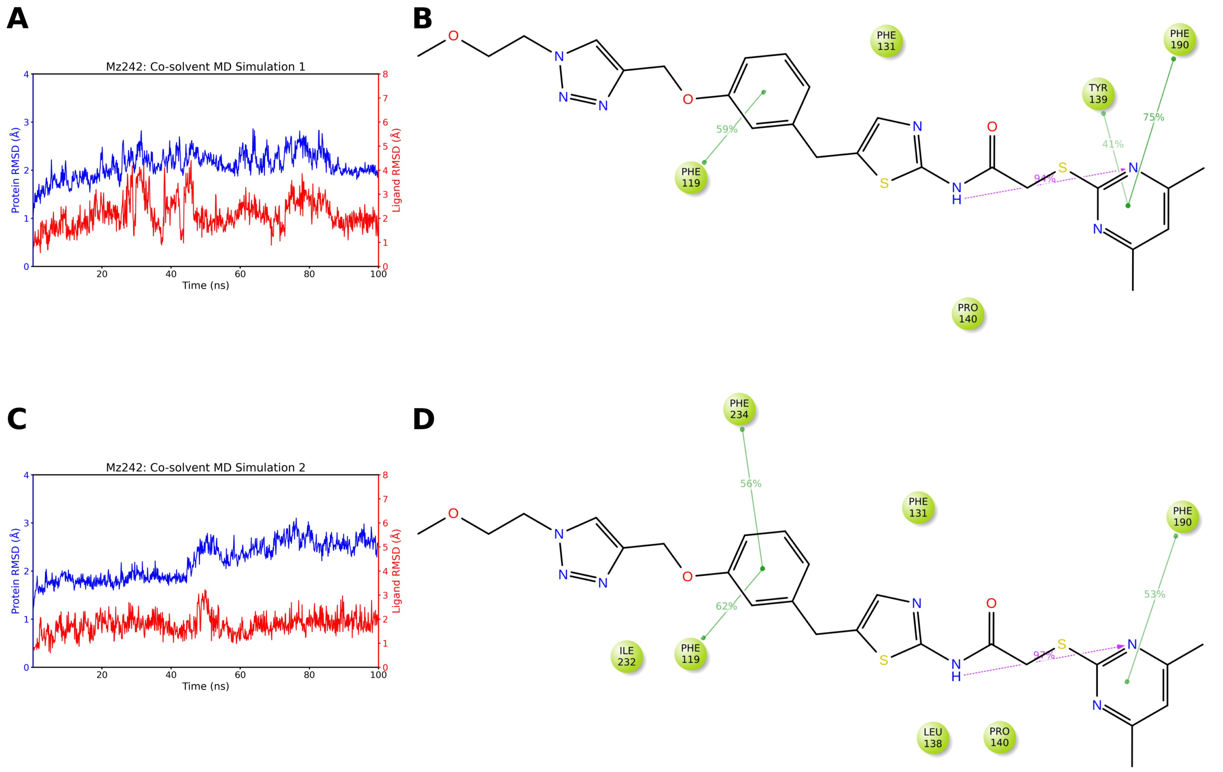

Figure S19. Protein and ligand RMSD graphs (**A**, **C**) as well as simulation interactions diagrams (**B**, **D**) obtained for all co-solvent MD simulations of the SIRT2–**1** complex.

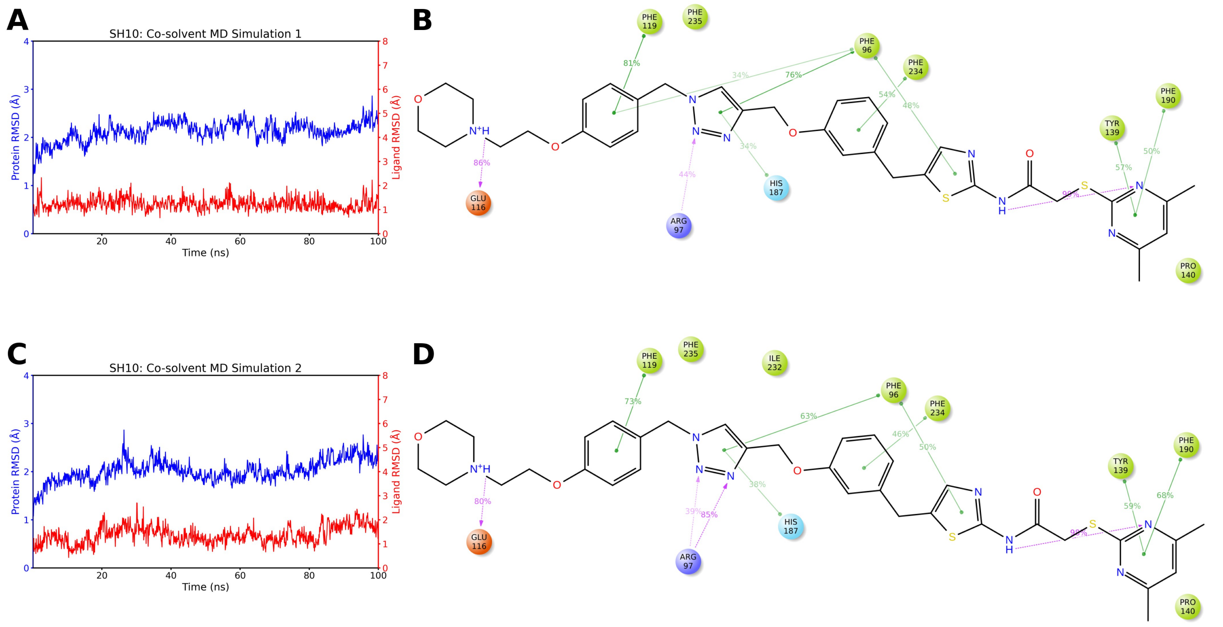

Figure S20. Protein and ligand RMSD graphs (**A**, **C**) as well as simulation interactions diagrams (**B**, **D**) obtained for all co-solvent MD simulations of the SIRT2–**2** complex.

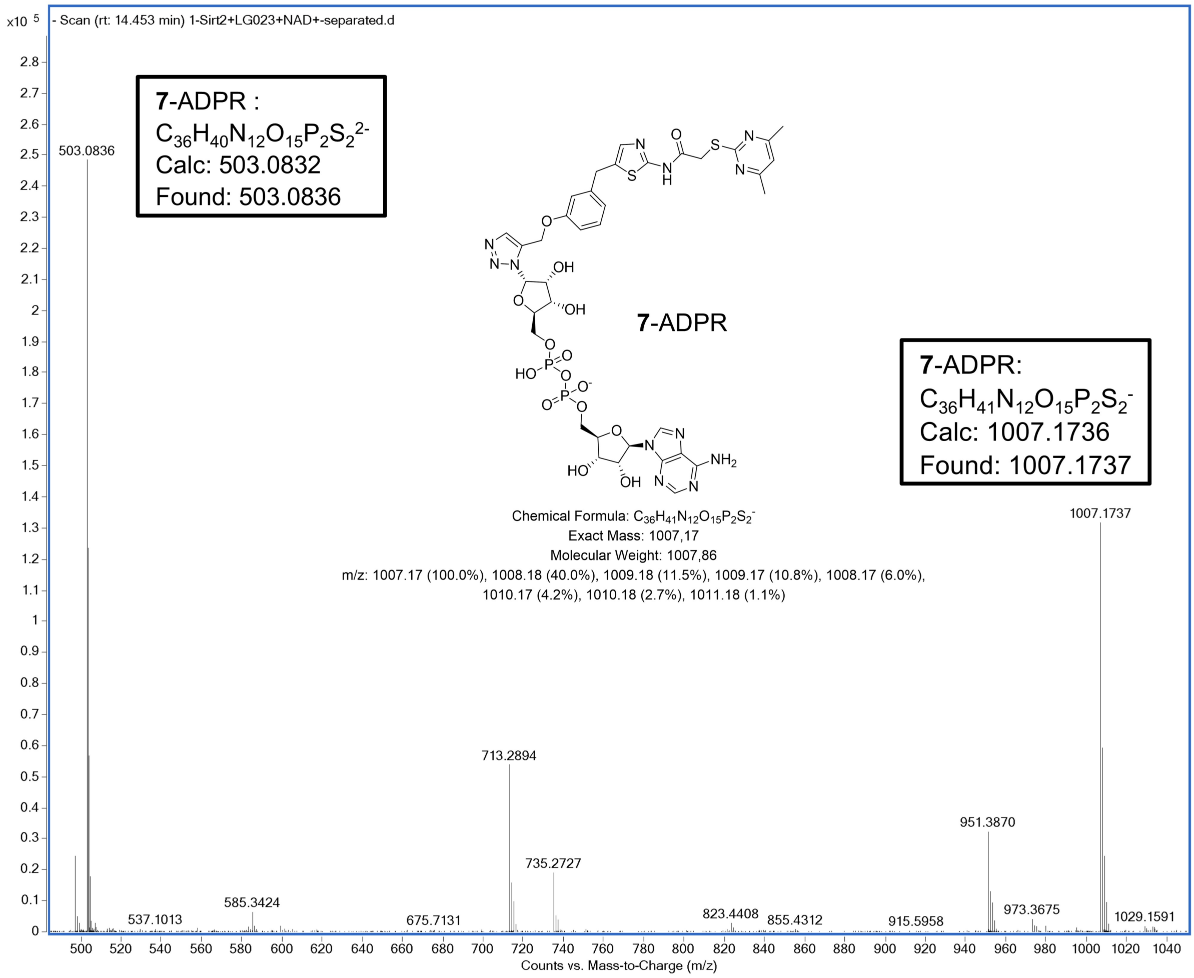

Figure S21. Original CE-ESI-MS data of the SIRT2–[**7**–ADPR] adduct analysis (m/z = 503.08 [2−], 1007.17 [1−]) and structure of proposed **7**-ADPR adduct.

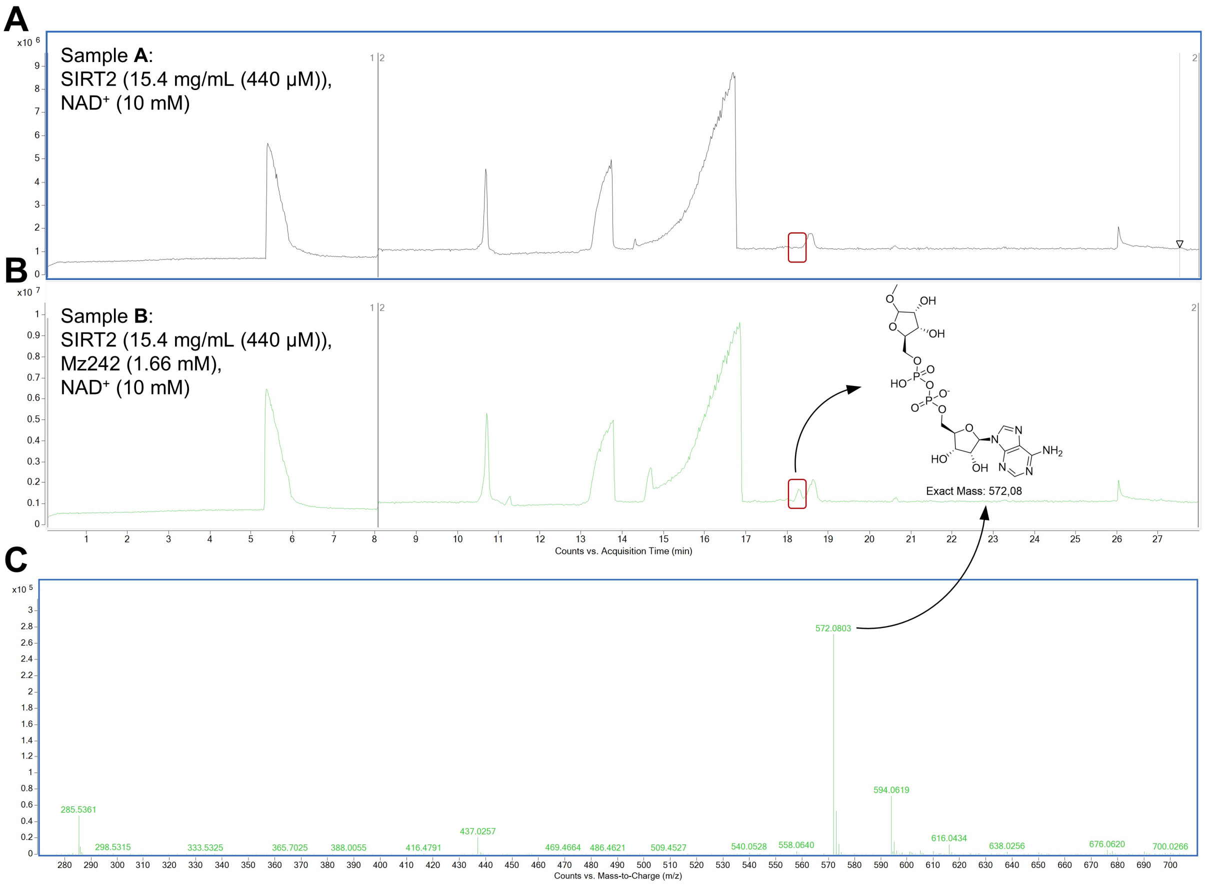

D

Sample	NAD+ (%)	ADPR (%)	MeO-ADPR (%)
**A**: SIRT2 + NAD+	89.9	9.8	0.3
**B**: SIRT2 + NAD+ + Mz242	82.1	13.1	4.8

E

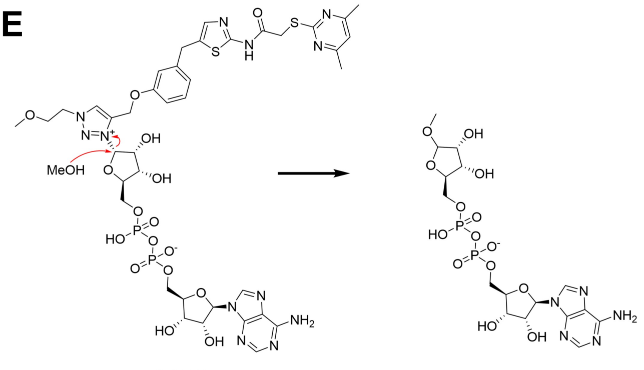

Figure S22. CE-ESI-MS results using MeOH as SIRT2 denaturing agent. (**A**) CE-ESI-MS data of the negative control containing only SIRT2 and NAD^+ (sample A). (**B**) CE-ESI-MS data of sample B containing SIRT2, NAD^+, and **1**, showing pronounced MeO–ADPR formation. (**C**) *m/z* values and experimental spectrum of MeO–ADPR. (**D**) Quantitative summary of NAD^+, ADPR, and MeO–ADPR contents determined by CE-MS for samples A and B. Assuming one trapped **1**–ADPR adduct per SIRT2 molecule, the theoretical maximum MeO–ADPR content (4.5%) agrees well with the measured value (4.8%) for sample B. (**E**) Proposed mechanism of MeO–ADPR formation in solution upon protein denaturation.

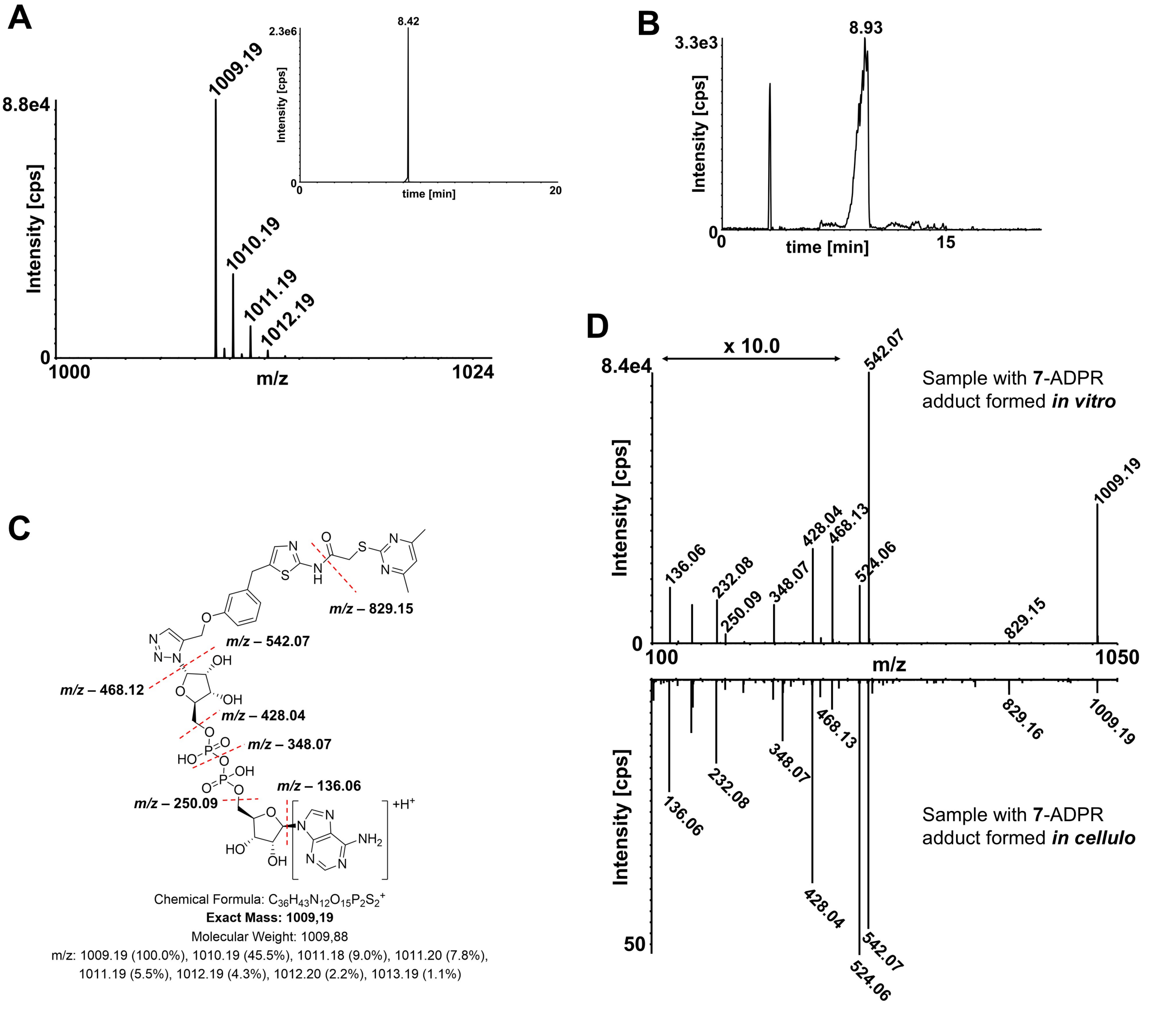

Figure S23. CE-ESI-MS data in positive mode, including in vitro reference as validation for results of **7**-ADPR adduct formed *in cellulo*. (**A**) Extracted ion electropherogram of in vitro standard and CE-ESI-MS spectrum confirming formation and solution stability of the **7**–ADPR adduct (m/z = 1009.19 $[M+H]^+$). (**B**) Extracted ion electropherogram of extracted sample from HEK293T cells. (**C**) Positively charged **7**-ADPR adduct with its proposed fragmentation sites. (**D**) Comparative MS/MS spectra of the in vitro standard (top spectrum) and the sample extracted from HEK293 cells overexpressing SIRT2 (bottom spectrum). Note that fragment ions in the standard were amplified 10-fold for better comparability. Ions at *m/z* values of 232.08 and 524.06 are generated by a neutral loss of water from fragment ions with *m/z* values of 250.09 and 542.07.

Table S1. Crystallographic data collection and refinement statistics of the SIRT2–**Inhibitor** complexes.

Structure	**SIRT2–[1–ADPR]**	**SIRT2–2**	**SIRT2–7**	**SIRT2–[7–ADPR]**
Beamline	BM07	ID30B	ID30B	ID30B
**Data Collection**				
Space group	$P2_1$	$P2_1$	$P2_1$	$C2$
a, b, c (Å)	34.83, 72.74, 54.89	34.92, 77.17, 55.82	35.95, 73.91, 55.49	102.61, 47.46, 74.79
α, β, γ (deg)	90.00, 91.31, 90.00	90.00, 93.78, 90.00	90.00, 95.06, 90.00	90.00, 108.00, 90.00
Wavelength (Å)	0.9795	0.8731	0.8731	0.8731
Resolution (Å)	54.88 – 1.10 (1.12 – 1.10)	77.17 – 1.50 (1.53 – 1.50)	55.28 – 1.55 (1.58 – 1.55)	71.14 – 1.95 (2.00 – 1.95)
Total/unique no. of reflections	725,487 (108,746)	321,986 (47,249)	249,168 (38,648)	77,177 (23,415)
$R_{merge}^{a,b}$	0.061 (1.226)	0.065 (0.969)	0.069 (0.957)	0.083 (1.161)
$R_{pim}^{a,c}$	0.025 (0.508)	0.027 (0.397)	0.029 (0.421)	0.054 (0.733)
$CC_{1/2}^{a,d}$	0.999 (0.716)	0.999 (0.780)	0.999 (0.675)	0.997 (0.511)
$I/\sigma(I)^{a}$	14.0 (1.6)	13.8 (1.9)	14.5 (2.1)	8.5 (1.1)
Redundancy[a]	6.7 (6.6)	6.8 (6.9)	6.4 (6.1)	3.3 (3.4)
Completeness (%)[a]	98.3 (97.4)	99.9 (100)	92.1 (100)	93.0 (99.7)
**Refinement**				
No. of reflections used in refinement/test set	108,693 (10,764)	47,201 (4,713)	38,619 (4,169)	23,390 (2,491)
R_{work}^{e}	0.165 (0.278)	0.160 (0.237)	0.172 (0.244)	0.178 (0.328)
R_{free}^{f}	0.177 (0.301)	0.182 (0.247)	0.204 (0.289)	0.214 (0.367)
Number of Atoms[g]	2827	2695	2585	2597
Average *B*-Factors (Å^2)	18.0	25.7	25.5	40.1
protein	16.7	24.5	25.0	40.2
ligands	14.3	30.1	29.0	30.9
solvent	27.1	35.1	30.4	42.1
RMS Deviations				
bonds (Å)	0.008	0.006	0.006	0.009
angles (deg)	0.96	0.88	0.84	0.97
Ramachandran plot (%)[h]				
favored	98.28	97.92	98.25	97.32
allowed	1.72	2.08	1.75	2.68
outliers	0	0	0	0
PDB accession code	**9S1Z**	**9S20**	**9S21**	**9S22**

[a] Values in parentheses refer to the highest-resolution shell of the data.
[b] $R_{merge} = \sum|I_h - \langle I_h\rangle|/\sum\langle I_h\rangle$; I_h = intensity measure for reflection *h;* $\langle I_h\rangle$ = average intensity for reflection *h* calculated from replicate data.
[c] $R_{pim} = \sum(1/(n-1))^{1/2}|I_h - \langle I_h\rangle|/\sum\langle I_h\rangle$; *n* = number of observations (redundancy).
[d] $CC_{1/2} = \sigma_T^2/(\sigma_T^2 + \sigma_\varepsilon^2)$, where σ_T^2 is the true measurement error variance and σ_ε^2 is the independent measurement error variance.
[e] $R_{work} = \sum||F_o| - |F_c||/\sum|F_o|$ for reflections contained in the working set. $|F_o|$ and $|F_c|$ are the observed and calculated structure factor amplitudes, respectively.
[f] $R_{free} = \sum||F_o| - |F_c||/\sum|F_o|$ for reflections contained in the test set held aside during refinement.
[g] Per asymmetric unit.
[h] Assessed by MolProbity.

Table S2. Crystallographic data collection and refinement statistics of the SIRT2–**Inhibitor** complexes.

Structure	**SIRT2–5**	**SIRT2–6**	**SIRT2–[5–ADPR]**	**SIRT2–[6–ADPR]**
Beamline	ID23-2	ID23-2	ID30B	ID23-2
**Data Collection**				
Space group	$P4_3\,2_1\,2$	$P4_3\,2_1\,2$	$P4_3\,2_1\,2$	$P4_3\,2_1\,2$
a, b, c (Å)	89.62, 89.62, 133.03	83.52, 83.52, 132.94	88.92, 88.92, 133.28	89.68, 89.68, 133.90
α, β, γ (deg)	90.00, 90.00, 90.00	90.00, 90.00, 90.00	90.00, 90.00, 90.00	90.00, 90.00, 90.00
Wavelength (Å)	0.8731	0.8731	0.8731	0.8731
Resolution (Å)	74.33 – 2.30 (2.38 – 2.30)	132.94 – 2.10 (2.16 – 2.10)	88.92 – 2.10 (2.16 – 2.10)	74.51 – 2.30 (2.38 – 2.30)
Total/unique no. of reflections	658,807 (24,808)	537,668 (28,243)	786,456 (31,977)	662,338 (25,013)
$R_{merge}{}^{a,b}$	0.352 (3.592)	0.274 (2.290)	0.234 (1.717)	0.551 (3.799)
$R_{pim}{}^{a,c}$	0.069 (0.693)	0.064 (0.523)	0.048 (0.365)	0.108 (0.733)
$CC_{1/2}{}^{a,d}$	0.998 (0.335)	0.998 (0.525)	0.998 (0.310)	0.995 (0.220)
$I/\sigma(I)^{a}$	12.5 (1.3)	12.2 (2.0)	12.7 (2.6)	10.1 (1.4)
Redundancy[a]	26.6 (27.2)	19.0 (19.7)	24.6 (22.6)	26.5 (27.1)
Completeness (%)[a]	100 (100)	100 (100)	100 (100)	100 (100)
**Refinement**				
No. of reflections used in refinement/test set	24,723 (2,400)	28,159 (2,743)	31,892 (3,123)	24,934 (2,420)
$R_{work}{}^{e}$	0.226 (0.351)	0.197 (0.282)	0.213 (0.379)	0.216 (0.343)
$R_{free}{}^{f}$	0.259 (0.370)	0.225 (0.297)	0.229 (0.401)	0.252 (0.344)
Number of Atoms[g]	2325	2422	2504	2452
Average *B*-Factors (Å^2)	57.5	43.0	48.5	54.0
protein	57.6	42.9	48.8	54.4
ligands	62.5	50.3	40.5	44.6
solvent	49.8	44.0	44.7	44.8
RMS Deviations				
bonds (Å)	0.002	0.002	0.003	0.005
angles (deg)	0.55	0.55	0.62	0.76
Ramachandran plot (%)[h]				
favored	98.53	97.85	97.22	96.15
allowed	1.47	2.15	2.78	3.85
outliers	0	0	0	0
PDB accession code	**9S23**	**9S25**	**9S24**	**9S26**

[a] Values in parentheses refer to the highest-resolution shell of the data.
[b] $R_{merge} = \sum|I_h - \langle I_h\rangle|/\sum\langle I_h\rangle$; I_h = intensity measure for reflection *h;* $\langle I_h\rangle$ = average intensity for reflection *h* calculated from replicate data.
[c] $R_{pim} = \sum(1/(n-1))^{1/2}|I_h - \langle I_h\rangle|/\sum\langle I_h\rangle$; *n* = number of observations (redundancy).
[d] $CC_{1/2} = \sigma_\tau^2/(\sigma_\tau^2 + \sigma_\varepsilon^2)$, where σ_τ^2 is the true measurement error variance and σ_ε^2 is the independent measurement error variance.
[e] $R_{work} = \sum||F_o| - |F_c||/\sum|F_o|$ for reflections contained in the working set. $|F_o|$ and $|F_c|$ are the observed and calculated structure factor amplitudes, respectively.
[f] $R_{free} = \sum||F_o| - |F_c||/\sum|F_o|$ for reflections contained in the test set held aside during refinement.
[g] Per asymmetric unit.
[h] Assessed by MolProbity.

Table S3. Crystallographic data collection and refinement statistics of the SIRT3–**Inhibitor** complex.

Structure	**SIRT3–[6–ADPR]**
Beamline	ID23-2
**Data Collection**	
Space group	*H*3 2
a, b, c (Å)	97.10, 97.10, 203.67
α, β, γ (deg)	90.00, 90.00, 120.00
Wavelength (Å)	0.8731
Resolution (Å)	77.73 – 1.60 (1.63 – 1.60)
Total/unique no. of reflections	876,921 (48,997)
$R_{merge}^{a,b}$	0.170 (3.074)
$R_{pim}^{a,c}$	0.041 (0.750)
$CC_{1/2}^{a,d}$	0.999 (0.295)
$I/\sigma(I)^{a}$	14.5 (1.3)
Redundancy[a]	17.9 (17.5)
Completeness (%)[a]	100 (100)
**Refinement**	
No. of reflections used in refinement/test set	48,988 (4,811)
R_{work}^{e}	0.181 (0.324)
R_{free}^{f}	0.194 (0.347)
Number of Atoms[g]	2513
Average *B*-Factors (Å^2)	28.9
protein	28.1
ligands	26.3
solvent	37.7
RMS Deviations	
bonds (Å)	0.007
angles (deg)	0.91
Ramachandran plot (%)[h]	
favored	98.53
allowed	1.47
outliers	0
PDB accession code	**9S27**

[a] Values in parentheses refer to the highest-resolution shell of the data.
[b] $R_{merge} = \sum|I_h - \langle I_h\rangle|/\sum\langle I_h\rangle$; I_h = intensity measure for reflection *h;* $\langle I_h\rangle$ = average intensity for reflection *h* calculated from replicate data.
[c] $R_{pim} = \sum(1/(n-1))^{1/2}|I_h - \langle I_h\rangle|/\sum\langle I_h\rangle$; *n* = number of observations (redundancy).
[d] $CC_{1/2} = \sigma_\tau^2/(\sigma_\tau^2 + \sigma_\varepsilon^2)$, where σ_τ^2 is the true measurement error variance and σ_ε^2 is the independent measurement error variance.
[e] $R_{work} = \sum||F_o| - |F_c||/\sum|F_o|$ for reflections contained in the working set. $|F_o|$ and $|F_c|$ are the observed and calculated structure factor amplitudes, respectively.
[f] $R_{free} = \sum||F_o| - |F_c||/\sum|F_o|$ for reflections contained in the test set held aside during refinement.
[g] Per asymmetric unit.
[h] Assessed by MolProbity.

Supplementary NMR Spectra

NMR spectra of **Mz242** (**1**)[1] and **SH10** (**2**)[2] have been previously published.

NMR spectrum of **LG023** (**7**):

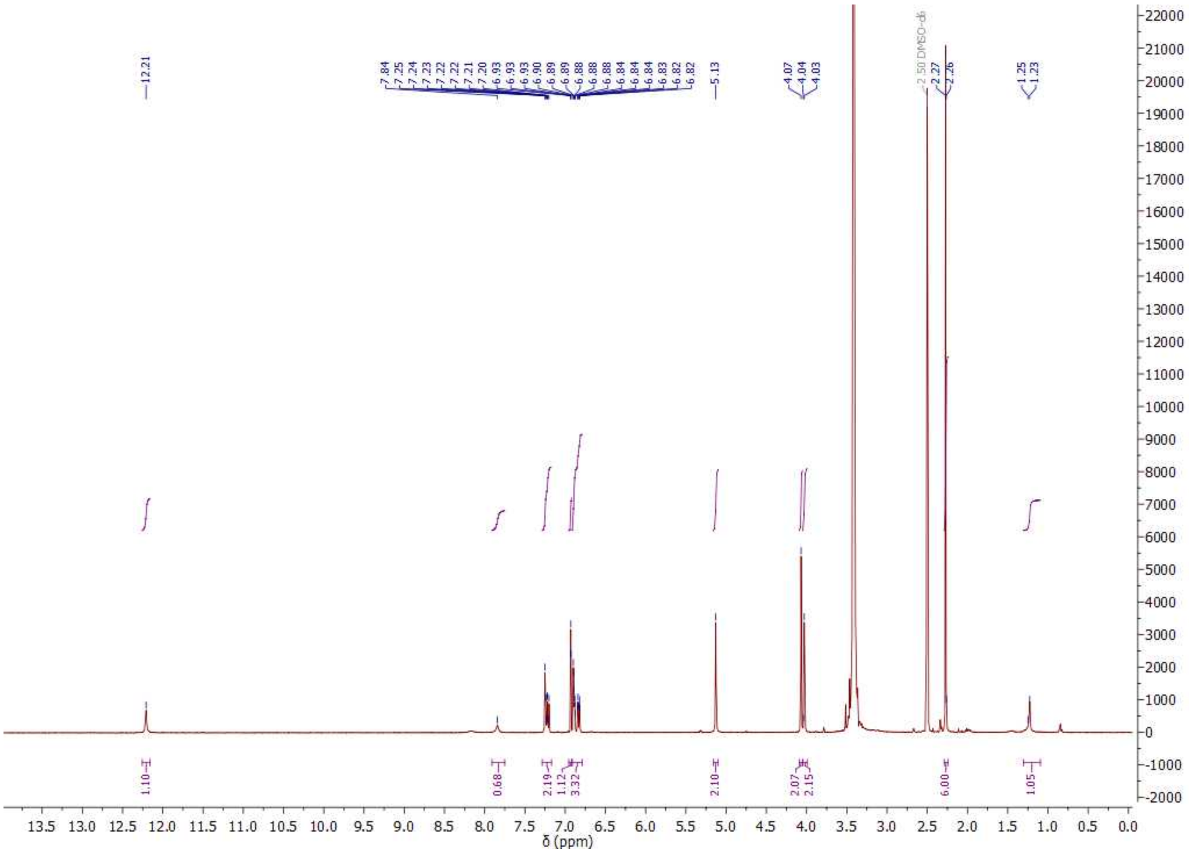

Supplementary HPLC Spectra

HPLC-MS Analysis of peptide-based triazole inhibitors

HPLC-MS analysis of compound **OTi-1** (**3**):

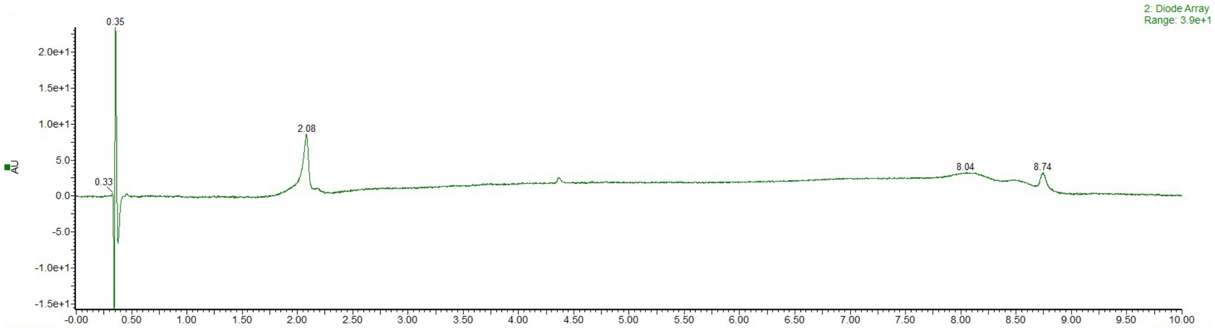

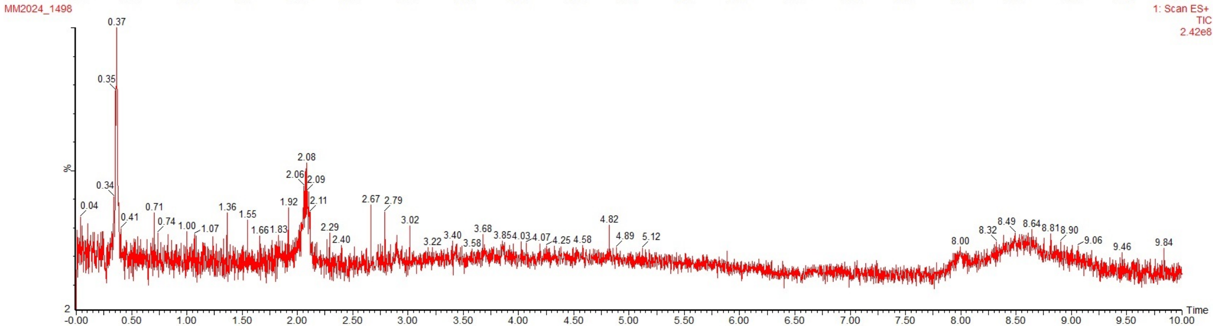

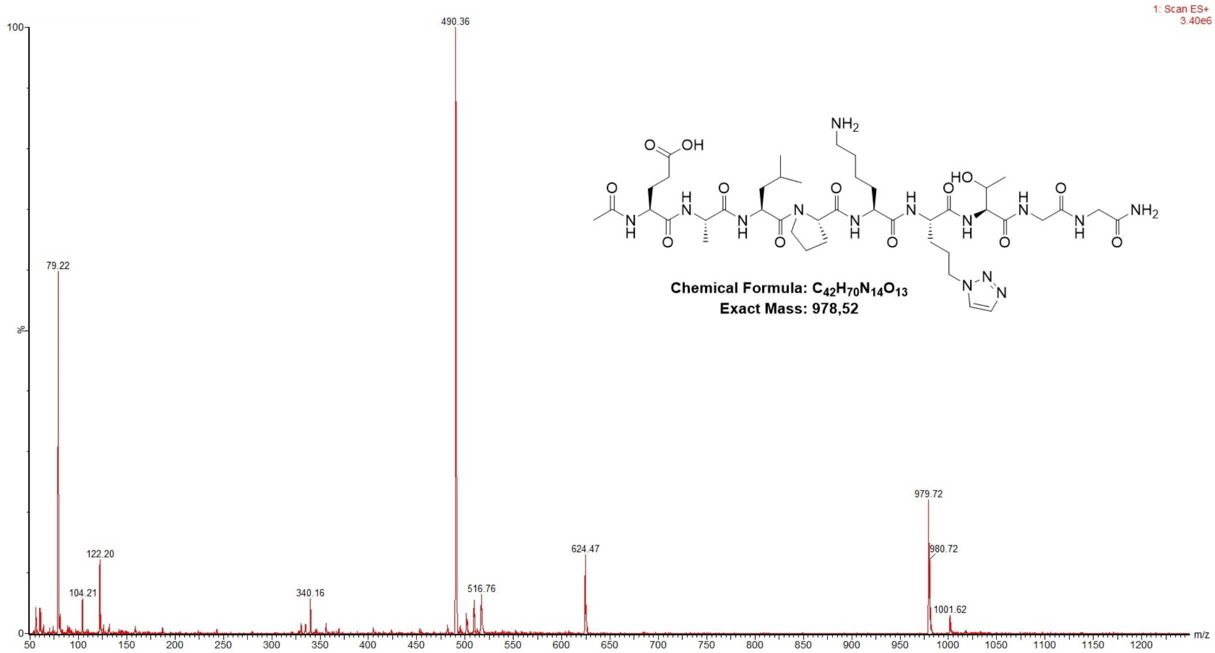

HPLC-MS analysis of compound **LTi-1** (**4**):

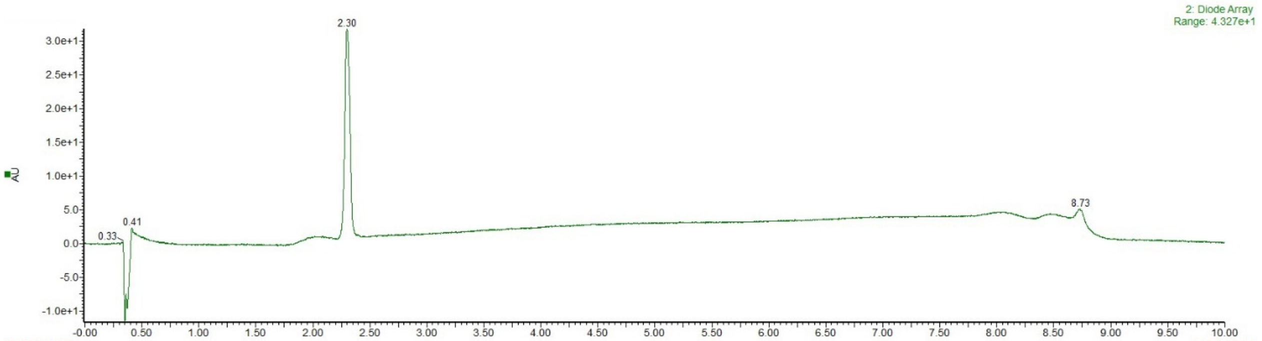

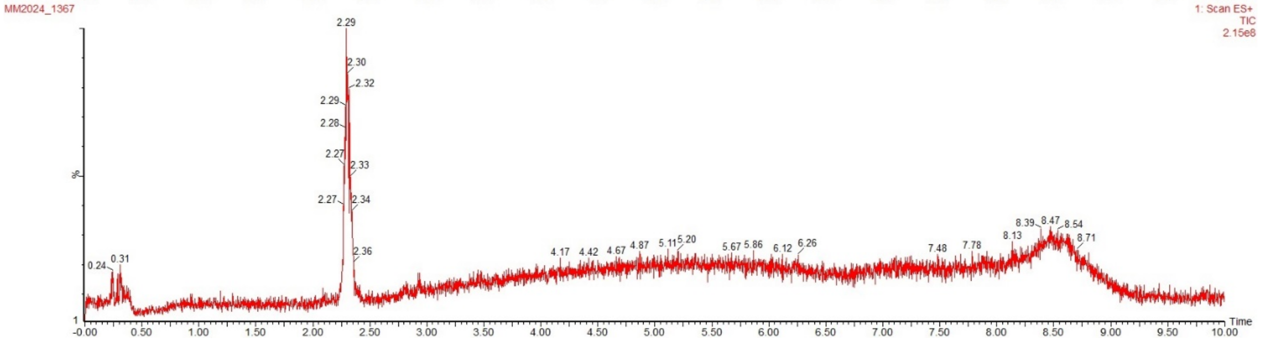

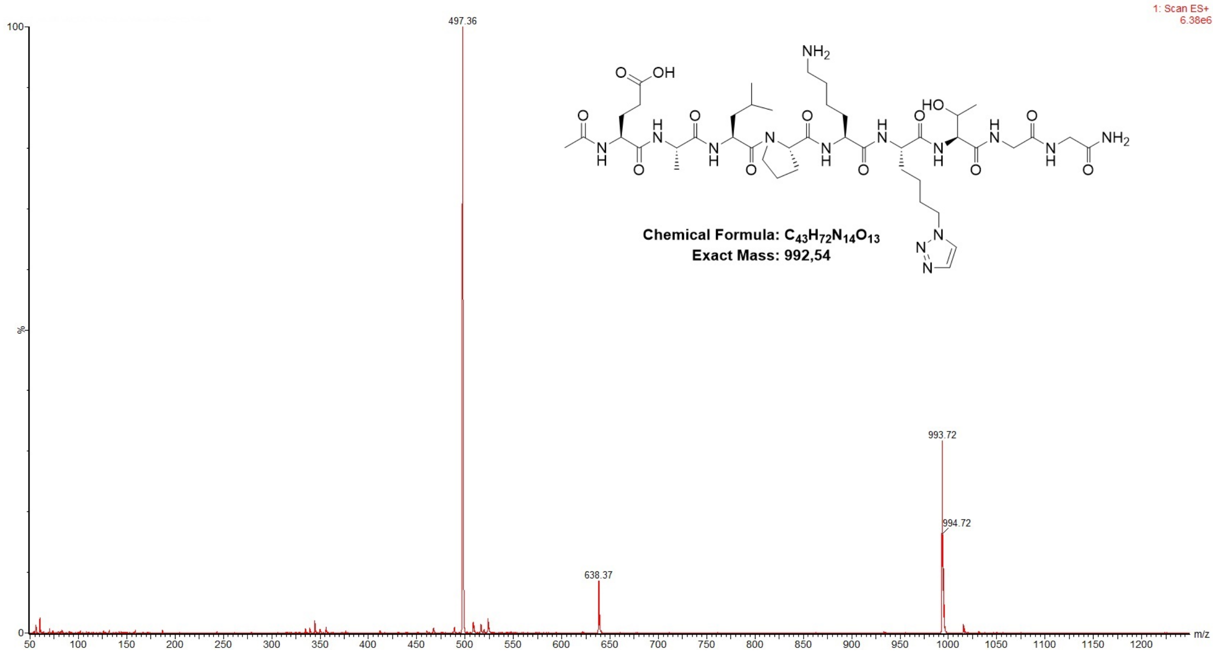

HPLC-MS analysis of compound **OTDi-1** (**5**):

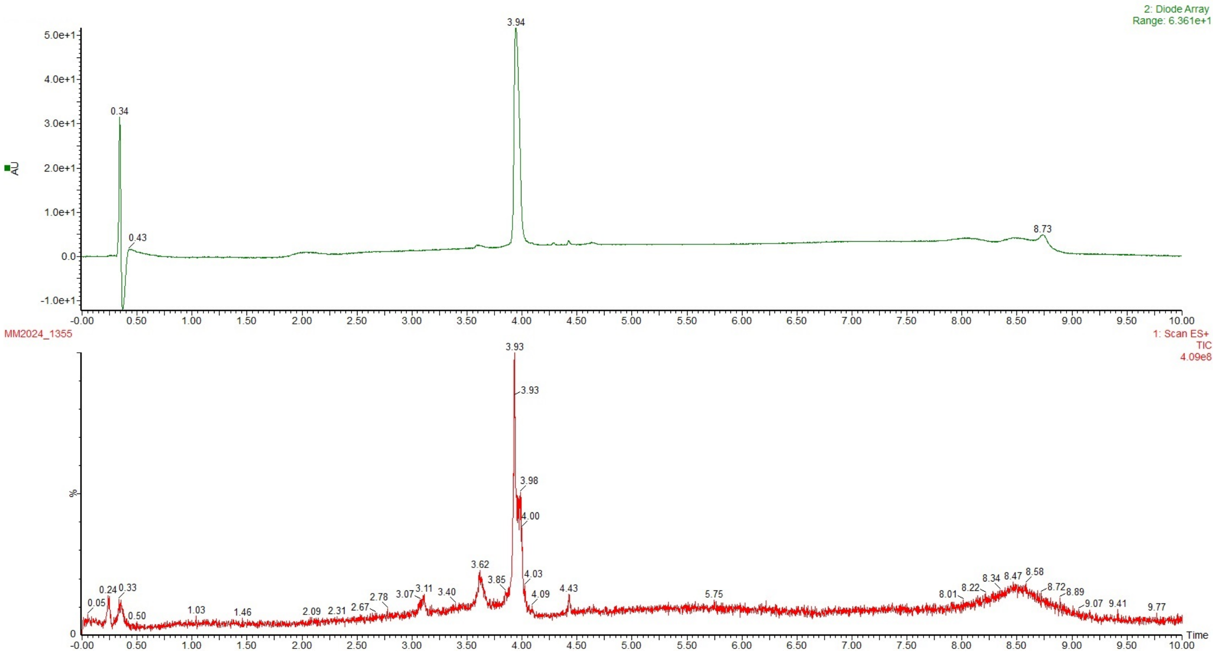

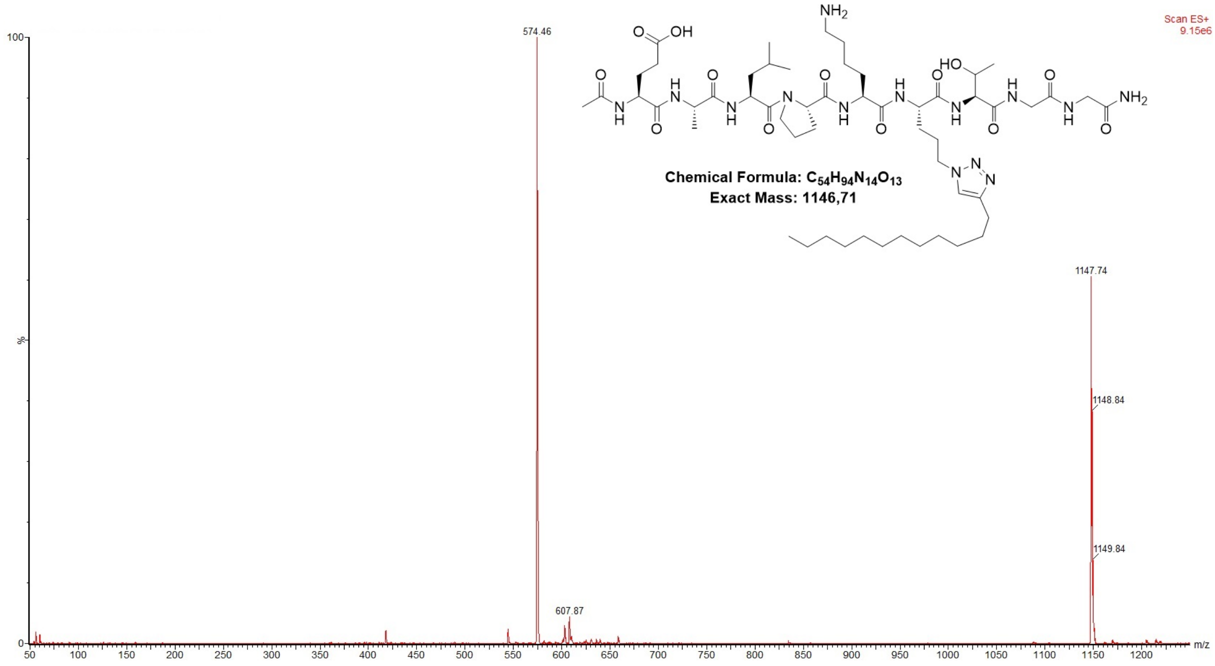

HPLC-MS analysis of compound **LTDi-1** (**6**):

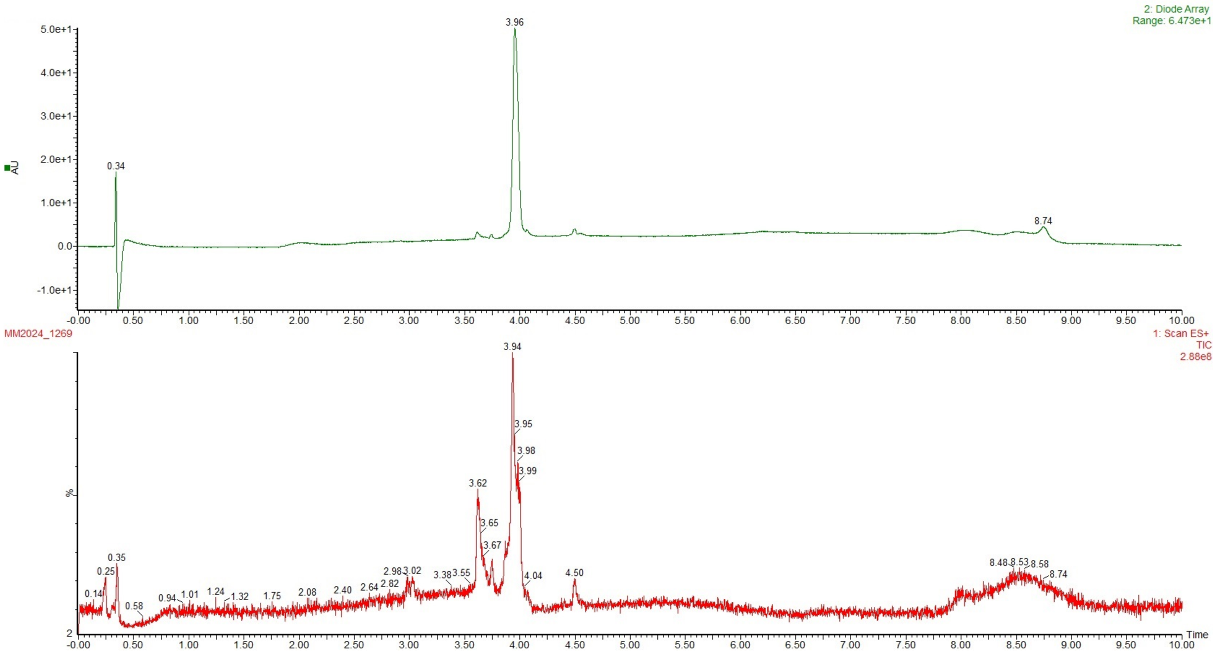

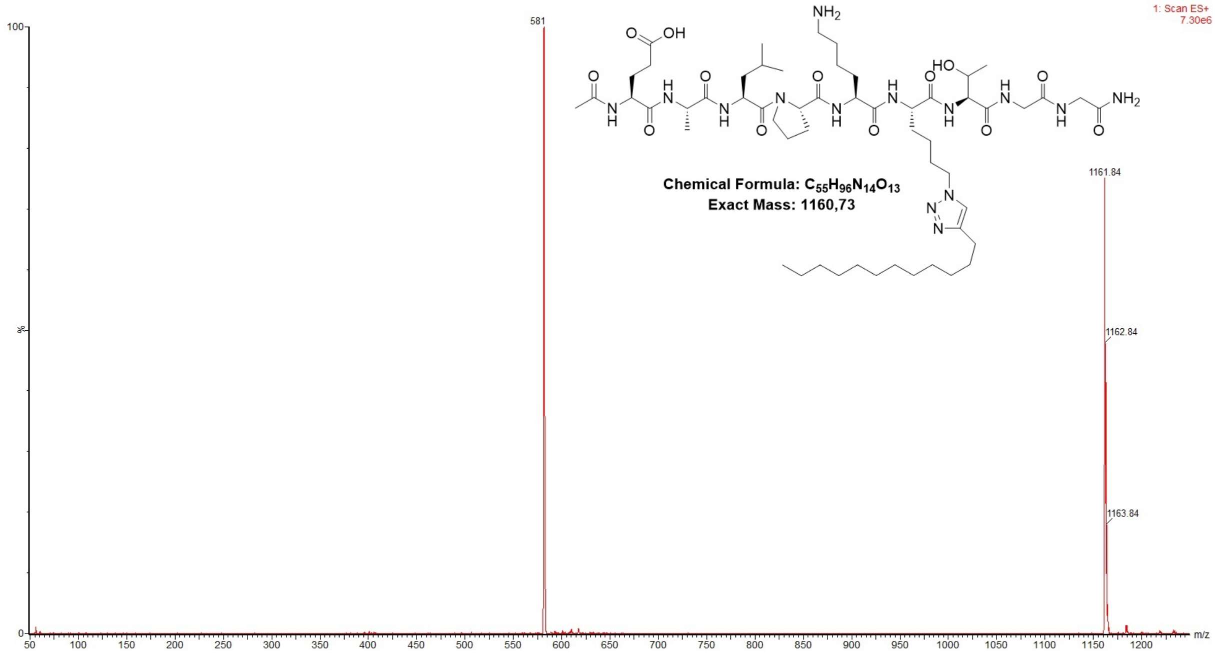

HPLC-MS analysis of compound **LTDi-1a** (**6a**):

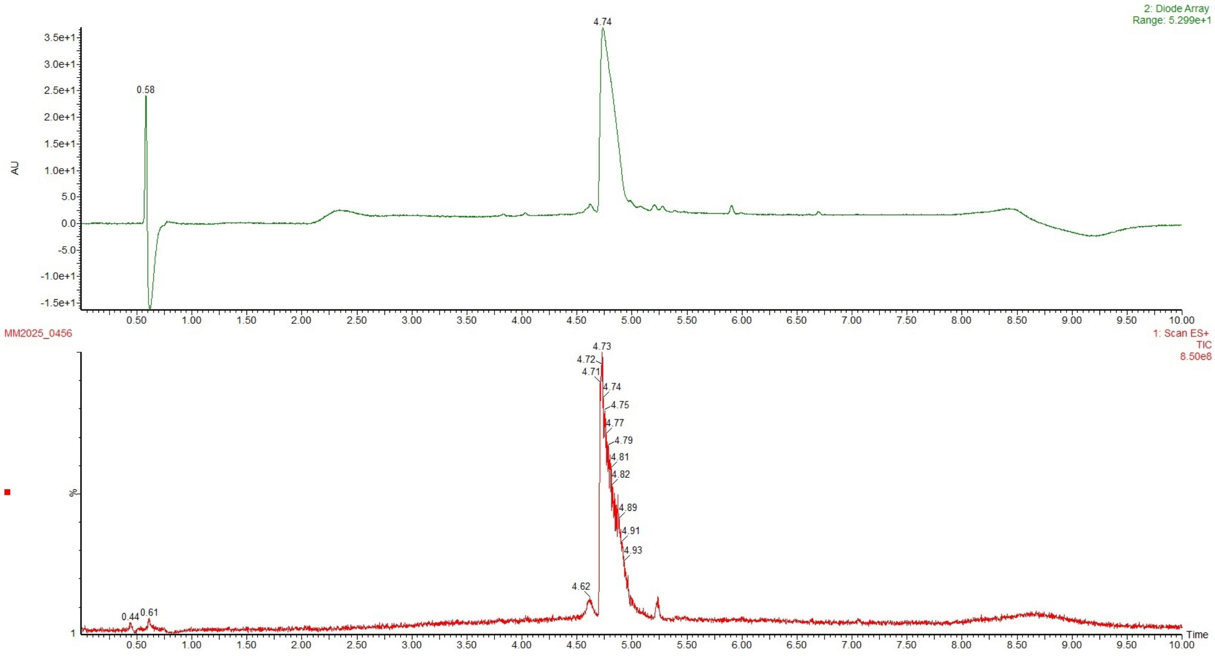

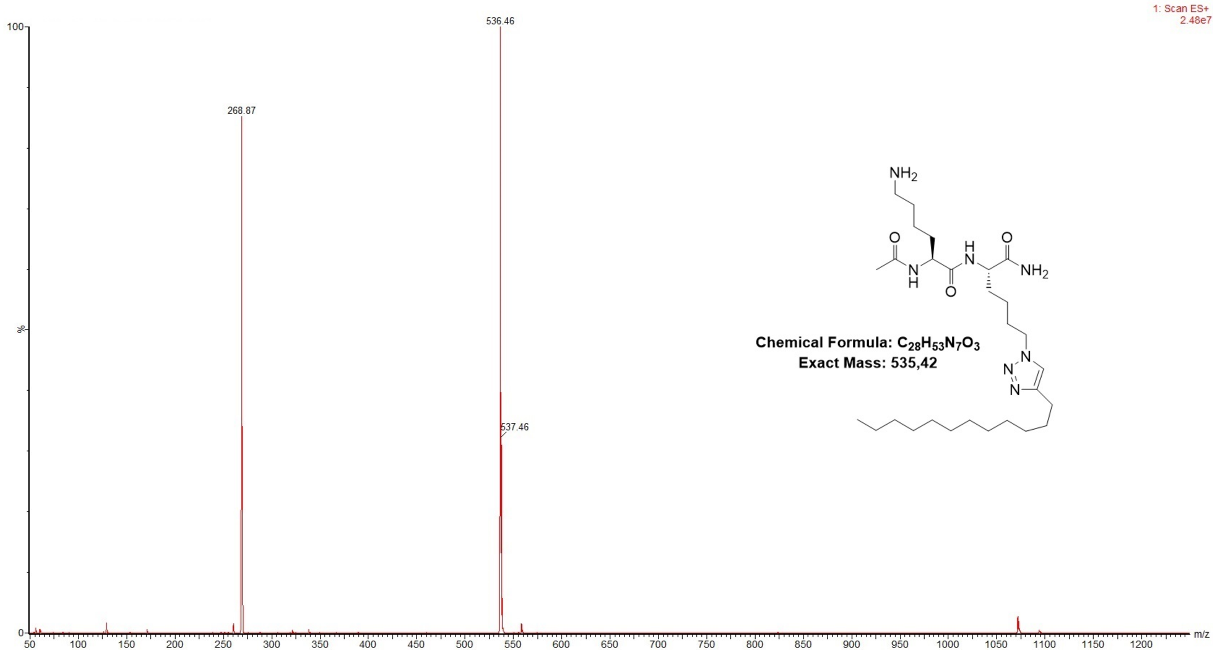

Chemical Formula: $C_{28}H_{53}N_7O_3$
Exact Mass: 535,42

HPLC-MS analysis of compound **LTDi-1b** (**6b**):

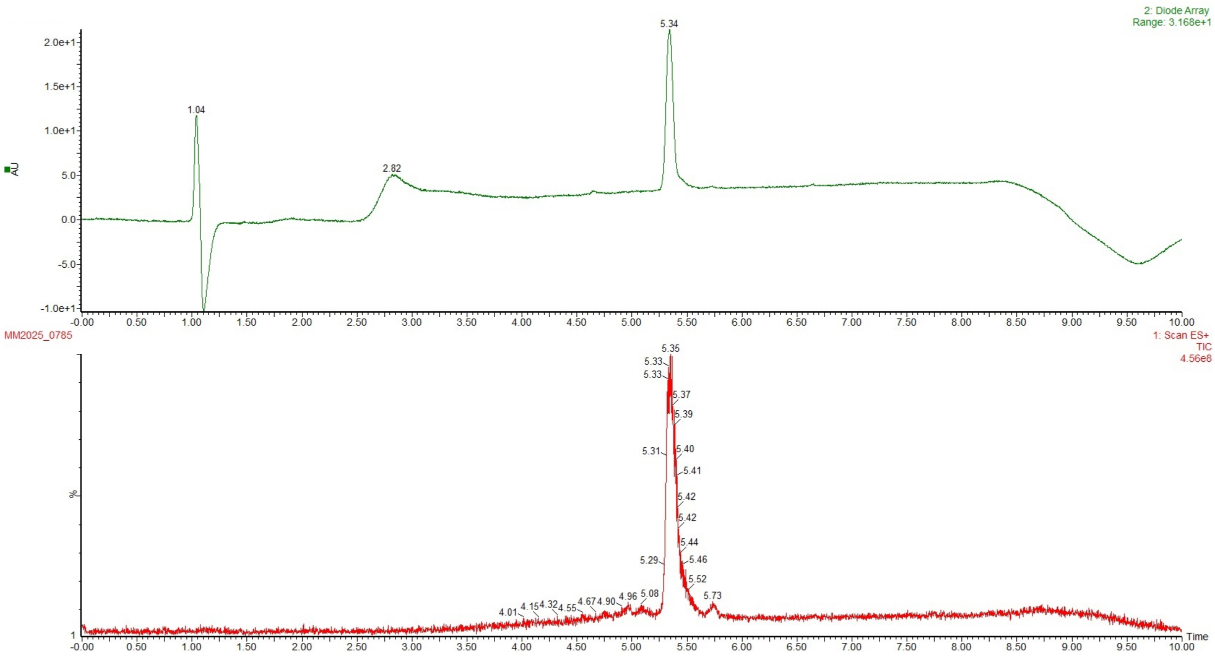

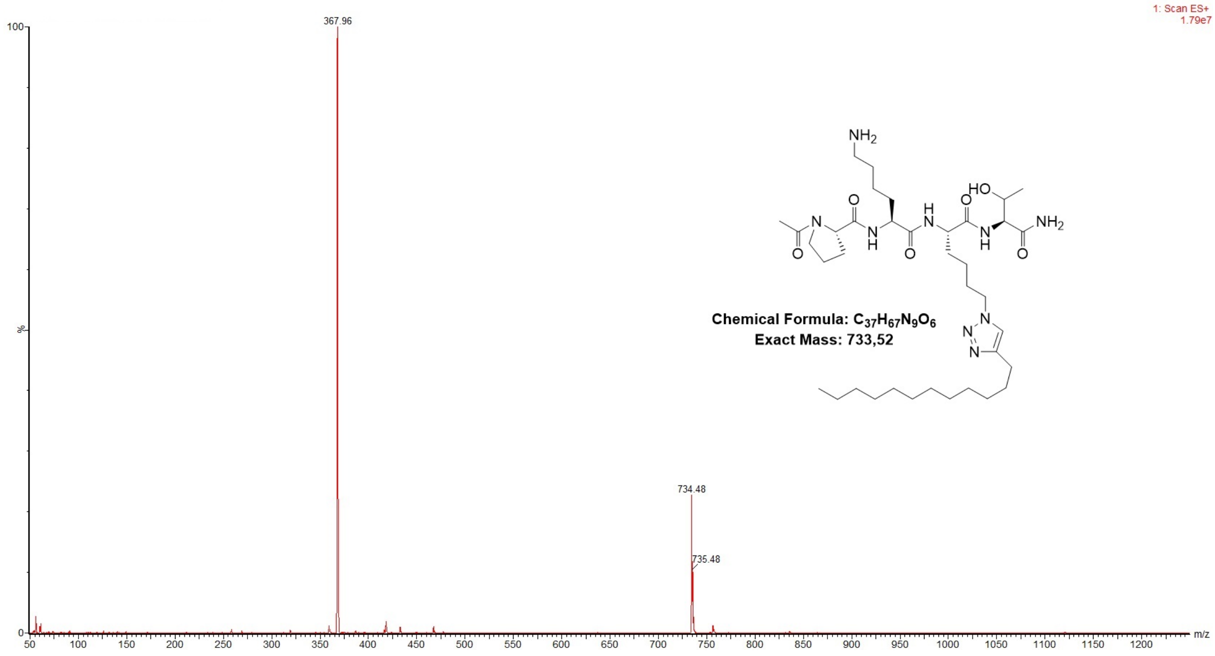

HPLC-MS analysis of compound **BT-6**:

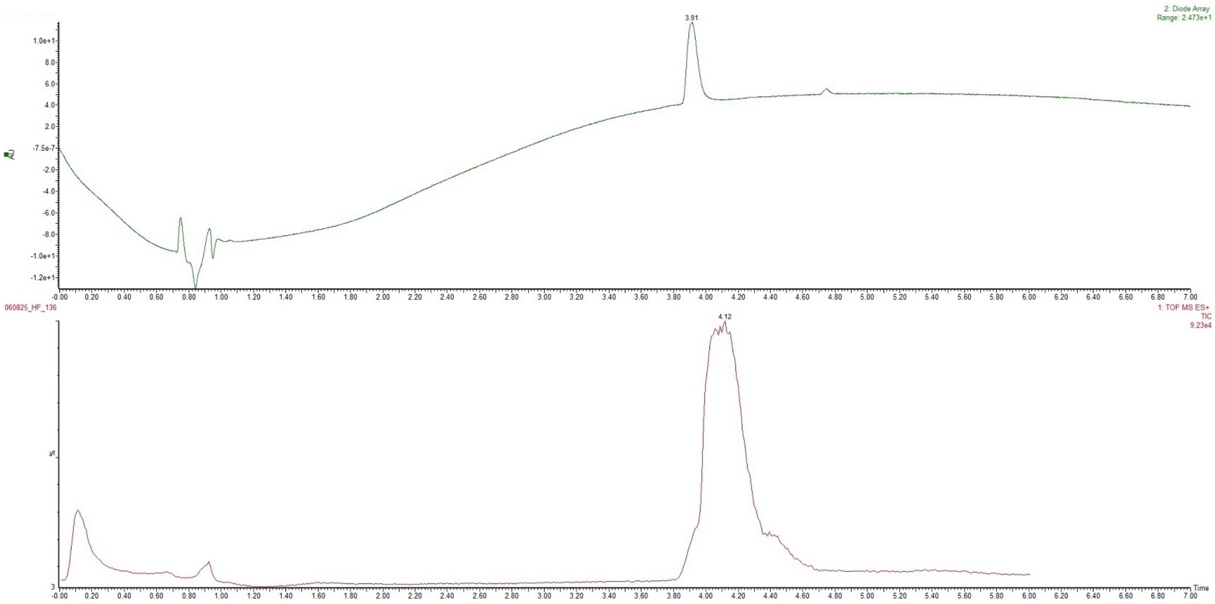

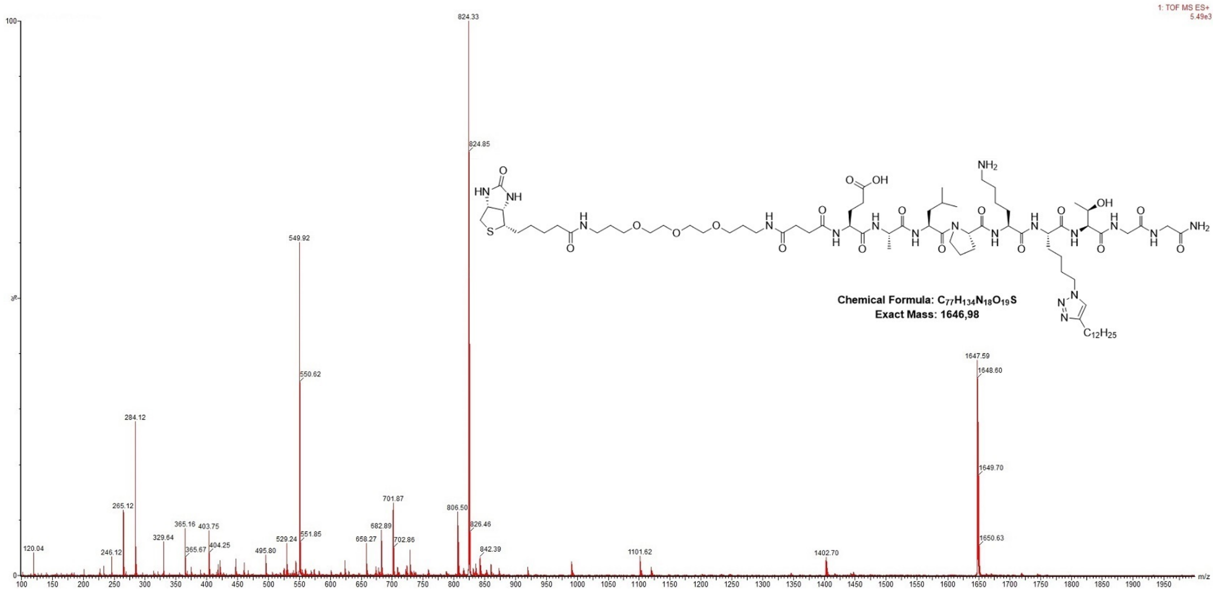

HPLC-MS analysis of compound **BT-6b**:

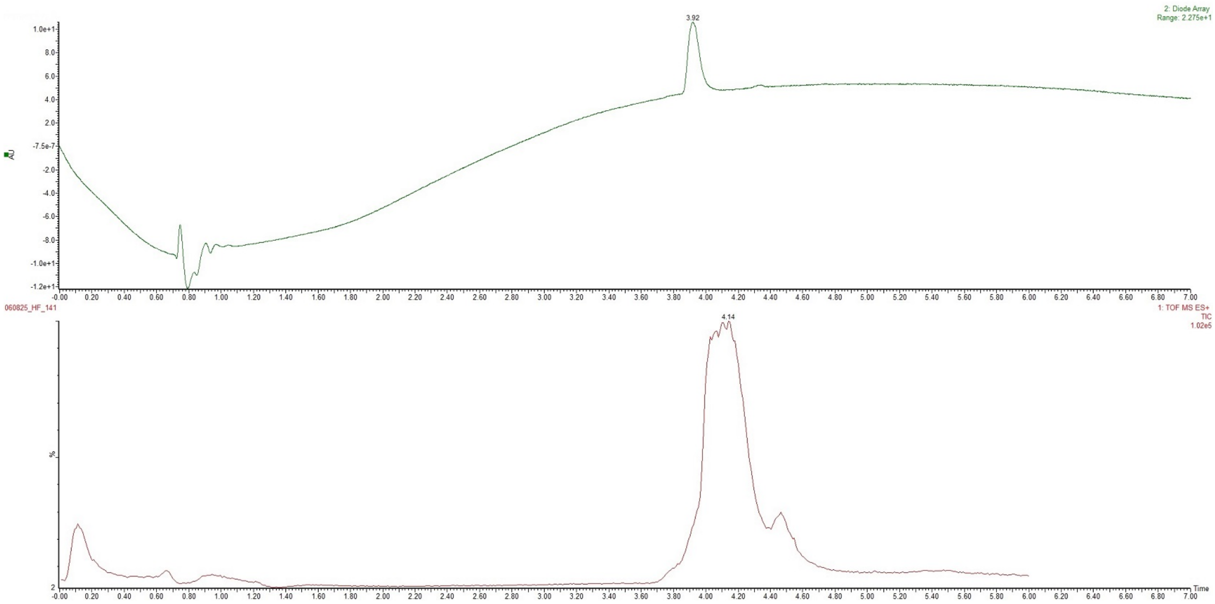

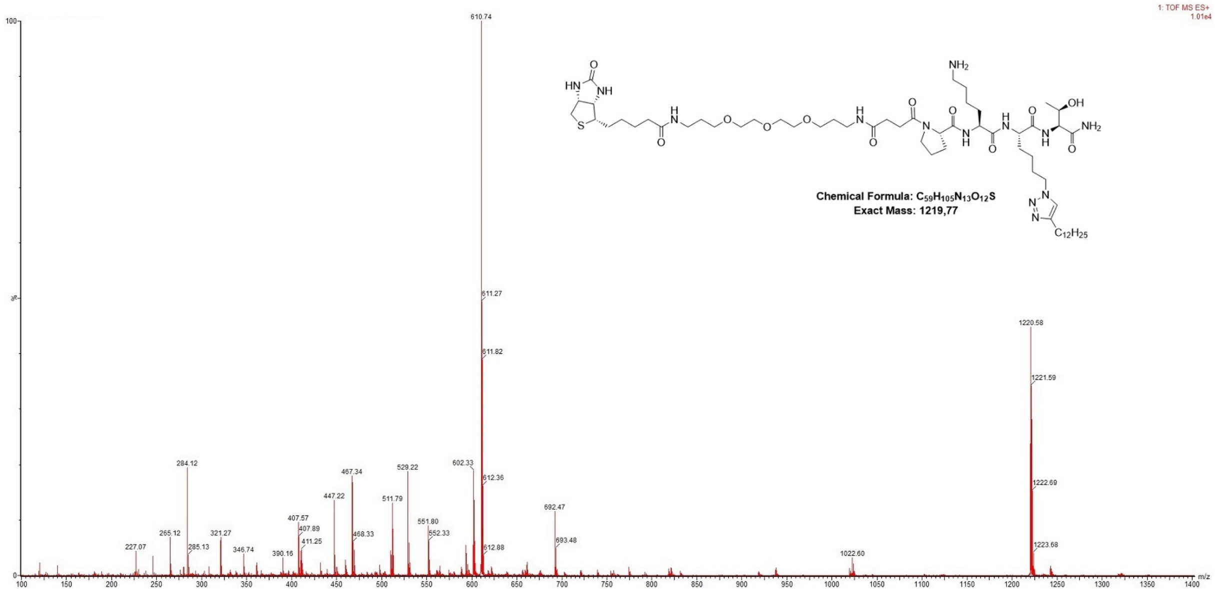

Supplementary References

[1] M. Schiedel, T. Rumpf, B. Karaman, A. Lehotzky, S. Gerhardt, J. Ovádi, W. Sippl, O. Einsle, M. Jung, "Structure-Based Development of an Affinity Probe for Sirtuin 2" *Angew. Chem. Int. Ed.* **2016**, *55*, 2252–2256.

[2] A. Vogelmann, M. Schiedel, N. Wössner, A. Merz, D. Herp, S. Hammelmann, A. Colcerasa, G. Komaniecki, J. Hong, M. Sum, E. Metzger, E. Neuwirt, L. Zhang, O. Einsle, O. Groß, R. Schüle, H. Lin, W. Sippl, M. Jung, "Development of a NanoBRET assay to validate inhibitors of Sirt2-mediated lysine deacetylation and defatty-acylation that block prostate cancer cell migration" *RSC Chem. Biol.* **2022**, *3*, 468–485.

[3] N. A. Spiegelman, J. Y. Hong, J. Hu, H. Jing, M. Wang, I. R. Price, J. Cao, M. Yang, X. Zhang, H. Lin, "A Small-Molecule SIRT2 Inhibitor That Promotes K-Ras4a Lysine Fatty-Acylation" *ChemMedChem* **2019**, *14*, 744–748.

[4] N. Wössner, Z. Alhalabi, J. González, S. Swyter, J. Gan, K. Schmidtkunz, L. Zhang, A. Vaquero, H. Ovaa, O. Einsle, W. Sippl, M. Jung, "Sirtuin 1 Inhibiting Thiocyanates (S1th)—A New Class of Isotype Selective Inhibitors of NAD+ Dependent Lysine Deacetylases" *Front. Oncol.* **2020**, *10*, DOI 10.3389/fonc.2020.00657.

[5] T. Rumpf, M. Schiedel, B. Karaman, C. Roessler, B. J. North, A. Lehotzky, J. Oláh, K. I. Ladwein, K. Schmidtkunz, M. Gajer, M. Pannek, C. Steegborn, D. A. Sinclair, S. Gerhardt, J. Ovádi, M. Schutkowski, W. Sippl, O. Einsle, M. Jung, "Selective Sirt2 inhibition by ligand-induced rearrangement of the active site" *Nat. Commun.* **2015**, *6*, 6263.

[6] F. H. Niesen, H. Berglund, M. Vedadi, "The use of differential scanning fluorimetry to detect ligand interactions that promote protein stability" *Nat. Protoc.* **2007**, *2*, 2212–2221.

[7] S. Swyter, M. Schiedel, D. Monaldi, S. Szunyogh, A. Lehotzky, T. Rumpf, J. Ovádi, W. Sippl, M. Jung, "New chemical tools for probing activity and inhibition of the NAD+-dependent lysine deacylase sirtuin 2" *Philos. Trans. R. Soc. B Biol. Sci.* **2018**, *373*, 20170083.

[8] A. L. Nielsen, N. Rajabi, N. Kudo, K. Lundø, C. Moreno-Yruela, M. Bæk, M. Fontenas, A. Lucidi, A. S. Madsen, M. Yoshida, C. A. Olsen, "Mechanism-based inhibitors of SIRT2: structure–activity relationship, X-ray structures, target engagement, regulation of α-tubulin acetylation and inhibition of breast cancer cell migration" *RSC Chem. Biol.* **2021**, *2*, 612–626.

[9] M. Zessin, M. Meleshin, S. Hilscher, C. Schiene-Fischer, C. Barinka, M. Jung, M. Schutkowski, "Continuous Fluorescent Sirtuin Activity Assay Based on Fatty Acylated Lysines" *Int. J. Mol. Sci.* **2023**, *24*, 7416.

[10] I. Galleano, M. Schiedel, M. Jung, A. S. Madsen, C. A. Olsen, "A Continuous, Fluorogenic Sirtuin 2 Deacylase Assay: Substrate Screening and Inhibitor Evaluation" *J. Med. Chem.* **2016**, *59*, 1021–1031.

[11] D. Qiu, M. S. Wilson, V. B. Eisenbeis, R. K. Harmel, E. Riemer, T. M. Haas, C. Wittwer, N. Jork, C. Gu, S. B. Shears, G. Schaaf, B. Kammerer, D. Fiedler, A. Saiardi, H. J. Jessen, "Analysis of inositol phosphate metabolism by capillary electrophoresis electrospray ionization mass spectrometry" *Nat. Commun.* **2020**, *11*, 6035.

[12] L. Sinatra, A. Vogelmann, F. Friedrich, M. A. Tararina, E. Neuwirt, A. Colcerasa, P. König, L. Toy, T. Z. Yesiloglu, S. Hilscher, L. Gaitzsch, N. Papenkordt, S. Zhai, L. Zhang, C. Romier, O. Einsle, W. Sippl, M. Schutkowski, O. Gross, G. Bendas, D. W. Christianson, F. K. Hansen, M. Jung, M. Schiedel, "Development of First-in-Class Dual Sirt2/HDAC6 Inhibitors as Molecular Tools for Dual Inhibition of Tubulin Deacetylation" *J. Med. Chem.* **2023**, *66*, 14787–14814.

[13] C. Vonrhein, C. Flensburg, P. Keller, A. Sharff, O. Smart, W. Paciorek, T. Womack, G. Bricogne, "Data processing and analysis with the autoPROC toolbox" *Acta Crystallogr. D Biol. Crystallogr.* **2011**, *67*, 293–302.

[14] M. D. Winn, C. C. Ballard, K. D. Cowtan, E. J. Dodson, P. Emsley, P. R. Evans, R. M. Keegan, E. B. Krissinel, A. G. W. Leslie, A. McCoy, S. J. McNicholas, G. N. Murshudov, N. S. Pannu, E. A. Potterton, H. R. Powell, R. J. Read, A. Vagin, K. S. Wilson, "Overview of the CCP4 suite and current developments" *Acta Crystallogr. D Biol. Crystallogr.* **2011**, *67*, 235–242.

[15] A. J. McCoy, R. W. Grosse-Kunstleve, P. D. Adams, M. D. Winn, L. C. Storoni, R. J. Read, "Phaser crystallographic software" *J. Appl. Crystallogr.* **2007**, *40*, 658–674.

[16] J. L. Feldman, K. E. Dittenhafer-Reed, N. Kudo, J. N. Thelen, A. Ito, M. Yoshida, J. M. Denu, "Kinetic and Structural Basis for Acyl-Group Selectivity and NAD+ Dependence in Sirtuin-Catalyzed Deacylation" *Biochemistry* **2015**, *54*, 3037–3050.

[17] G. T. T. Nguyen, S. Schaefer, M. Gertz, M. Weyand, C. Steegborn, "Structures of human sirtuin 3 complexes with ADP-ribose and with carba-NAD+ and SRT1720: binding details and inhibition mechanism" *Acta Crystallogr. D Biol. Crystallogr.* **2013**, *69*, 1423–1432.

[18] P. Emsley, B. Lohkamp, W. G. Scott, K. Cowtan, "Features and development of Coot" *Acta Crystallogr. D Biol. Crystallogr.* **2010**, *66*, 486–501.

[19] G. N. Murshudov, P. Skubák, A. A. Lebedev, N. S. Pannu, R. A. Steiner, R. A. Nicholls, M. D. Winn, F. Long, A. A. Vagin, "REFMAC5 for the refinement of macromolecular crystal structures" *Acta Crystallogr. D Biol. Crystallogr.* **2011**, *67*, 355–367.

[20] P. D. Adams, P. V. Afonine, G. Bunkóczi, V. B. Chen, I. W. Davis, N. Echols, J. J. Headd, L.-W. Hung, G. J. Kapral, R. W. Grosse-Kunstleve, A. J. McCoy, N. W. Moriarty, R. Oeffner, R. J. Read, D. C. Richardson, J. S. Richardson, T. C. Terwilliger, P. H. Zwart, "PHENIX: a comprehensive Python-based system for macromolecular structure solution" *Acta Crystallogr. D Biol. Crystallogr.* **2010**, *66*, 213–221.

[21] V. B. Chen, W. B. Arendall, J. J. Headd, D. A. Keedy, R. M. Immormino, G. J. Kapral, L. W. Murray, J. S. Richardson, D. C. Richardson, "MolProbity: all-atom structure validation for macromolecular crystallography" *Acta Crystallogr. D Biol. Crystallogr.* **2010**, *66*, 12–21.

[22] C. Yung-Chi, W. H. Prusoff, "Relationship between the inhibition constant (*K*I) and the concentration of inhibitor which causes 50 per cent inhibition (*I*50) of an enzymatic reaction" *Biochem. Pharmacol.* **1973**, *22*, 3099–3108.
[23] G. Madhavi Sastry, M. Adzhigirey, T. Day, R. Annabhimoju, W. Sherman, "Protein and ligand preparation: parameters, protocols, and influence on virtual screening enrichments" *J. Comput. Aided Mol. Des.* **2013**, *27*, 221–234.
[24] Schrödinger Release 2021-3: Protein Preparation Wizard; Epik, Schrödinger, LLC, New York, NY, Impact, Schrödinger, LLC, New York, NY, Prime, Schrödinger, LLC, New York, NY, **2021**.
[25] Schrödinger Release 2021-3: Maestro. Schrödinger, LLC, New York, NY. **2021**.
[26] M. P. Jacobson, R. A. Friesner, Z. Xiang, B. Honig, "On the Role of the Crystal Environment in Determining Protein Side-chain Conformations" *J. Mol. Biol.* **2002**, *320*, 597–608.
[27] M. P. Jacobson, D. L. Pincus, C. S. Rapp, T. J. F. Day, B. Honig, D. E. Shaw, R. A. Friesner, "A hierarchical approach to all-atom protein loop prediction" *Proteins Struct. Funct. Bioinforma.* **2004**, *55*, 351–367.
[28] J. C. Shelley, A. Cholleti, L. L. Frye, J. R. Greenwood, M. R. Timlin, M. Uchimaya, "Epik: a software program for pKaprediction and protonation state generation for drug-like molecules" *J. Comput. Aided Mol. Des.* **2007**, *21*, 681–691.
[29] D. Shivakumar, J. Williams, Y. Wu, W. Damm, J. Shelley, W. Sherman, "Prediction of Absolute Solvation Free Energies using Molecular Dynamics Free Energy Perturbation and the OPLS Force Field" *J. Chem. Theory Comput.* **2010**, *6*, 1509–1519.
[30] W. L. Jorgensen, D. S. Maxwell, J. Tirado-Rives, "Development and Testing of the OPLS All-Atom Force Field on Conformational Energetics and Properties of Organic Liquids" *J. Am. Chem. Soc.* **1996**, *118*, 11225–11236.
[31] W. L. Jorgensen, J. Tirado-Rives, "The OPLS [optimized potentials for liquid simulations] potential functions for proteins, energy minimizations for crystals of cyclic peptides and crambin" *J. Am. Chem. Soc.* **1988**, *110*, 1657–1666.
[32] D. A. Case, H. M. Aktulga, K. Belfon, D. S. Cerutti, G. A. Cisneros, V. W. D. Cruzeiro, N. Forouzesh, T. J. Giese, A. W. Götz, H. Gohlke, S. Izadi, K. Kasavajhala, M. C. Kaymak, E. King, T. Kurtzman, T.-S. Lee, P. Li, J. Liu, T. Luchko, R. Luo, M. Manathunga, M. R. Machado, H. M. Nguyen, K. A. O'Hearn, A. V. Onufriev, F. Pan, S. Pantano, R. Qi, A. Rahnamoun, A. Risheh, S. Schott-Verdugo, A. Shajan, J. Swails, J. Wang, H. Wei, X. Wu, Y. Wu, S. Zhang, S. Zhao, Q. Zhu, T. E. Cheatham, D. R. Roe, A. Roitberg, C. Simmerling, D. M. York, M. C. Nagan, K. M. Merz, "AmberTools" *J. Chem. Inf. Model.* **2023**, *63*, 6183–6191.
[33] J. A. Maier, C. Martinez, K. Kasavajhala, L. Wickstrom, K. E. Hauser, C. Simmerling, "ff14SB: Improving the Accuracy of Protein Side Chain and Backbone Parameters from ff99SB" *J. Chem. Theory Comput.* **2015**, *11*, 3696–3713.
[34] P. Li, B. P. Roberts, D. K. Chakravorty, K. M. Jr. Merz, "Rational Design of Particle Mesh Ewald Compatible Lennard-Jones Parameters for +2 Metal Cations in Explicit Solvent" *J. Chem. Theory Comput.* **2013**, *9*, 2733–2748.
[35] P. Li, K. M. Jr. Merz, "Taking into Account the Ion-Induced Dipole Interaction in the Nonbonded Model of Ions" *J. Chem. Theory Comput.* **2014**, *10*, 289–297.
[36] P. Li, L. F. Song, K. M. Jr. Merz, "Parameterization of Highly Charged Metal Ions Using the 12-6-4 LJ-Type Nonbonded Model in Explicit Water" *J. Phys. Chem. B* **2015**, *119*, 883–895.
[37] P. Li, L. F. Song, K. M. Jr. Merz, "Systematic Parameterization of Monovalent Ions Employing the Nonbonded Model" *J. Chem. Theory Comput.* **2015**, *11*, 1645–1657.
[38] A. Jakalian, D. B. Jack, C. I. Bayly, "Fast, efficient generation of high-quality atomic charges. AM1-BCC model: II. Parameterization and validation" *J. Comput. Chem.* **2002**, *23*, 1623–1641.
[39] J. Wang, R. M. Wolf, J. W. Caldwell, P. A. Kollman, D. A. Case, "Development and testing of a general amber force field" *J. Comput. Chem.* **2004**, *25*, 1157–1174.
[40] W. L. Jorgensen, J. Chandrasekhar, J. D. Madura, R. W. Impey, M. L. Klein, "Comparison of simple potential functions for simulating liquid water" *J. Chem. Phys.* **1983**, *79*, 926–935.
[41] N. Vithani, S. Zhang, J. P. Thompson, L. A. Patel, A. Demidov, J. Xia, A. Balaeff, A. Mentes, Y. A. Arnautova, A. Kohlmann, J. D. Lawson, A. Nicholls, A. G. Skillman, D. N. LeBard, "Exploration of Cryptic Pockets Using Enhanced Sampling Along Normal Modes: A Case Study of KRAS G12D" *J. Chem. Inf. Model.* **2024**, *64*, 8258–8273.
[42] Y.-H. Sanejouand, "At least three xenon binding sites in the glycine binding domain of the N-methyl D-aspartate receptor" *Arch. Biochem. Biophys.* **2022**, *724*, 109265.
[43] O. Warr, C. J. Ballentine, J. Mu, A. Masters, "Optimizing Noble Gas–Water Interactions via Monte Carlo Simulations" *J. Phys. Chem. B* **2015**, *119*, 14486–14495.
[44] J.-P. Ryckaert, G. Ciccotti, H. J. C. Berendsen, "Numerical Integration of the Cartesian Equations of Motion of a System with Constraints: Molecular Dynamics of n-Alkanes" *J. Comput. Phys.* **1977**, *23*, 327–341.
[45] T. Darden, D. York, L. Pedersen, "Particle mesh Ewald: An $N \cdot \log(N)$ method for Ewald sums in large systems" *J. Chem. Phys.* **1993**, *98*, 10089–10092.
[46] U. Essmann, L. Perera, M. L. Berkowitz, T. Darden, H. Lee, L. G. Pedersen, "A smooth particle mesh Ewald method" *J. Chem. Phys.* **1995**, *103*, 8577–8593.
[47] D. R. Roe, T. E. I. Cheatham, "PTRAJ and CPPTRAJ: Software for Processing and Analysis of Molecular Dynamics Trajectory Data" *J. Chem. Theory Comput.* **2013**, *9*, 3084–3095.
[48] Schrödinger Release 2021-3: Desmond Molecular Dynamics System, D. E. Shaw Research, New York, NY, 2021. Maestro-Desmond Interoperability Tools, Schrödinger, New York, NY **2021**.

[49] P. Schmidtke, A. Bidon-Chanal, F. J. Luque, X. Barril, “MDpocket: open-source cavity detection and characterization on molecular dynamics trajectories” *Bioinformatics* **2011**, *27*, 3276–3285.
[50] V. Le Guilloux, P. Schmidtke, P. Tuffery, “Fpocket: An open source platform for ligand pocket detection” *BMC Bioinformatics* **2009**, *10*, 168.
[51] PyMOL Molecular Graphics System, Version 3.1.0. Schrödinger, LLC., “PyMOL Molecular Graphics System, Version 3.1.0. Schrödinger, LLC.” **2024**.
[52] J. D. Hunter, “Matplotlib: A 2D Graphics Environment” *Comput. Sci. Eng.* **2007**, *9*, 90–95.
[53] M. Waskom, “seaborn: statistical data visualization” *J. Open Source Softw.* **2021**, *6*, 3021.
[54] W. Gai, H. Li, H. Jiang, Y. Long, D. Liu, “Crystal structures of SIRT3 reveal that the α2-α3 loop and α3-helix affect the interaction with long-chain acyl lysine” *FEBS Lett.* **2016**, *590*, 3019–3028.
[55] S. Jana, J. Shang, J. Y. Hong, M. K. Fenwick, R. Puri, X. Lu, A. M. Melnick, M. Li, H. Lin, “A Mitochondria-Targeting SIRT3 Inhibitor with Activity against Diffuse Large B Cell Lymphoma” *J. Med. Chem.* **2024**, *67*, 15428–15437.
[56] Y. Wang, Y. M. E. Fung, W. Zhang, B. He, M. W. H. Chung, J. Jin, J. Hu, H. Lin, Q. Hao, “Deacylation Mechanism by SIRT2 Revealed in the 1′-SH-2′-O-Myristoyl Intermediate Structure” *Cell Chem. Biol.* **2017**, *24*, 339–345.
